# Photostable π-Expanded Furans via Base-Mediated Quinone Oxygen-Annulation

**DOI:** 10.1021/acs.joc.5c02906

**Published:** 2026-02-10

**Authors:** Abhishek Pareek, Maja Morawiak, Emran Masoumifeshani, Przemysław Gaweł

**Affiliations:** 49559 Institute of Organic Chemistry, Polish Academy of Sciences, Kasprzaka 44/52, 01-224 Warsaw, Poland

## Abstract

A one-pot oxygen-annulation couples chloroquinones with aromatic alcohols to efficiently construct π-expanded furans in yields up to 91%. Subsequent TIPS-acetylene functionalization and reductive aromatization enhance solubility and furnish bench-stable chromophores that absorb in the blue and exhibit strong fluorescence. Time-dependent density functional theory (TD-DFT) reveals low-energy, charge-transfer excitations in the quinone intermediates, which shift to higher-energy, localized π–π* transitions upon aromatization. Remarkably, these π-expanded furans withstand UV irradiation over 10-fold longer than TIPS-pentacene, highlighting their exceptional photostability.

## Graphical abstract

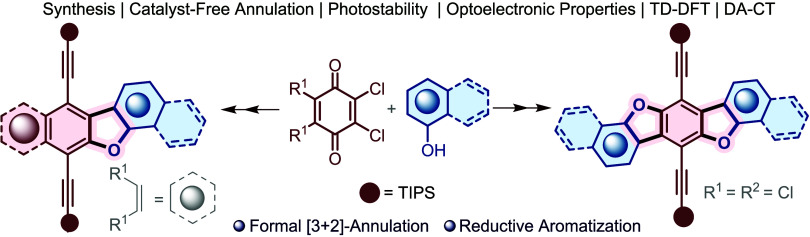

## Introduction

Organic electronics is reshaping modern materials science by enabling devices that are not only lightweight and flexible but also compatible with solution-based processing.^1–3^ Central to this progress are heteroatom-containing polycyclic aromatic compounds (PACs), which offer precise control over molecular electronics through strategic incorporation of elements such as nitrogen, oxygen, or sulfur (Figure 1).^4,5^ These modifications influence key functional properties, such as redox potentials, charge mobility, and exciton dynamics, making PACs essential in a broad spectrum of applications, including organic photovoltaics (OPVs),^6,7^ organic field-effect transistors (OFETs),^8,9^ organic phototransistors (OPTs),^10^ and organic light-emitting diodes (OLEDs).^11^

**Figure 1:**
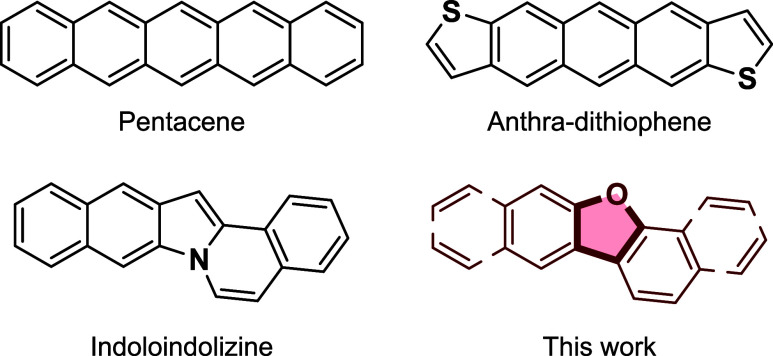
Examples of PACs featuring carbon, sulfur, nitrogen, and oxygen-based motifs, including those presented in this work.

Incorporating electron-rich heterocycles into the framework of PACs enables precise modulation of frontier-orbital energies, enhanced intramolecular charge-transfer, and stronger, directional π-stacking interactions, features that collectively lower the transport barrier, boost charge-carrier mobility, and raise device efficiencies in OFETs, OLEDs, and OPVs.^12^ While sulfur- and nitrogen-doped PACs have been extensively studied and widely applied, oxygen-doped analogues remain comparatively underexplored.^13,14^ Among these, furan stands out due to its electron-rich nature, natural abundance, and thus, the potential for enhanced sustainability and biodegradability.^15–18^ π-Expanded furan derivatives, as well as furan-based conjugated polymers, have demonstrated promising performance in OLEDs, OFETs, and OPVs.^19–26^ However, further exploration of this class of materials has been hampered by the limited number of broadly applicable and efficient synthetic strategies for constructing π-expanded furan scaffolds.^27–29^

Motivated by the need for new design strategies to access heteroatom-rich PACs with tailored optoelectronic properties, we developed a concise, metal-free, efficient, and modular synthetic approach for embedding furan units into laterally π-expanded frameworks. Our approach utilizes the rich reactivity of quinones to construct furan-embedded polyaromatic cores through a key oxygen-annulation step. This methodology builds upon the classical protocol reported by Liebermann and subsequent studies by others, while substantially expanding the substrate scope and synthetic utility.^30–34^ It is further inspired by our recent work on indoloindolizines, extending the underlying synthetic principles to oxygen-doped analogues and thereby broadening the structural and electronic diversity of accessible π-conjugated architectures.^35^ Our protocol allows for the construction of both symmetrical and unsymmetrical π-expanded furan derivatives comprising 5 to 7 fused rings. The resulting compounds are bench-stable, crystalline, and exhibit strong fluorescence, making them promising candidates for future applications in organic optoelectronics.

## Results and Discussion

### Synthesis

Our retrosynthetic approach centers on the construction of a furan-fused polycyclic core via strategic cleavage and annulation, converging on a key naphthoquinone intermediate (Table 1). In the model transformation, 2,3-dichloro-1,4-naphthoquinone **1a** was submitted to reaction with 2-naphthol **2a** in pyridine, which serves as both base and solvent, at 105 °C for 24 h. This high-yielding reaction produces furan-embedded pentacyclic quinone **3a**, which has poor solubility in common organic solvents. Nevertheless, the excellent efficiency of this transformation allows for straightforward isolation of the product by simple filtration, eliminating the need for chromatographic purification. To investigate the scope of this reaction and alternate the π-system, we first tested different aromatic alcohols and found that both 1-naphthol **2d** and 2-hydroxyanthracene **2e** give the corresponding polycyclic furans in good yields (Table 1). However, attempts to synthesize a simpler tetracyclic system under standard conditions using **1a** and phenol led only to phenolic OH substitution on quinone, without further cyclization to furan.^36^ For reference, we prepared this tetracyclic quinone **3h** using alternative Pd-catalyzed protocol.^37^ Derivatives of 2-naphthol functionalized with electron-withdrawing (Br, **2b**) and electron-donating (OMe, **2c**) groups worked fine as well, showing good versatility of this transformation throughout larger aromatic alcohols. To expand the π-system on the quinone side we used 2,3-dichloro-1,4-anthraquinone **1b**,^38^ which reacted readily with both 2-naphthol **2a** and 1-naphthol **2d** giving corresponding furans **3e** and **3f**, respectively (Table 1).

**Table 1:**
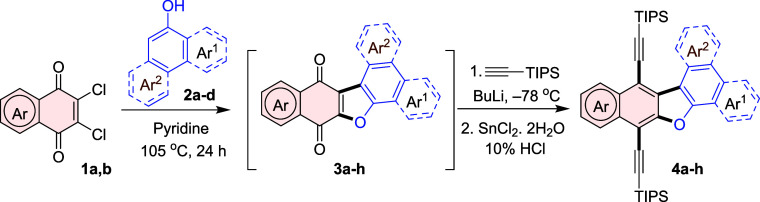

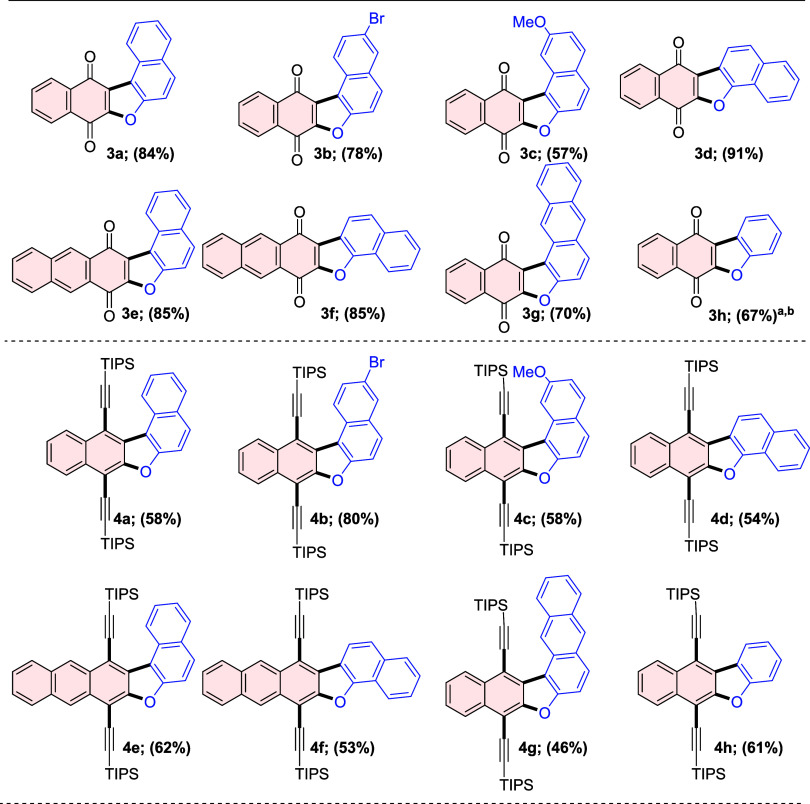
Synthesis of Unsymmetrical π-Expanded Furan-Embedded Quinones **3a–h** and Aromatized Derivatives **4a–h**

a 2-Chloro-1,4-naphthoquinone was used instead of 2,3-dichloro-1,4-naphthoquinone.

b Reaction conditions for the synthesis of **3h** are as reported in ref 37.

Further functionalization by addition of lithium triisopropylsilyl (TIPS)-acetylide to quinones **3a–h** gave the corresponding propargylic alcohol intermediates, which were transformed *in situ* into π-expanded furans **4a–h** via reductive elimination using aqueous SnCl_2_ in 10% HCl (Table 1). The incorporation of TIPS groups significantly improves the solubility of compounds **4** compared to their quinone precursors **3**, thereby enabling purification by chromatographic methods and facilitating full spectroscopic characterization.^39^

On the basis of literature on 2-naphthol reactivity and the control experiments described in Section S3,^40–43^ we propose the mechanism depicted in Figure S1 for the key oxygen-annulation step. In pyridine, 2-naphthol is supposedly in equilibrium with a small amount of naphtholate as an ion-paired species. Additionally, pyridine can enhance nucleophilicity through hydrogen bonding. Within the naphtholate resonance manifold, the negative charge is delocalized over the O/C framework, imparting partial C-nucleophilic character at the α-position. This naphtholate/naphtholate-like nucleophile then attacks the electrophilic C-3 position of 2,3-dichloro-1,4-naphthoquinone **1a**, leading to the formation of an adduct via a base-assisted addition–elimination pathway. The resulting phenoxide oxygen then intramolecularly attacks the adjacent C-2 center of quinone, displacing the second chloride and making the new C–O bond that closes the five-membered ring. Subsequent proton transfer and elimination restore aromaticity, delivering the rigid, π-expanded furan-fused quinone. Our control studies confirm that all three components: elevated temperature (∼105 °C), nucleophilic 2-naphthol, and pyridine acting as base, solvent, and HCl scavenger are indispensable. Only under this specific combination of conditions does the annulation proceed efficiently, producing the desired furan-embedded polycyclic framework in high yield.

After successfully synthesizing unsymmetrically π-expanded furans **4a–h**, we then explored the synthesis of symmetrical π-extension. To this end, we replaced **1a** with chloranil **1c** as we expected its four electrophilic sites to lead to the formation of symmetric polycyclic furans upon reaction with two equivalents of naphthols. The initial reaction with 1-naphthol **2d** resulted in the isolation of the desired quinone **3i** in good yield (Table 2). Similarly, reactions with **2a** and **2c** led to the isolation of corresponding quinones in reasonable yields. Subsequent TIPS-acetylene functionalization gave π-expanded furans **4i–k**.

**Table 2:**
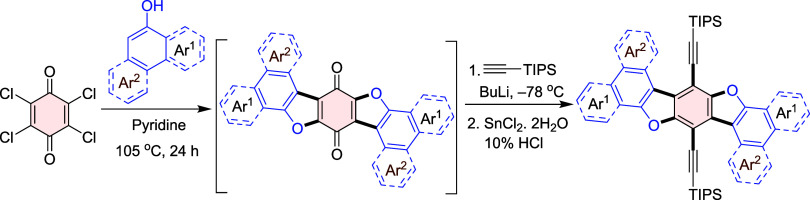

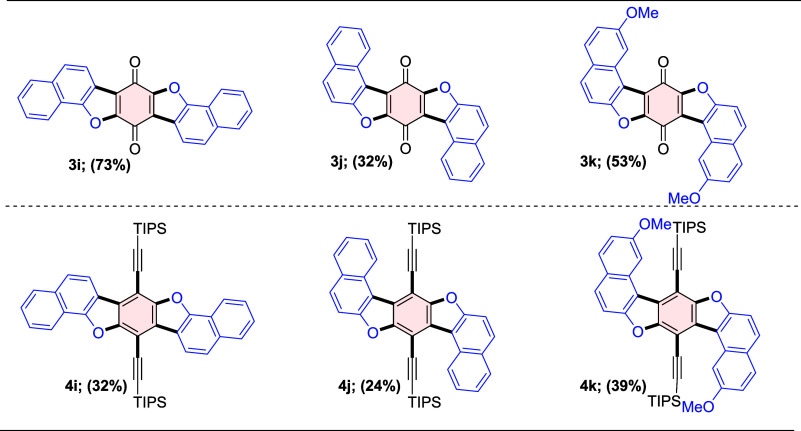
Synthesis of Symmetrical π-Expanded Furan-Embedded Quinones **3i–k** and Aromatized Derivatives **4i–k**

### Structure

The structure of **4i** was unambiguously confirmed through the single-crystal X-ray diffraction analysis (Figure 2). Crystal was grown by slow diffusion of hexanes into a CH_2_Cl_2_ solution of compound **4i**. In the crystal, the molecules arrange in a slipped-stacked parallel domains that resemble a brickwork-type packing of TIPS-pentacene.^39^ Packing is dominated by π–π interactions, and the separation between adjacent π-expanded furan backbones is as short as 3.5 Å, indicating substantial intermolecular overlap.

**Figure 2:**
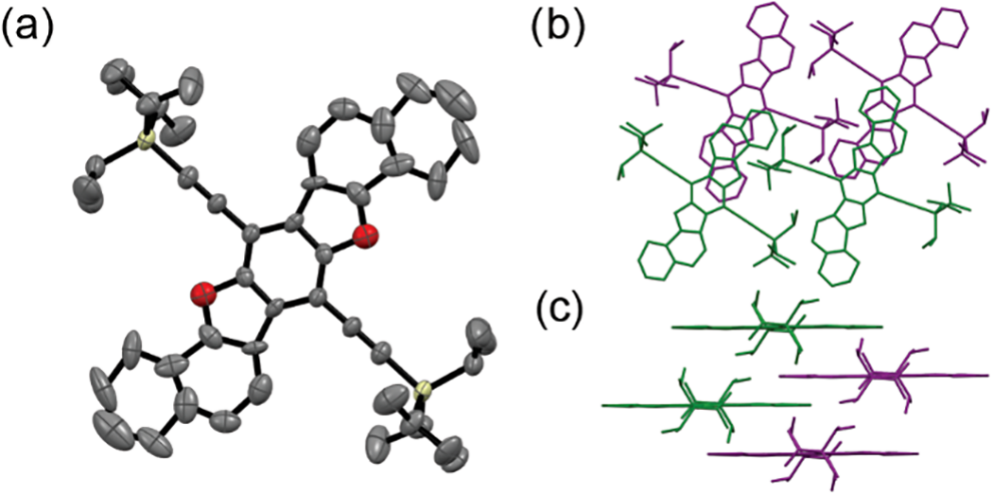
(a) ORTEP plot for the single crystal structure of **4i**. Atomic displacement parameters at 296 K are drawn at the 30% probability level. Atom colors: gray, C; red, O; and yellow, Si. (b) Top and (c) side views of the slipped-stack packing. H atoms have been omitted for the sake of clarity.

### Photophysical Properties

The photophysical behavior of the π-expanded furans **4** mirrors the size and substitution pattern of their polycyclic cores. Visually, the series spans pale yellow to deep orange solids that are strongly fluorescent in both CH_2_Cl_2_ solution and in the solid state (Figure 3 and Table 3). The smallest derivative, the four-ring system **4h**, absorbs up to λ_max_ = 395 nm and emits at 403 nm with a solution fluorescence quantum yield (Φ_PL_) of 33%. Incremental π-extension on the benzofuran side produces progressive bathochromic shifts: an additional ring in **4d** moves the band by ≈10 nm, while in **4a** shift it by ≈20 nm both in absorption and fluorescence spectra. Substitution of **4a** with Br at position 3 (**4b**) leaves λ_max_ essentially unchanged, whereas a methoxy group at position 2 in **4c** narrows the gap only marginally in absorption but red-shifts the emission by almost 30 nm, an effect attributable to the electron-donating character of MeO combined with steric strain imposed by repulsion between the MeO and TIPS groups. Further π-extension has a more dramatic impact. Replacing the naphthalene unit with an anthracene in **4g** shifts λ_max_ to 455 nm and the emission to 493 nm. Expansion on the opposite (former quinone) side is even more effective: the fused polycyclic frameworks **4e** and **4f** reach absorption onsets at 486 and 474 nm, respectively. Although these red-shifts track the expected reduction in the HOMO–LUMO gap, the transition energies remain higher than those of fully conjugated acenes of comparable size. Two-dimensional NICS maps (Section S9) reveal the origin of this offset: the furan bridge partially insulates the adjoining benzenoid circuits, creating two quasi-independent chromophores within a single π-system, a phenomenon we also observed in our recent indoloindolizine study.^35^ This electronic isolation offers a useful handle for fine-tuning optical gaps without excessive red-shifting. The unsymmetrically π-expanded furans **4a–h** are all emissive, with Φ_PL_ ranging from 15% to 66% and generally small Stokes shifts, reflecting the rigidity of their structures in excited states (Table 3). While benzofuran derivatives are typically considered highly emissive, the emission efficiencies observed in this study are comparable to those of previously reported π-expanded benzofurans.^44,45^ The symmetrically π-expanded derivatives **4i–k** absorb and emit in similar spectral window; however, introducing a peripheral methoxy group in **4k** produces an additional bathochromic shift of ∼14 nm in emission band relative to the parent compound **4j**, consistent with the stronger electron-donating character of the MeO substituent (Section S4).

**Figure 3:**
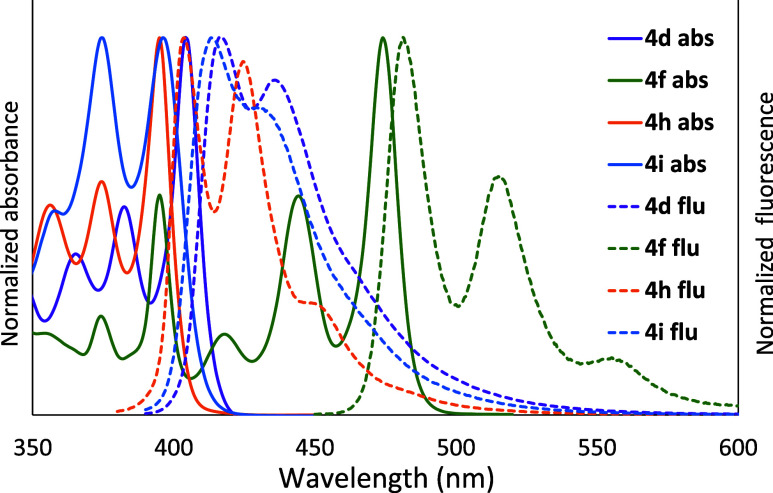
Normalized UV–vis absorption and fluorescence spectra of selected π-expanded furans: **4d**, **4f**, **4h**, and **4i**.

**Table 3:** Photophysical and Computational Data for **4a–k**

	UV–vis	fluorescence			DFT
Cpd. code	λ_abs_, nm	λ_em_, nm	Stokes shift, eV	Φ_PL_	Δ*E*_TDDFT_, eV
**4a**	416	420	0.03	0.16	3.26
**4b**	418	428	0.07	0.15	3.26
**4c**	416	448	0.21	0.52	3.26
**4d**	404	414	0.08	0.48	3.34
**4e**	486	495	0.05	0.24	2.79
**4f**	474	481	0.04	0.36	2.84
**4g**	455	493	0.21	0.66	3.06
**4h**	395	403	0.07	0.33	3.43
**4i**	396	414	0.13	0.55	3.50
**4j**	411	428	0.12	0.48	3.37
**4k**	422	442	0.13	0.50	3.30

### Intramolecular Charge-Transfer

An interesting photophysical trend emerged when the furan-fused naphthoquinone **3a** was converted to the fully aromatized derivative **4a** (Figure 4a). Despite the formal extension of conjugation, the lowest-energy absorption shifted hypsochromically (blue) rather than bathochromically. The same behavior was observed throughout the entire series of π-expanded furans **3** and **4** (Section S4). TD-DFT analysis explains this behavior: in the quinones **3**, the S_0_ → S_1_ transition is strongly charge-transfer (CT) in nature, with quinone moiety serving as a strong electron-accepting unit and naphthofuran as electron-donor (Figure 5 and Section S8). Excited-state aromatic stabilization within the quinone drives the charge-transfer and lowers the optical gap, yielding bathochromically shifted absorption.^46,47^ In contrast, the fully reduced compounds **4a–h** display conventional π–π* transitions that are more localized, restoring a larger HOMO–LUMO gap and thus a blue-shifted spectrum (Section S8).

**Figure 4:**
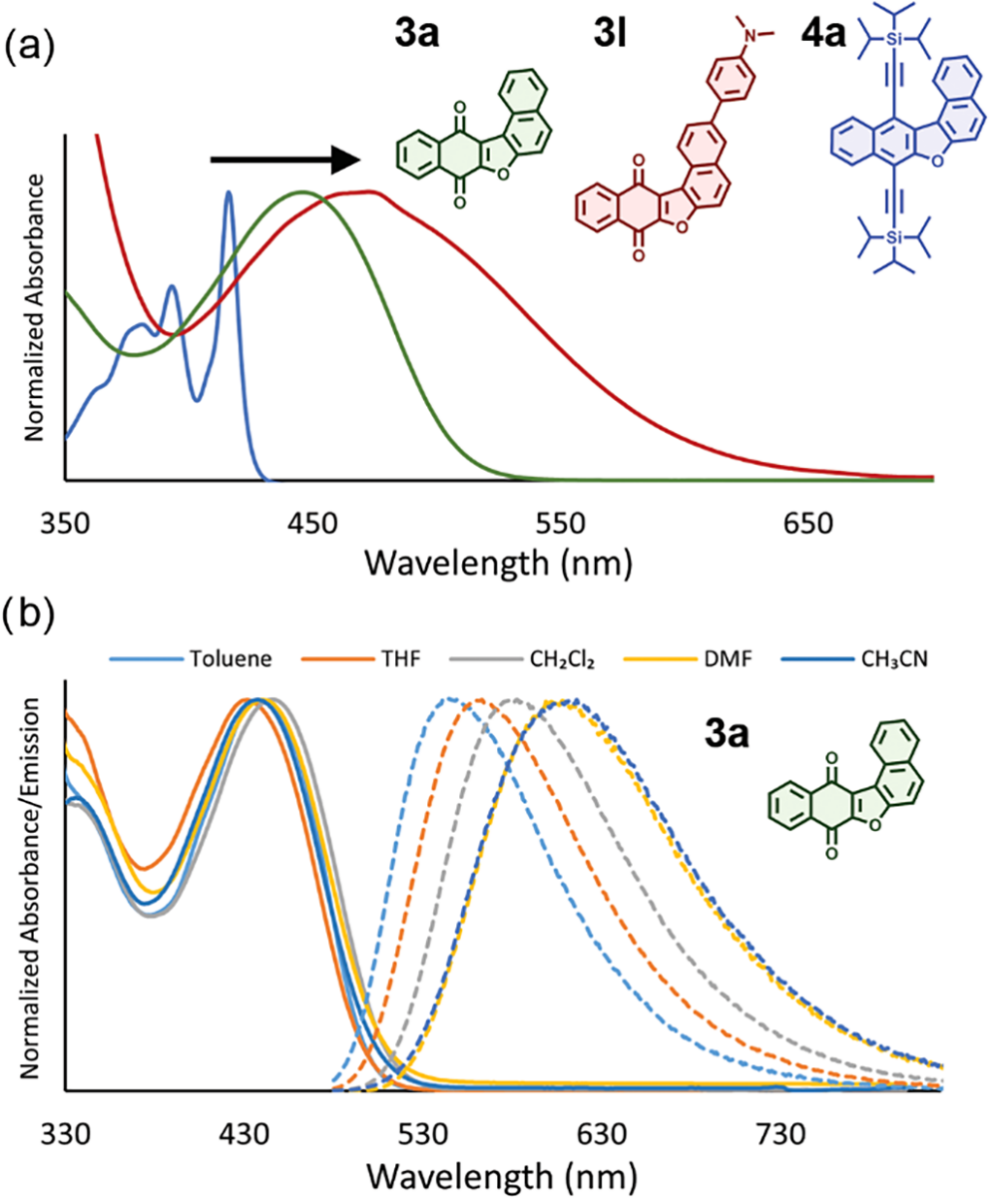
(a) UV–vis absorption spectra of **4a**, **3a**, and **3l** and (b) absorption and emission spectra of compound **3a** recorded in different solvents (toluene, THF, CH_2_Cl_2_, CH_3_CN, and DMF).

**Figure 5:**
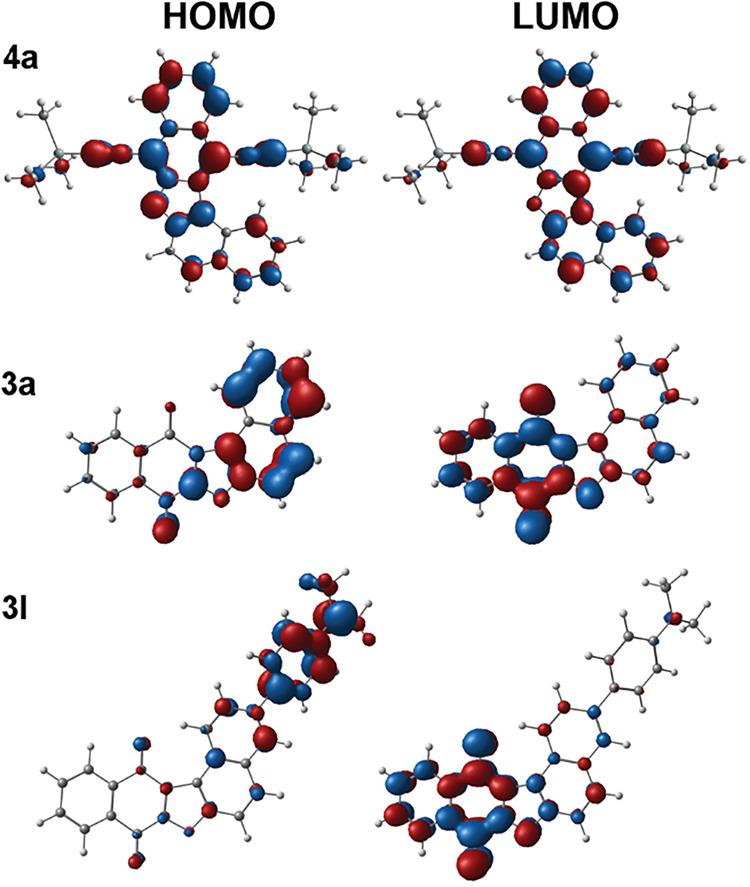
Frontier molecular orbitals of **4a**, **3a**, and **3l**. CAM-B3LYP^48^/def2-TZVP^49^ level of theory. Isovalues at 0.02.

To amplify the CT effect we appended a powerful donor, *N*,*N*-dimethylaniline, to the naphthofuran terminus of **3b** via Suzuki coupling, affording **3l**. The new derivative exhibits a broad absorption tailing to around 600 nm, markedly red-shifted relative to its parent compound **3a**. Frontier molecular orbital plots confirm that the HOMO is centered on the *N*,*N*-dimethylaniline unit, whereas the LUMO is delocalized over the quinone, validating the intensified CT donor-to-acceptor transition responsible for the bathochromic shift (Figure 5).

To further probe the CT character of S_0_ → S_1_ transition in **3a**, we examined its solvatochromic behavior by recording absorption and emission spectra in solvents of varying polarity (toluene, THF, CH_2_Cl_2_, DMF, and CH_3_CN) (Figure 4b). The absorption spectra remained nearly identical across all tested solvents, indicating negligible perturbation of the dipole moment between the ground state and Franck–Condon excited state. In contrast, the emission spectra displayed a pronounced bathochromic shift with increasing solvent polarity, consistent with stabilization of a polar, intramolecular charge-transfer (ICT) excited state. This solvatochromic response provides strong experimental support for the calculated spatial separation between the HOMO and LUMO in **3a**. For the dimethylaniline-substituted analogue **3l**, fluorescence was weak and solvent-dependent spectral variations were inconsistent, precluding reliable determination of a solvatochromic trend.

### Stability

π-Extended furan frameworks have long been viewed as too photolabile for practical optoelectronic use, yet our results show that careful molecular design can deliver impressive photostability.^50,51^ To support that, we subjected aerated CH_2_Cl_2_ solutions of **4d**, **4f**, **4h**, and **4i** to continuous 254 nm irradiation from a 4 W low-pressure mercury lamp and tracked the decay of absorption bands (Section S5). A clear size-stability relationship emerged. The tetracyclic derivative **4h** proved the most robust, retaining half of its initial absorbance for nearly 6 h under these harsh conditions. The pentacyclic **4d** followed with a half-life of about 2 h, while the hexacyclic **4f** was fully bleached within 1 h. The symmetric difuran **4i** fits the same trend, degrading at a rate intermediate between **4h** and **4d**. For comparison, the benchmark acene TIPS-pentacene in identical conditions completely decomposed within 10 min, highlighting the superior resilience of our oxygen-doped analogues (Figure S11).^39^ The inverse correlation between photostability and HOMO–LUMO gaps suggests that lower-gap and more extended systems activates photooxidative pathways, whether through [4 + 4] dimerization or singlet-oxygen attack, whereas the wider-gap members are kinetically protected. We hope that these findings overturn the conventional perception of furan-containing polycycles as inherently fragile and open the door to their deployment in durable organic electronic devices.

## Summary

In summary, we have introduced a catalyst-free, one-pot oxygen-annulation strategy that transforms readily available chloroquinones and aromatic alcohols into a diverse family of π-expanded furan frameworks. The method tolerates both electron-rich and electron-poor substituents and affords unsymmetrical and symmetrical π-expanded, bench-stable architectures in high yields. Single-crystal X-ray diffraction of the heptacyclic derivative **4i** confirms a tightly slipped π-stacked (3.5 Å) packing, foreshadowing efficient solid-state charge transport. Systematic photophysical studies reveal size-dependent, bathochromically tunable absorption (395–486 nm) and strong fluorescence (Φ_PL_ up to 66%), while TD-DFT analysis and pronounced solvatochromism reveal a switch from charge-transfer-dominated transitions in the quinone precursors to localized π–π* excitations in the reduced aromatized furans. Notably, despite long-standing concerns over the photoinstability of furan-containing systems, the presented π-expanded furans outperform TIPS-pentacene, often by more than an order of magnitude, in photodegradation stress tests. We anticipate that oxygen-doped, π-expanded furans will find broad utility as synthetically accessible, photostable, and optoelectronically versatile building blocks for next-generation organic semiconductors, bridging a long-standing gap in the development of heteroatom-rich acene analogues. Our ongoing efforts are directed toward applying this methodology to the construction of larger, three-dimensional π-systems.

## Supplementary Material





## Data Availability

The data underlying this study are available in the published article and its Supporting Information.

## Notes

Cartesian coordinates of all theoretically studied molecules in *xyz* format (ZIP)
General experimental methods, synthetic protocols, electronic absorption and fluorescence spectra, stability experiments, details of the X-ray crystallographic analysis, descriptions of quantum chemical calculations, theoretical results on optoelectronic properties, NICS analysis of aromatic ring currents, and ^1^H and ^13^C NMR spectra (PDF)

## †

Centre of New Technologies, University of Warsaw, S. Banacha 2c, 02–097 Warsaw, Poland. Email: p.gawel@cent.uw.edu.pl
