# Supplementary material for: Photostable π-Expanded Furans via Base-Mediated Quinone Oxygen-Annulation
Source: J Org Chem. 2026 Feb 10;91(7):2837–43. doi: 10.1021/acs.joc.5c02906 (PMC13362199; doi:10.1021/acs.joc.5c02906)
Supplement: Supplementary file 2 [file jo5c02906_si_002.pdf]

Supporting Information

Photostable π-Expanded Furans *via* Base-Mediated Quinone Oxygen-Annulation

Abhishek Pareek,[a] Maja Morawiak,[a] Emran Masoumifeshani,[a] Przemysław Gaweł*[a]

[a]Institute of Organic Chemistry, Polish Academy of Sciences, Kasprzaka 44/52, 01-224 Warsaw, Poland

Contents

S 1. Materials and General Methods S2
S 2. Synthetic Protocols S4
S 3. Proposed Mechanism and Control Experiments S19
S 4. Photophysical Measurements S20
S 5. Stability Studies S26
S 6. Solvatochromism S29
S 7. X-Ray Crystallographic Analysis S32
S 8. Quantum Chemical Calculations S34
S 9. Calculations of Nucleus Independent Chemical Shift (NICS) Plots S39
S 10. ^{1}H and $^{13}C\{^{1}H\}$ NMR Spectra S53
S 11. References S88

S 1. Materials and General Methods

Reagents (Acros, Aldrich, ABCR, and TCI) were purchased as reagent grade and used without further purification.

Solvents for extraction or column chromatography were used analytical grade.

Dry solvents (THF, CH_2Cl_2, diethyl ether, and toluene) for reactions were purified by a solvent drying system from MBraun under nitrogen atmosphere (H_2O content < 10 ppm as determined by Karl-Fischer titration). All other solvents were purchased in p.a. quality.

Reactions in the absence of air and moisture were performed in oven-dried glassware under Ar atmosphere.

Flash column chromatography (FC) was performed using Biotage® Selekt apparatus at 25 °C with a head pressure of 0.0–30 bar and Flow Rate (50-250 mL/min). SiO_2 (60 Å, 230-400 mesh, particle size 0.040-0.063 mm, Fluka). The used solvent compositions are reported in synthetic procedures.

Analytical thin layer chromatography (TLC) was performed on aluminium sheets coated with silica gel 60 F254 (Merck, Macherey-Nagel). Visualization was achieved using UV light (254 or 365 nm), aqueous $KMnO_4$, ceric ammonium molybdate (CAM) solution, or iodine adsorbed on SiO_2.

Evaporation in vacuo was performed at 25–60 °C and 800–10 mbar.

Reported yields refer to spectroscopically and chromatographically pure compounds that were dried under high vacuum (0.5–0.1 mbar) before analytical characterization.

^{1}H and ^{13}C nuclear magnetic resonance (NMR) spectra were recorded on Bruker 400 (Avance III HD), Bruker DRX 500, Varian-Agilent 500 and Varian-Agilent 600 spectrometers at 400 MHz, 500 MHz or 600 MHz (^{1}H) and 75 MHz, 126 MHz or 150 MHz (^{13}C), respectively. Temperatures of measurements are indicated in the procedures and on the spectra. Chemical shifts δ are reported in ppm downfield from tetramethylsilane using the residual deuterated solvent signals as an internal reference ($CDCl_3$: δH = 7.26 ppm, δC = 77.0 ppm). For ^{1}H NMR, coupling constants *J* are given in Hz and the resonance multiplicity is described as s (singlet), d (doublet), t (triplet), q (quartet), m (multiplet). All spectra were recorded at 298 K.

High-resolution mass spectrometry (HR-MS) was performed by the Laboratory for Analysis of Bioactive Compounds at the Institute of Organic Chemistry PAS on a AutoSpec Premier spectrometer (EI), on a 4000 Q-TRAP spectrometer (ESI) and (ACPI), or on a Maldi SYNAPT G2-S HDMS (Waters) spectrometer (MALDI). For MALDI measurements, the matrix was Dithranol or trans-2-[3-(4-tert-Butylphenyl)-2-methyl-2-propenylidene] malononitrile (DCTB).

Melting Points All melting points for crystalline products were measured with automated melting point apparatus EZ-MELT and are reported without correction.

Photophysical measurements in solution were performed at 298 K and conducted using a 10-mm square cell. UV–visible absorption spectra were recorded on a UV-visible spectrophotometer Shimadzu UV-1900i. The steady-state emission spectra and excitation spectra were measured with a spectrofluorometer (Edinburgh FL 980). Photoluminescence quantum yield (Φ_{Fl}) was determined using a comparative method[1] using 9,10-diphenylanthracene as a reference (Φ_{Fl} = 95%).[2]

General Procedures: All reactions were performed in glassware that had been flame-dried under vacuum or oven-dried overnight. All reaction temperatures are noted as the oil bath temperature, the internal temperature as monitored by a Teflon-coated thermocouple), or as the room temperature (approximately 23 °C). Solvents used for extraction were reagent grade, and chromatography solvents were technical grade. Column chromatography was conducted using 230-400 mesh silica gel.

X-Ray Crystallographic Analysis: X-Ray Crystallographic Analysis was performed on a Bruker X8 APEXII diffractometer using CuKα (λ = 1.54178 Å) radiation. Frames were integrated with the Bruker SAINT[3] package using a narrow-frame algorithm. The structure was solved and refined using the Bruker SHELXTL Software Package.[4] Data were corrected for absorption effects using the face-indexed numerical method (SADABS).[5] The structure was solved by direct methods SHELXS-2014 and refined with full-matrix least-squares calculations on F^2 using SHELX-2014.[6]

Crystallographic data have been deposited at the Cambridge Crystallographic Data Centre, 12 Union Road, 129 Cambridge CB21EZ, UK, and copies can be obtained on request, free of charge, by quoting the publication citation and the deposition number. CCDC number for **4i:** 2434127

S 2. Synthetic Protocols

Compounds 2,3-dichloroanthracene-1,4-dione[7], anthracen-2-ol[8], and naphtho[2,3-b]benzofuran-6,11-dione[9] were prepared according to literature procedures.

Dinaphtho[2,1-b:2',3'-d] furan-8,13-dione (3a):

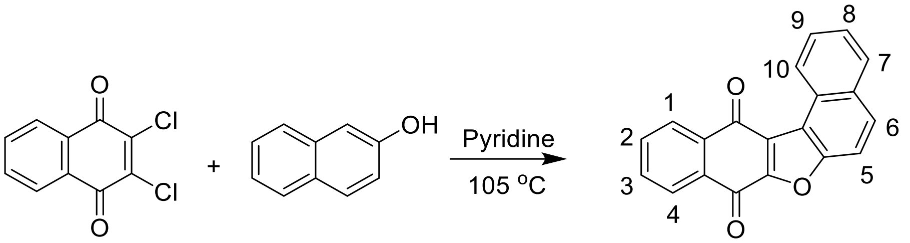

A mixture of 2-naphthol (**2a**) (666.8 mg, 4.62 mmol) and 2,3-dichloro-1,4-naphthoquinone (**1a**) (1.00 g, 4.40 mmol) was dissolved in pyridine (5 mL) and heated at 105 °C with stirring for 24 h. After cooling to room temperature, acetonitrile (5–10 mL) was added dropwise to induce precipitation. The resulting solid was collected by vacuum filtration, washed with acetonitrile (2 × 5 mL), and dried under reduced pressure to afford dinaphtho[2,1-b:2',3'-d]furan-8,13-dione (**3a**) as a light brown solid (1.1 g, 84%).

Rf: 0.50 (Hexanes/EtOAc 7:3)

^{1}H NMR: (500 MHz, $CDCl_3$) δ 9.77 (d, *J* = 8.4 Hz, 1H; H–C (10)), 8.36 (d, *J* = 7.3 Hz, 1H; H–C (4)), 8.29 (d, *J* = 7.2 Hz, 1H; H–C (1)), 8.04 (d, *J* = 9.2 Hz, 1H; H–C (5)), 8.00 (d, *J* = 7.6 Hz, 1H; H–C (7)), 7.84-7.79 (m, 4H; H-C (2, 3, 6, 9)), 7.66 ppm (t, *J* = 8.2 Hz, 1H; H–C (8));

^{13}C{1H} NMR: (126 MHz, $CDCl_3$) δ 181.1, 175.2, 155.7, 153.4, 134.4, 134.0, 133.9, 132.6, 132.2, 131.8, 129.2, 128.6, 128.3, 128.1, 127.8, 126.8, 126.8, 126.7, 119.6, 112.6 ppm.

HRMS: (APCI+) Calcd for $C_{20}H_{11}O_3$ $[M+H]^+$: 299.0708; found: 299.0707

Melting Point: 272 °C

UV/Vis (CH_2Cl_2): λ_{max} (ε) = 446 (6750) 317 (13645) 293 (26285) 284 (26700) 237 (35775 M^{-1} cm^{-1}).

3-Bromodinaphtho[2,1-b:2',3'-d]furan-8,13-dione (3b):

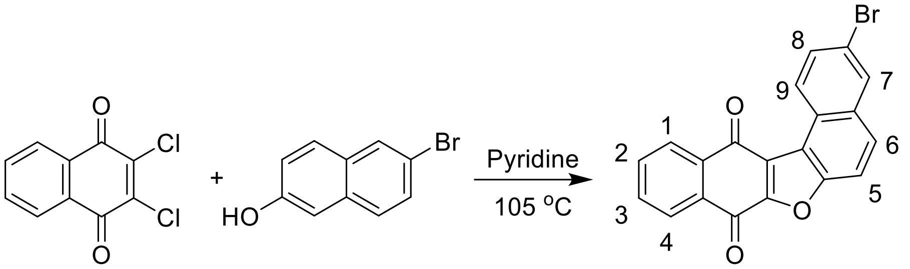

A mixture of 6-bromo-2-naphthol (**2b**) (1.03 g, 4.62 mmol) and 2,3-dichloro-1,4-naphthoquinone (**1a**) (1.00 g, 4.40 mmol) was dissolved in pyridine (5 mL) and heated at 105 °C with stirring for 24 h. After cooling to room temperature, acetonitrile (5–10 mL) was added dropwise to induce precipitation. The resulting solid was collected by vacuum filtration, washed with acetonitrile (2 × 5 mL), and dried under reduced pressure to afford 3-bromodinaphtho[2,1-b:2',3'-d]furan-8,13-dione (**3b**) as a light brown solid (1.3 g, 78%). Owing to its limited solubility in standard organic solvents, this compound was characterized only by HRMS and ^{1}H NMR and employed in the subsequent step without additional purification.

Rf: 0.50 (Hexanes/EtOAc 7:3)

^{1}H NMR: (400 MHz, $CDCl_3$) δ 9.69 (d, *J* = 8.9 Hz, 1H; H–C (9)), 8.81 (br s, 1H; H–C (4)), 8.47 (s, 1H; H–C (1)), 8.36 (d, *J* = 7.0 Hz, 1H; H–C (5)), 8.30 (d, *J* = 7.0 Hz, 1H; H–C (6)), 8.17 (s, 1H; H–C (7)), 7.95 (d, *J* = 8.9 Hz, 1H; H–C (8)), 7.89-7.82 ppm (m, 2H; H–C (2, 3)).

^{13}C{1H} NMR: was not possible to measure due to the low solubility.

HRMS: (APCI+) Calcd for $C_{20}H_{10}O_3Br$ $[M+H]^+$: 376.9813; found: 376.9812

Melting Point: 321 °C

UV/Vis (CH_2Cl_2): λ_{max} (ε) = 438 (8520), 295 (41925), 283 (44545), 254 nm (56570 M^{-1} cm^{-1}).

2-Methoxydinaphtho[2,1-b:2',3'-d]furan-8,13-dione (3c):

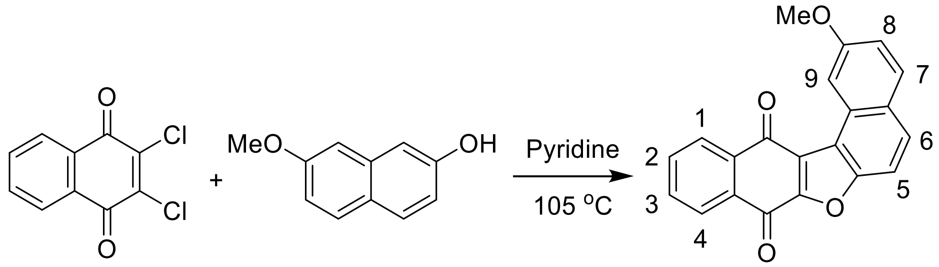

A mixture of 7-methoxy-2-naphthol (**2c**) (322 mg, 1.85 mmol) and 2,3-dichloro-1,4-naphthoquinone (**1a**) (400 mg, 1.76 mmol) was dissolved in pyridine (3 mL) and heated at 105 °C with stirring for 24 h. After cooling to room temperature, acetonitrile (3 mL) was added dropwise to induce precipitation. The resulting solid was collected by vacuum filtration, washed with acetonitrile (2 × 3 mL), and dried under reduced pressure to afford 2-methoxydinaphtho[2,1-b:2',3'-d]furan-8,13-dione (**3c**) as a light brown solid (330 mg, 57%).

Rf: 0.50 (Hexane/EtOAc 6:4)

^{1}H NMR: (500 MHz, $CDCl_3$) δ 9.26 (d, *J* = 2.6 Hz, 1H; H–C (9)), 8.32 (dd, *J* = 6.9, 2.1 Hz, 1H; H–C (4)), 8.26 (dd, *J* = 7.1, 2.0 Hz, 1H; H–C (1)), 7.91 (d, *J* = 8.9 Hz, 1H; H–C (6)), 7.83 (d, *J* = 8.9 Hz, 1H; H–C (7)), 7.81-7.76 (m, 2H; H–C (2, 3)), 7.60 (d, *J* = 8.9 Hz, 1H; H–C (5)), 7.23 (dd, *J* = 8.9, 2.6 Hz, 1H; H–C (8)), 4.15 ppm (s, 3H; OMe);

^{13}C{1H} NMR: (126 MHz, $CDCl_3$) δ 181.2, 175.0, 159.7, 156.2, 153.1, 134.2, 134.0, 133.8, 132.2, 132.1, 130.5, 130.2, 127.7, 126.7, 126.6, 126.6, 119.1, 118.6, 109.8, 107.5, 55.9 ppm.

HRMS: (APCI+) Calcd for $C_{21}H_{13}O_4$ $[M+H]^+$: 329.0816; found: 329.0814

Melting Point: 260 °C

UV/Vis (CH_2Cl_2): λ_{max} (ε) = 466 (1270), 321 (5345), 284 (4230), 247 nm (9910 M^{-1} cm^{-1}).

Dinaphtho[1,2-b:2',3'-d]furan-7,12-dione (3d):

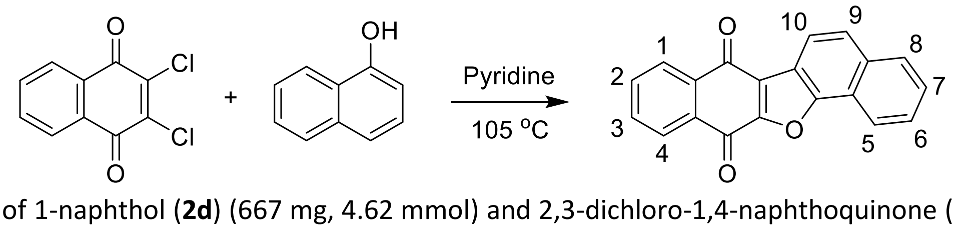

A mixture of 1-naphthol (**2d**) (667 mg, 4.62 mmol) and 2,3-dichloro-1,4-naphthoquinone (**1a**) (1.00 g, 4.40 mmol) was dissolved in pyridine (5 mL) and heated at 105 °C with stirring for 24 h. After cooling

to room temperature, acetonitrile (5 mL) was added dropwise to induce precipitation. The resulting solid was collected by vacuum filtration, washed with acetonitrile (2 × 5 mL), and dried under reduced pressure to afford dinaphtho[1,2-b:2',3'-d]furan-7,12-dione (**3d**) as a light brown solid (1.20 g, 91%).

Rf: 0.50 (Hexanes/EtOAc 7:3)

^{1}H NMR: (600 MHz, $CDCl_3$) δ 8.51 (d, *J* = 8.2 Hz, 1H; H–C (8)), 8.31-8.27 (m, 1H; H–C (2)), 8.27-8.22 (m, 2H; H–C (3, 10)), 7.98 (d, *J* = 8.1 Hz, 1H; H–C (5)), 7.87 (d, *J* = 8.5 Hz, 1H; H–C (4)), 7.82-7.76 (m, 2H; H–C (1, 9)), 7.70 (t, *J* = 6.8 Hz, 1H; H–C (7)), 7.65 ppm (t, *J* = 6.8 Hz, 1H; H–C (6));

^{13}C{1H} NMR: (151 MHz, $CDCl_3$) δ 181.9, 174.9, 153.8, 152.9, 134.2, 134.1, 134.1, 133.5, 132.8, 128.8, 128.1, 127.7, 127.4, 127.1, 127.0, 125.4, 121.3, 121.2, 120.0, 119.3 ppm.

HRMS: (APCI+) Calcd for $C_{20}H_{11}O_3$ $[M+H]^+$: 299.0708; found: 299.0710

Melting Point: 230 °C

UV/Vis (CH_2Cl_2): λ_{max} (ε) = 431 (7530), 340 (3975), 262 nm (89235 M^{-1} cm^{-1}).

Anthra[2,3-b]naphtho[1,2-d]furan-8,15-dione (3e):

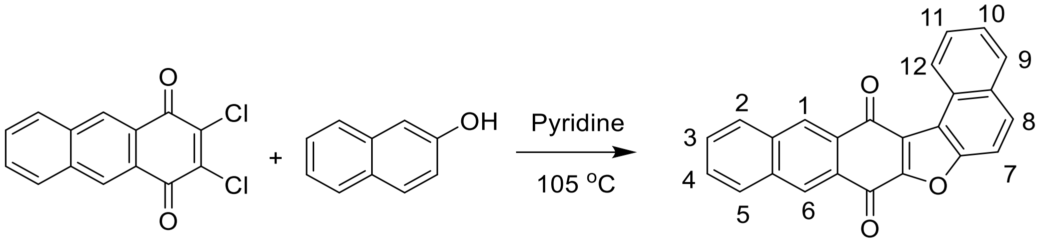

A mixture of 2-naphthol (**2a**) (109 mg, 0.76 mmol) and 2,3-dichloroanthracene-1,4-dione (**1b**) (200 mg, 0.72 mmol) was dissolved in pyridine (3 mL) and heated at 105 °C with stirring for 24 h. After cooling to room temperature, acetonitrile (3 mL) was added dropwise to induce precipitation. The resulting solid was collected by vacuum filtration, washed with acetonitrile (2 × 3 mL), and dried under reduced pressure to afford anthra[2,3-b]naphtho[1,2-d]furan-8,15-dione (**3e**) as a purple solid (214 mg, 85%). Owing to its limited solubility in standard organic solvents, this compound was characterized only by ^{1}H NMR and HRMS and employed in the subsequent step without additional purification.

Rf: 0.50 (Hexanes/EtOAc 7:3)

^{1}H NMR: (400 MHz, $CDCl_3$) δ 9.87 (d, *J* = 8.4 Hz, 1H; H–C (12)), 8.88 (s, 1H; H–C (6)), 8.82 (s, 1H; H–C (1)), 8.14-8.10 (m, 2H; H–C (2, 5)), 8.06-8.00 (m, 2H; H–C (7, 9)), 7.85-7.81 (m, 2H; H–C (8, 11)), 7.74-7.71 (m, 2H; H–C (3, 4)), 7.69-7.63 ppm (m, 1H; H–C (10)).

^{13}C{1H} NMR: was not possible to measure due to the low solubility.

HRMS: (APCI$^+$) Calcd for $C_{24}H_{13}O_3$ $[M+H]^+$: 349.0865; found: 349.0866

Melting Point: 318 °C

UV/Vis (CH_2Cl_2): λ_{max} (ε) = 394 (14360), 285 nm (44235 M^{-1} cm^{-1}).

Anthra[2,3-b]naphtho[2,1-d]furan-7,14-dione (3f):

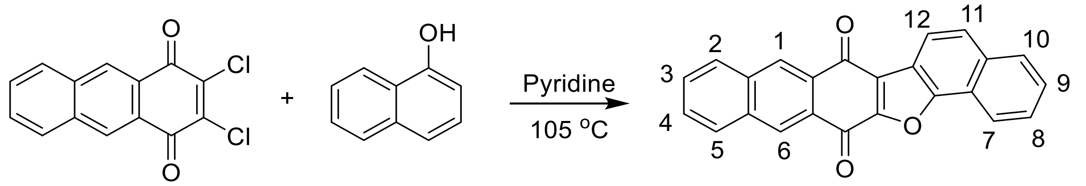

A mixture of 1-naphthol (**2d**) (164 mg, 1.14 mmol) and 2,3-dichloroanthracene-1,4-dione (**1b**) (300 mg, 1.08 mmol) was dissolved in pyridine (3 mL) and heated at 105 °C with stirring for 24 h. After cooling to room temperature, acetonitrile (3 mL) was added dropwise to induce precipitation. The resulting solid was collected by vacuum filtration, washed with acetonitrile (2 × 3 mL), and dried under reduced pressure to afford anthra[2,3-b]naphtho[2,1-d]furan-7,14-dione (**3f**) as a purple solid (320 mg, 85%). Owing to its limited solubility in standard organic solvents, this compound was characterized only by HRMS and ^{1}H NMR and employed in the subsequent step without additional purification.

Rf: 0.50 (Hexanes/EtOAc 7:3)

^{1}H NMR: (400 MHz, $CDCl_3$) δ 8.84 (s, 1H; H–C (6)), 8.78 (s, 1H; H–C (1)), 8.57 (d, *J* = 8.1 Hz, 1H; H–C (10)), 8.35 (d, *J* = 8.6 Hz, 1H; H–C (12)), 8.11 (br s, 2H; H–C (2, 3)), 8.02 (d, *J* = 7.9 Hz, 1H; H–C (5)), 7.91 (d, *J* = 8.7 Hz, 1H; H–C (7)), 7.73-7.66 ppm (m, 4H; H–C (4, 8, 9, 11)).

^{13}C{1H} NMR: was not possible to measure due to the low solubility.

HRMS: (APCI$^+$) Calcd for $C_{24}H_{13}O_3$ $[M+H]^+$: 349.0865; found: 349.0866

Melting Point: 272 °C

UV/Vis (CH_2Cl_2): λ_{max} (ε) = 419 (7885), 300 (27030), 236 nm (36915 M^{-1} cm^{-1}).

Anthra[2,1-b]naphtho[2,3-d]furan-9,14-dione (3g):

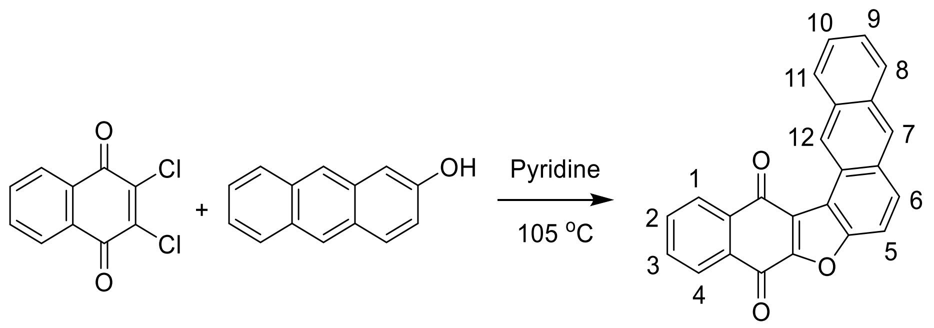

A mixture of anthracen-2-ol (**2e**) (126 mg, 0.65 mmol) and 2,3-dichloro-1,4-naphthoquinone (**1a**) (140 mg, 0.62 mmol) was dissolved in pyridine (3 mL) and heated at 105 °C with stirring for 24 h. After cooling to room temperature, acetonitrile (3 mL) was added dropwise to induce precipitation. The resulting solid was collected by vacuum filtration, washed with acetonitrile (2 × 3 mL), and dried under reduced pressure to afford anthra[2,1-b]naphtho[2,3-d]furan-9,14-dione (**3g**) as a purple solid (151 mg, 70%). Owing to its limited solubility in standard organic solvents, this compound was characterized only by HRMS and ^{1}H NMR and employed in the subsequent step without additional purification.

Rf: 0.50 (Hexanes/EtOAc 7:3)

^{1}H NMR: (400 MHz, $CDCl_3$) δ 10.40 (s, 1H; H–C (12)), 8.57 (s, 1H; H–C (7)), 8.43 (d, *J* = 7.0 Hz, 1H; H–C (4)), 8.35-8.30 (m, 2H; H–C (1, 11)), 8.17 (d, *J* = 8.7 Hz, 1H; H–C (8)), 8.09 (d, *J* = 7.1 Hz, 1H; H–C (5)), 7.84-7.77 (m, 3H; H–C (2, 3, 6)), 7.64-7.60 ppm (m, 2H; H–C (9, 10)).

^{13}C{1H} NMR: was not possible to measure due to the low solubility.

HRMS: (APCI+) Calcd for $C_{24}H_{13}O_3$ $[M+H]^+$: 349.0865; found: 349.0863

Melting Point: 364 °C

UV/Vis (CH_2Cl_2): λ_{max} (ε) = 508 (7095), 388 (8840), 368 (9795), 347 (11865), 284 nm (25780 M^{-1} cm^{-1}).

Dinaphtho[2,1-d;2',1'-d']benzo[1,2-b;4,5-b']difuran-7,15-dione (3i):

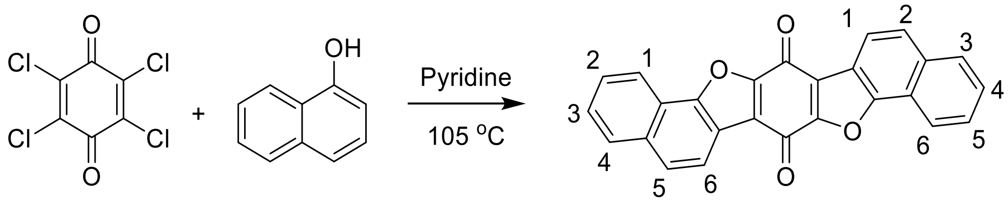

A mixture of 1-naphthol (**2d**) (1.29 g, 8.95 mmol) and tetrachloro-*p*-benzoquinone (**1c**) (1 g, 4.07 mmol) was dissolved in pyridine (5 mL) and heated at 105 °C with stirring for 24 h. After cooling to room temperature, acetonitrile (5 mL) was added dropwise to induce precipitation. The resulting solid was collected by vacuum filtration, washed with acetonitrile (2 × 5 mL), and dried under reduced pressure to afford dinaphtho[2,1-d;2',1'-d']benzo[1,2-b;4,5-b']difuran-7,15-dione (**3i**) as a red crystalline solid (1.15 g, 73%). Due to its limited solubility in common organic solvents, this product was characterized only by HRMS and used directly in the subsequent step without further purification.

Rf: 0.50 (Hexane/EtOAc 6:4)

^{1}H NMR and **^{13}C NMR:** were not possible to measure due to the low solubility.

HRMS: ($APCI^+$) Calcd for $C_{26}H_{13}O_4$ $[M+H]^+$: 349.0814; found: 349.0817

Melting Point: 360 °C

UV/Vis (CH_2Cl_2): λ_{max} (ε) = 443 (2040), 301 (8730), 259 nm (25315 M^{-1} cm^{-1}).

Dinaphtho[1,2-d:1',2'-d']benzo[1,2-b:4,5-b']difuran-8,16-dione (3j):

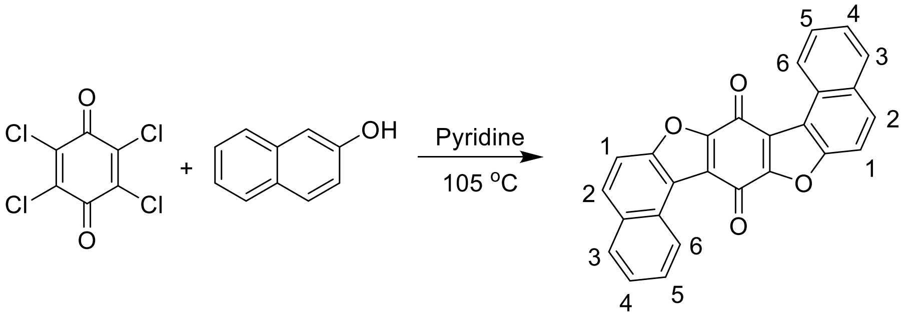

A mixture of 2-naphthol (**2a**) (1.29 g, 8.95 mmol) and tetrachloro-*p*-benzoquinone (**1c**) (1 g, 4.07 mmol) was dissolved in pyridine (5 mL) and heated at 105 °C with stirring for 24 h. After cooling to room temperature, acetonitrile (5 mL) was added dropwise to induce precipitation. The resulting solid was collected by vacuum filtration, washed with acetonitrile (2 × 5 mL), and dried under reduced pressure to afford dinaphtho[1,2-d:1',2'-d']benzo[1,2-b:4,5-b']difuran-8,16-dione (**3j**) as a dark brown crystalline solid (500 mg, 32%). Owing to its limited solubility in standard organic solvents, this compound was characterized only by HRMS and ^{1}H NMR and employed in the subsequent step without additional purification.

Rf: 0.50 (Hexane/EtOAc 6:4)

^{1}H NMR: (400 MHz, $CDCl_3$) δ 9.87 (d, *J* = 8.3 Hz, 2H; H–C (6)), 8.02 (d, *J* = 8.7 Hz, 4H; H–C (1, 3)), 7.90-7.87 (m, 2H; H–C (4)), 7.79 (d, *J* = 8.9 Hz, 2H; H–C (2)), 7.69 ppm (t, *J* = 7.5 Hz, 2H; H–C (5)).

^{13}C{1H} NMR: were not possible to measure due to the low solubility.

HRMS: ($APCI^+$) Calcd for $C_{26}H_{13}O_4$ $[M+H]^+$: 349.0814; found: 349.0815

Melting Point: 390 °C

UV/Vis (CH_2Cl_2): λ_{max} (ε) = 450 (8280), 324 (27795), 300 nm (29270 M^{-1} cm^{-1}).

2,10-Dimethoxy-dinaphtho[1,2-d;1',2'-d']benzo[1,2-b;4,5-b']difuran-8,16-dione (3k):

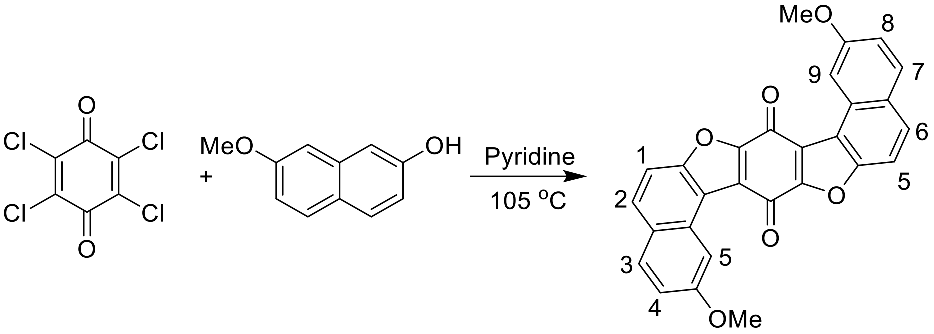

A mixture of 7-methoxy-2-naphthol (**2c**) (623.50 mg, 3.58 mmol) and tetrachloro-*p*-benzoquinone (**1c**) (400 mg, 1.63 mmol) was dissolved in pyridine (5 mL) and heated at 105 °C with stirring for 24 h. After cooling to room temperature, acetonitrile (5 mL) was added dropwise to induce precipitation. The resulting solid was collected by vacuum filtration, washed with acetonitrile (2 × 5 mL), and dried under reduced pressure to afford 2,10-dimethoxy-dinaphtho[1,2-d;1',2'-d']benzo[1,2-b;4,5-b']difuran-8,16-dione (**3k**) as a dark brown solid (386 mg, 53%). Due to its limited solubility in common organic solvents, the product was characterized only by HRMS and used directly in the subsequent step without further purification.

Rf: 0.50 (Hexane/EtOAc 5:5)

^{1}H NMR and **^{13}C NMR:** were not possible to measure due to the low solubility.

HRMS: ($APCI^+$) Calcd. for $C_{28}H_{17}O_6$ $[M+H]^+$: 449.1025; found: 449.1024

Melting Point: 388 °C

UV/Vis (CH_2Cl_2): λ_{max} (ε) = 391 (4520), 322 (6510), 301 (6855), 233 nm (16735 M^{-1} cm^{-1}).

3-(4-(Dimethylamino)phenyl)dinaphtho[2,1-b:2',3'-d]furan-8,13-dione (3l):

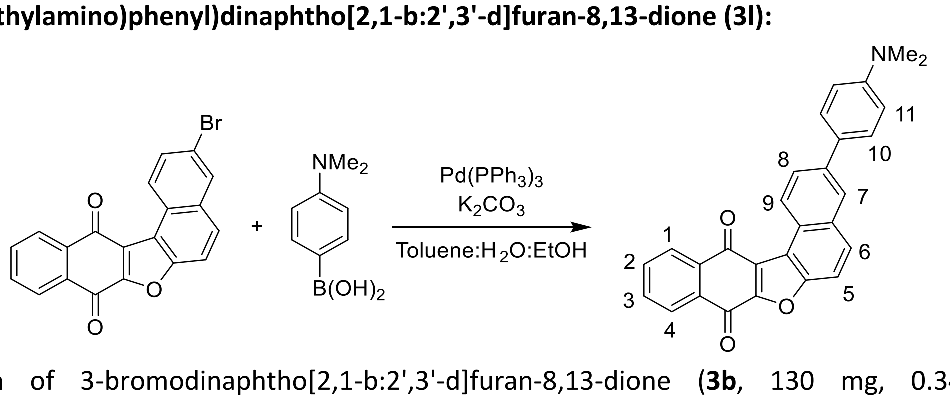

A solution of 3-bromodinaphtho[2,1-b:2',3'-d]furan-8,13-dione (**3b**, 130 mg, 0.34 mmol), (4-(dimethylamino)phenyl) boronic acid (85.3 mg, 0.52 mmol), $Pd(PPh_3)_4$ (15.4 mg, 17.2 µmol), and K_2CO_3 (142.9 mg, 1.03 mmol) in toluene/EtOH/H_2O (5/2/2 mL) mixture was degassed and heated at 100 °C for 12 h under Ar_2. After cooling to room temperature, organic products were extracted with

CH_2Cl_2, and the organic phase was passed through a silica gel plug, eluted with CH_2Cl_2 and evaporated. The residue was purified by flash column chromatography on SiO_2 using toluene as eluent to give **3l** as a reddish solid (110 mg, 76%).

Rf: 0.50 (Hexane/EtOAc 6:4)

^{1}H NMR: (600 MHz, $CDCl_3$) δ 9.71 (d, *J* = 8.7 Hz, 1H; H–C (9)), 8.34 (dd, *J* = 7.3, 1.6 Hz, 1H; H–C (4)), 8.25 (dd, *J* = 7.4, 1.5 Hz, 1H; H–C (1)), 8.08 (d, *J* = 2.1 Hz, 1H; H–C (7)), 8.02 (d, *J* = 8.9 Hz, 1H; H–C (5)), 7.99 (dd, *J* = 8.7, 2.1 Hz, 1H; H–C (8)), 7.86-7.78 (m, 2H; H–C (2, 3)), 7.76 (d, *J* = 8.9 Hz, 1H; H–C (6)), 7.68 (d, *J* = 8.7 Hz, 2H; H–C (10)), 6.89 (br s, 2H; H–C (11)), 3.04 ppm (s, 6H; NMe_2);

^{13}C{1H} NMR: (126 MHz, $CDCl_3$) δ 181.1, 175.1, 155.6, 153.4, 150.4, 139.5, 134.3, 133.9, 133.9, 132.7, 132.4, 132.3, 128.4, 128.1, 127.7, 127.2, 126.7, 126.7, 126.7, 125.5, 119.6, 113.0, 112.7, 40.7 ppm.

HRMS: ($APCI^+$) Calcd for $C_{28}H_{20}NO_3$ $[M+H]^+$: 418.1443; found: 418.1444

Melting Point: 318 °C

UV/Vis (CH_2Cl_2): λ_{max} (ε) = 478 (6355) 312 (55950) 237 (41440 M^{-1} cm^{-1}).

(Dinaphtho[2,1-b:2',3'-d]furan-8,13-diylbis(ethyne-2,1-diyl))bis(triisopropylsilyl) (4a):

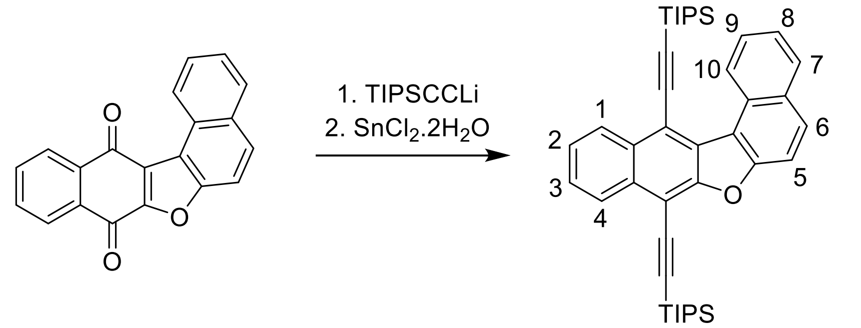

n-BuLi (2.82 mL, 7.05 mmol, 2.5 M in hexanes) was added dropwise to a solution of (triisopropylsilyl)acetylene (1.58 mL, 7.04 mmol) in dry THF (5 mL) at –78 °C and stirred for 30 minutes, followed by the addition of dinaphtho[2,1-b:2',3'-d]furan-8,13-dione (**3a**, 300 mg, 1.01 mmol) in one portion. The reaction mixture was allowed to warm to room temperature and stirred for 16 hours. A solution of $SnCl_2.2H_2O$ (6.03 mmol, 1.36 g) in 5 mL of 10% aqueous HCl was then added and stirring continued for an additional hour. The reaction mixture was extracted with CH_2Cl_2 (25 mL), washed with water (3 × 15 mL), dried over $MgSO_4$, and purified by flash column chromatography (SiO_2, hexanes/CH_2Cl_2, 20:1), affording **4a** as a yellow solid (58%, 365 mg).

Rf: 0.90 (Hexanes/EtOAc 9.5:0.5)

^{1}H NMR: (600 MHz, $CDCl_3$) δ 10.39 (d, *J* = 8.5 Hz, 1H; H–C (10)), 8.89 (d, *J* = 8.5 Hz, 1H; H–C (4)), 8.55 (d, *J* = 7.5 Hz, 1H; H–C (1)), 8.05 (d, *J* = 8.7 Hz, 1H; H–C (5)), 8.01 (d, *J* = 7.3 Hz, 1H; H–C (7)), 7.79 (d, *J* = 8.7 Hz, 1H; H–C (6)), 7.70-7.67 (m, 2H; H–C (2, 9)), 7.65-7.63 (m, 1H; H–C (3)), 7.57-7.54 (m, 1H; H–C (8)), 1.44-1.28 ppm (m, 42H, TIPS);

^{13}C{1H} NMR: (151 MHz, $CDCl_3$) δ 156.8, 156.3, 132.4, 132.0, 131.9, 131.1, 129.7, 129.4, 128.1, 127.8, 127.4, 127.0, 126.7, 126.1, 125.8, 124.6, 117.8, 114.1, 112.8, 107.6, 106.7, 104.1, 99.4, 19.0, 19.0, 11.9, 11.6 ppm.

HRMS: (APCI+) Calcd for $C_{42}H_{53}OSi_2$ $[M+H]^+$: 629.3635; found: 629.3630

Melting Point: 130 °C

UV/Vis (CH_2Cl_2): λ_{max} (ε) = 416 (48940), 393 (32 995), 381 (26665), 332 (23115), 320 (22780), 305 (27045), 293 nm (21750 M^{-1} cm^{-1}).

((3-Bromodinaphtho[2,1-b:2',3'-d]furan-8,13-diyl)bis(ethyne-2,1-diyl))bis(triisopropylsilyl) (4b):

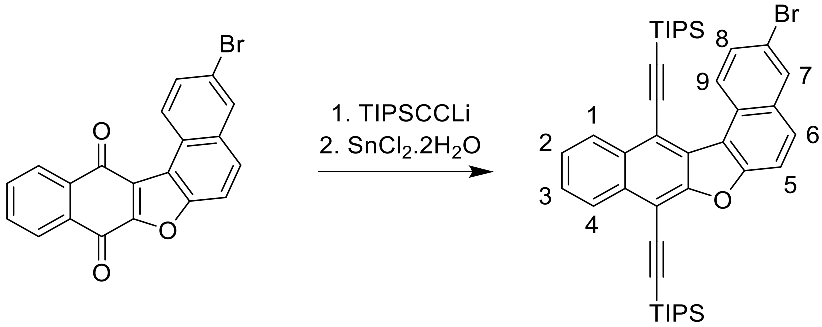

n-BuLi (3.71 mL, 9.28 mmol, 2.5 M in hexanes) was added dropwise to a solution of (triisopropylsilyl)acetylene (2.08 mL, 7.95 mmol) in dry THF (5 mL) at –78 °C and stirred for 30 minutes, followed by the addition of 3-bromodinaphtho[2,1-b:2',3'-d]furan-8,13-dione (**3b**, 500 mg, 1.33 mmol) in one portion. The reaction mixture was allowed to warm to room temperature and stirred for 16 hours. A solution of $SnCl_2·2H_2O$ (7.95 mmol, 1.79 g) in 5 mL of 10% aqueous HCl was then added and stirring continued for an additional hour. The reaction mixture was extracted with CH_2Cl_2 (25 mL), washed with water (3 × 15 mL), dried over $MgSO_4$, and purified by flash column chromatography (SiO_2, hexanes/CH_2Cl_2, 20:1), affording **4b** as a yellow solid (80%, 750 mg).

Rf: 0.90 (Hexanes/EtOAc 9.5:0.5)

^{1}H NMR: (500 MHz, $CDCl_3$) δ 10.28 (d, *J* = 9.0 Hz, 1H; H–C (9)), 8.86 (d, *J* = 8.3 Hz, 1H; H–C (4)), 8.54 (dd, *J* = 8.9, 1.3 Hz, 1H; H–C (1)), 8.14 (d, *J* = 2.2 Hz, 1H; H–C (7)), 7.94 (d, *J* = 8.9 Hz, 1H; H–C (5)), 7.81 (d, *J* = 8.8 Hz, 1H; H–C (6)), 7.73-7.67 (m, 2H; H–C (2, 8)), 7.64 (ddd, *J* = 8.3, 6.7, 1.3 Hz, 1H; H–C (3)), 1.41-1.27 ppm (m, 42H; TIPS);

^{13}C{1H} NMR: (126 MHz, $CDCl_3$) δ 156.7, 156.2, 132.5, 132.4, 132.1, 131.3, 130.7, 129.9, 129.8, 128.2, 127.8, 127.2, 126.8, 126.1, 125.9, 118.4, 118.1, 114.2, 113.9, 108.0, 106.6, 104.5, 104.4, 99.2, 19.0, 19.0, 11.9, 11.6 ppm.

HRMS: (APCI$^+$) Calcd for $C_{42}H_{52}OSi_2Br$ $[M+H]^+$: 707.2740; found: 707.2747

Melting Point: 132 °C

UV/Vis (CH_2Cl_2): λ_{max} (ε) = 418 (44960), 395 (28555), 376 (27500), 337 (21230), 303 (23450), 283 (50695), 272 nm (64050 M^{-1} cm^{-1}).

((2-Methoxydinaphtho[2,1-b:2',3'-d]furan-8,13-diyl)bis(ethyne-2,1-diyl))bis(triisopropylsilyl) (4c):

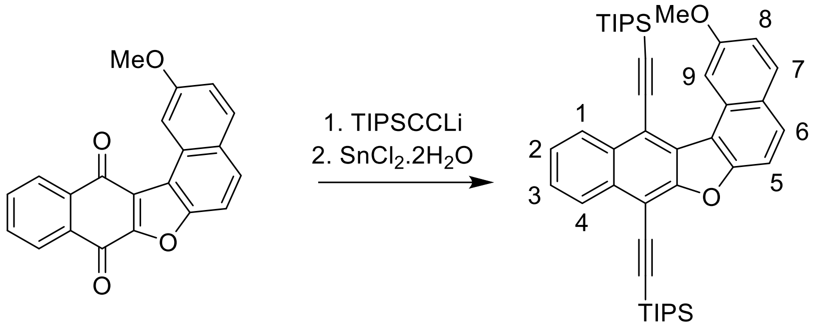

n-BuLi (2.56 mL, 6.40 mmol, 2.5 M in hexanes) was added dropwise to a solution of (triisopropylsilyl)acetylene (1.43 mL, 6.40 mmol) in dry THF (5 mL) at –78 °C and stirred for 30 minutes, followed by the addition of 2-methoxydinaphtho[2,1-b:2',3'-d]furan-8,13-dione (**3c**, 300 mg, 0.90 mmol) in one portion. The reaction mixture was allowed to warm to room temperature and stirred for 16 hours. A solution of $SnCl_2·2H_2O$ (6.03 mmol, 1.36 g) in 5 mL of 10% aqueous HCl was then added and stirring continued for an additional hour. The reaction mixture was extracted with CH_2Cl_2 (25 mL), washed with water (3 × 15 mL), dried over $MgSO_4$, and purified by flash column chromatography (SiO_2, hexanes/CH_2Cl_2, 20:1), affording **4c** as a yellow solid (58%, 347 mg).
Rf: 0.70 (Hexane/EtOAc 9:1)

^{1}H NMR: (600 MHz, $CDCl_3$) δ 9.52 (d, *J* = 2.4 Hz, 1H; H–C (9)), 8.87 (dd, *J* = 8.6, 1.3 Hz, 1H; H–C (4)), 8.53 (dd, *J* = 8.2, 0.8 Hz, 1H; H–C (1)), 7.97 (d, *J* = 8.7 Hz, 1H; H–C (5)), 7.95 (d, *J* = 9.0 Hz, 1H; H–C (7)), 7.68-7.65 (m, 1H; H–C (2)), 7.64-7.61 (m, 2H; H–C (3, 6)), 7.26 (dd, *J* = 8.9, 2.4 Hz, 1H; H–C (8)), 3.99 (s, 3H; OMe), 1.43-1.28 (m, 21H; TIPS), 1.23-1.21 ppm (m, 21H; TIPS);

^{13}C{1H} NMR: (151 MHz, $CDCl_3$) δ 158.6, 157.5, 156.4, 151.9, 132.5, 131.9, 131.7, 130.9, 130.8, 127.7, 127.3, 126.8, 126.3, 126.0, 125.6, 116.7, 113.9, 112.6, 112.6, 110.2, 108.5, 106.4, 103.9, 103.7, 99.5, 55.8, 19.0, 19.0, 11.7, 11.6 ppm.

HRMS: (APCI$^+$) Calcd for $C_{43}H_{55}O_2Si_2$ $[M+H]^+$: 659.3741; found: 659.3742

Melting Point: 113 °C

UV/Vis (CH_2Cl_2): λ_{max} (ε) = 416 (23590), 394 (28245), 325 (22560), 311 (17580), 273 nm (61635 M^{-1} cm^{-1}).

(Dinaphtho[1,2-b:2',3'-d]furan-7,12-diylbis(ethyne-2,1-diyl))bis(triisopropylsilyl) (4d):

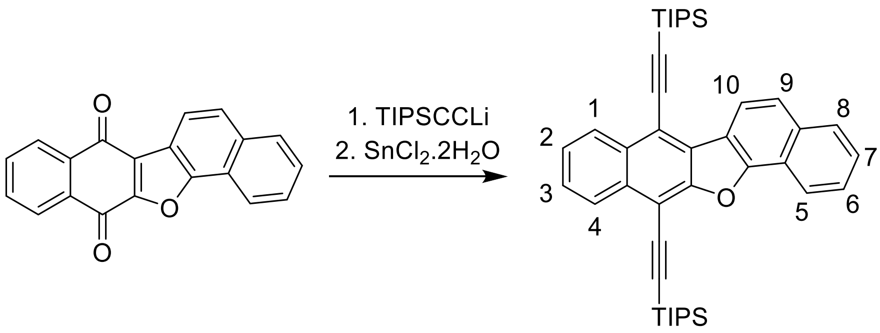

n-BuLi (2.82 mL, 7.04 mmol, 2.5 M in hexanes) was added dropwise to a solution of (triisopropylsilyl)acetylene (1.58 mL, 7.04 mmol) in dry THF (5 mL) at –78 °C and stirred for 30 minutes, followed by the addition of dinaphtho[1,2-b:2',3'-d]furan-7,12-dione (**3d**, 300 mg, 1.01 mmol) in one portion. The reaction mixture was allowed to warm to room temperature and stirred for 16 hours. A

solution of $SnCl_2.2H_2O$ (6.03 mmol, 1.36 g) in 5 mL of 10% aqueous HCl was then added and stirring continued for an additional hour. The reaction mixture was extracted with CH_2Cl_2 (25 mL), washed with water (3 × 15 mL), dried over $MgSO_4$, and purified by flash column chromatography (SiO_2, hexanes/CH_2Cl_2, 20:1), affording **4d** as a yellow solid (54%, 340 mg).

Rf: 0.90 (Hexanes/EtOAc 9.5:0.5)

^{1}H NMR: (600 MHz, $CDCl_3$) δ 8.74 (d, *J* = 8.5 Hz, 1H; H–C (10)), 8.63 (d, *J* = 8.1 Hz, 1H; H–C (4)), 8.55 (d, *J* = 7.2 Hz, 1H; H–C (5)), 8.47 (d, *J* = 8.1 Hz, 1H; H–C (1)), 8.02 (d, *J* = 8.2 Hz, 1H; H–C (8)), 7.83 (d, *J* = 8.5 Hz, 1H; H–C (9)), 7.70-7.65 (m, 2H; H–C (2, 3)), 7.65-7.60 (m, 2H; H–C (6, 7)), 1.39-1.30 ppm (m, 42H; TIPS);

^{13}C{1H} NMR: (151 MHz, $CDCl_3$) δ 156.3, 154.1, 134.1, 132.4, 131.4, 128.5, 127.1, 127.1, 126.8, 126.8, 126.6, 126.5, 125.8, 123.5, 121.5, 121.1, 120.2, 119.1, 113.2, 104.1, 104.0, 103.7, 103.2, 99.4, 19.0, 11.7, 11.6 ppm.

HRMS: (APCI+) Calcd for $C_{42}H_{53}OSi_2$ $[M+H]^+$: 629.3635; found: 629.3641

Melting Point: 206 °C

UV/Vis (CH_2Cl_2): λ_{max} (ε) = 404 (51365), 382 (28720), 365 (22470), 347 (18955), 331 nm (10990 M^{-1} cm^{-1})

(Anthra[2,3-b]naphtho[1,2-d]furan-8,15-diylbis(ethyne-2,1-diyl))bis(triisopropylsilyl) (4e):

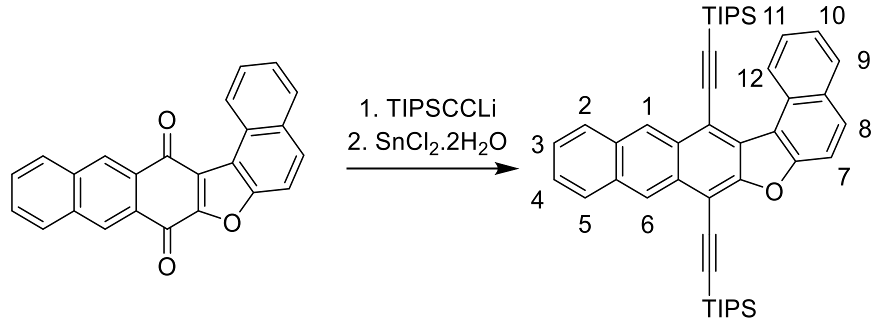

n-BuLi (1.37 mL, 3.42mmol, 2.5 M in hexanes) was added dropwise to a solution of (triisopropylsilyl)acetylene (0.76 mL, 3.42 mmol) in dry THF (3 mL) at –78 °C and stirred for 30 minutes, followed by the addition of anthra[2,3-b]naphtho[1,2-d]furan-8,15-dione (**3e**, 170 mg, 0.48 mmol) in one portion. The reaction mixture was allowed to warm to room temperature and stirred for 16 hours. A solution of $SnCl_2·2H_2O$ (2.93 mmol, 661 mg) in 3 mL of 10% aqueous HCl was then added and stirring continued for an additional hour. The reaction mixture was extracted with CH_2Cl_2 (20 mL), washed with water (3 × 15 mL), dried over $MgSO_4$, and purified by Flash column chromatography on SiO_2, eluent: hexanes/CH_2Cl_2 (≈20:1) with 1% Et_3N, affording **4e** as a yellow solid (62%, 205 mg).

Rf: 0.90 (Hexanes/EtOAc 9.5:0.5)

^{1}H NMR: (500 MHz, $CDCl_3$) δ 10.44 (d, *J* = 8.5 Hz, 1H; H–C (12)), 9.54 (s, 1H; H–C (6)), 9.12 (s, 1H; H–C (1)), 8.09-8.06 (m, 3H; H–C (2, 5, 7)), 8.01 (d, *J* = 6.9 Hz, 1H; H–C (9)), 7.78 (d, *J* = 8.7 Hz, 1H; H–C (8)), 7.72-7.68 (m, 1H; H–C (11)), 7.60-7.47 (m, 3H; H–C (3, 4, 10)), 1.36-1.32 ppm (m, 42H; TIPS);

^{13}C{1H} NMR: (126 MHz, $CDCl_3$) δ 157.4, 156.6, 132.5, 132.1, 131.6, 131.2, 130.6, 129.8, 129.8, 129.4, 128.7, 128.5, 128.1, 128.1, 127.2, 126.9, 126.3, 125.7, 124.7, 124.6, 117.8, 113.9, 112.7, 108.5, 107.1, 104.6, 102.9, 99.8, 19.2, 19.0, 11.9, 11.7 ppm.

HRMS: ($APCI^+$) Calcd for $C_{46}H_{55}OSi_2$ $[M+H]^+$: 679.3791; found: 679.3803

Melting Point: 171 °C

UV/Vis (CH_2Cl_2): λ_{max} (ε) = 486 (22740), 455 (14530), 428 (5940), 406 (18030), 384 (10975), 348 nm (18510 M^{-1} cm^{-1}).

(Anthra[2,3-b]naphtho[2,1-d]furan-7,14-diylbis(ethyne-2,1-diyl))bis(triisopropylsilyl) (4f):

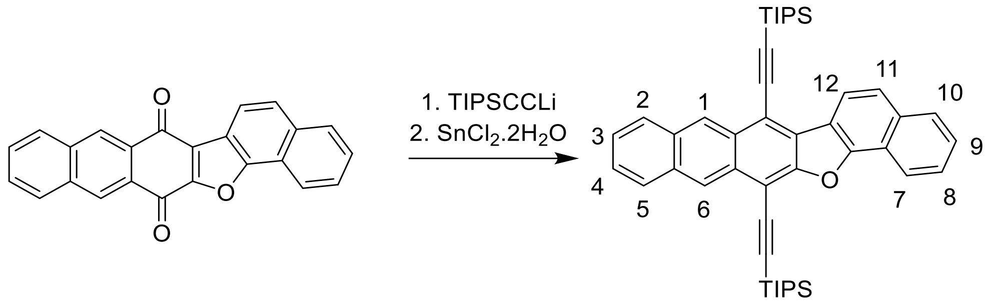

n-BuLi (1.61 mL, 4.02 mmol, 2.5 M in hexanes) was added dropwise to a solution of (triisopropylsilyl)acetylene (0.9 mL, 4.02 mmol) in dry THF (3 mL) at –78 °C and stirred for 30 minutes, followed by the addition of anthra[2,3-b]naphtho[2,1-d]furan-7,14-dione (**3f**, 200 mg, 0.57 mmol) in one portion. The reaction mixture was allowed to warm to room temperature and stirred for 16 hours. A solution of $SnCl_2.2H_2O$ (3.44 mmol, 777 mg) in 3 mL of 10% aqueous HCl was then added and stirring continued for an additional hour. The reaction mixture was extracted with CH_2Cl_2 (20 mL), washed with water (3 × 15 mL), dried over $MgSO_4$, and purified by flash column chromatography (SiO_2, hexanes/CH_2Cl_2, 20:1), affording **4f** as a yellow solid (53%, 207 mg).

Rf: 0.50 (Hexanes/EtOAc 9.5:0.5)

^{1}H NMR: (600 MHz, $CDCl_3$) δ 9.26 (s, 1H; H–C (6)), 9.13 (s, 1H; H–C (1)), 8.76 (d, *J* = 8.4 Hz, 1H; H–C (12)), 8.48 (d, *J* = 8.1 Hz, 1H; H–C (7)), 8.12-8.06 (m, 2H; H–C (2, 5)), 8.02 (d, *J* = 8.1 Hz, 1H; H–C (10)), 7.84 (d, *J* = 8.4 Hz, 1H; H–C (11)), 7.70-7.68 (m, 1H; H–C (8)), 7.64-7.61 (m, 1H; H–C (9)), 7.57-7.52 (m, 2H; H–C (3, 4)), 1.41-1.36 ppm (m, 42H; TIPS);

^{13}C{1H} NMR: (151 MHz, $CDCl_3$) δ 156.6, 154.7, 134.5, 132.0, 131.4, 130.2, 129.7, 128.6, 128.5, 128.3, 127.3, 126.8, 126.2, 125.9, 125.8, 125.2, 123.6, 121.7, 121.0, 120.3, 119.0, 112.9, 105.0, 104.5, 103.5, 102.5, 99.7, 19.1, 19.1, 11.8, 11.7 ppm. (One carbon signal is missing due to the overlap)

HRMS: ($APCI^+$) Calcd for $C_{46}H_{55}OSi_2$ $[M+H]^+$: 679.3791; found: 679.3790

Melting Point: 311 °C

UV/Vis (CH_2Cl_2): λ_{max} (ε) = 474 (38630), 444 (22465), 418 (8305), 395 (22625), 374 (10370), 300 nm (92155 M^{-1} cm^{-1}).

(Anthra[2,1-b]naphtho[2,3-d]furan-9,14-diylbis(ethyne-2,1-diyl))bis(triisopropylsilyl) (4g):

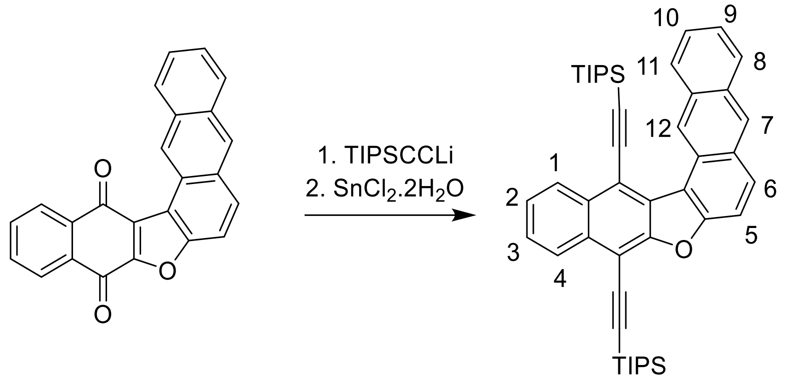

n-BuLi (1.61 mL, 4.02 mmol, 2.5 M in hexanes) was added dropwise to a solution of (triisopropylsilyl)acetylene (0.9 mL, 4.02 mmol) in dry THF (3 mL) at –78 °C and stirred for 30 minutes, followed by the addition of anthra[2,1-b]naphtho[2,3-d]furan-9,14-dione (**3g**, 200 mg, 0.57 mmol) in one portion. The reaction mixture was allowed to warm to room temperature and stirred for 16 hours. A solution of $SnCl_2{\cdot}2H_2O$ (3.44 mmol, 777 mg) in 3 mL of 10% aqueous HCl was then added and stirring continued for an additional hour. The reaction mixture was extracted with CH_2Cl_2 (20 mL), washed with water (3 × 15 mL), dried over $MgSO_4$, and purified by Flash column chromatography on SiO_2, eluent: hexanes/CH_2Cl_2 (≈20:1) with 1% Et_3N, affording **4g** as an orange solid (46%, 180 mg).

Rf: 0.90 (Hexanes/EtOAc 9.5:0.5)

^{1}H NMR: (600 MHz, $CDCl_3$) δ 10.48 (s, 1H; H–C (12)), 8.91 (d, *J* = 8.5 Hz, 1H; H–C (4)), 8.59-8.52 (m, 2H; H–C (1, 7)), 8.22 (d, *J* = 7.9 Hz, 1H; H–C (11)), 8.20 (d, *J* = 9.0 Hz, 1H; H–C (5)), 8.06 (d, *J* = 7.9 Hz, 1H; H–C (8)), 7.78 (d, *J* = 9.0 Hz, 1H; H–C (6)), 7.71-7.63 (m, 2H; H–C (2, 3)), 7.56-7.51 (m, 2H; H–C (9, 10)), 1.41-1.28 (m, 21H; TIPS), 1.19-1.17 ppm (m, 21H; TIPS);

^{13}C{1H} NMR: (151 MHz, $CDCl_3$) δ 156.9, 156.3, 132.8, 132.7, 132.1, 131.8, 130.5, 130.0, 128.7, 128.2, 128.2, 127.7, 127.2, 127.1, 126.8, 126.5, 126.2, 125.8, 125.8, 125.5, 116.6, 113.8, 113.7, 108.5, 106.6, 104.1, 104.0, 99.4, 19.0, 11.9, 11.6 ppm.

HRMS: (APCI$^+$) Calcd for $C_{46}H_{55}OSi_2$ $[M+H]^+$: 679.3791; found: 679.3787

Melting Point: 144 °C

UV/Vis (CH_2Cl_2): λ_{max} (ε) = 455 (19610), 429 (16565), 403 (26165), 384 (22415), 370 (19230), 295 nm (77415 M^{-1} cm^{-1}).

(Naphtho[2,3-b]benzofuran-6,11-diylbis(ethyne-2,1-diyl))bis(triisopropylsilyl) (4h):

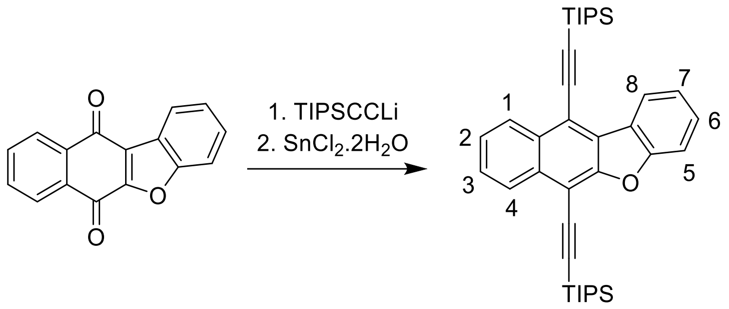

n-BuLi (0.79 mL, 1.97 mmol, 2.5 M in hexanes) was added dropwise to a solution of (triisopropylsilyl)acetylene (0.44 mL, 1.97 mmol) in dry THF (5 mL) at –78 °C and stirred for 30 minutes, followed by the addition of naphtho[2,3-b]benzofuran-6,11-dione (**3h**, 70 mg, 0.28 mmol) in one portion. The reaction mixture was allowed to warm to room temperature and stirred for 16 hours. A solution of $SnCl_2.2H_2O$ (1.69 mmol, 381 mg) in 5 mL of 10% aqueous HCl was then added and stirring continued for an additional hour. The reaction mixture was extracted with CH_2Cl_2 (25 mL), washed with water (3 × 15 mL), dried over $MgSO_4$, and purified by flash column chromatography (SiO_2, hexanes/CH_2Cl_2, 20:1), affording **4h** as a yellow solid (61%, 100 mg).

Rf: 0.90 (Hexanes/EtOAc 9.5:0.5)

^{1}H NMR: (500 MHz, $CDCl_3$) δ 8.69 (d, *J* = 7.7 Hz, 1H; H–C (8)), 8.59 (d, *J* = 8.0 Hz, 1H; H–C (4)), 8.52 (d, *J* = 8.3 Hz, 1H; H–C (1)), 7.68-7.59 (m, 3H; H–C (2, 3, 5)), 7.56 (t, *J* = 8.5 Hz, 1H; H–C (6)), 7.39 (t, *J* = 7.5 Hz, 1H; H–C (7)), 1.37-1.25 ppm (m, 42H; TIPS);

^{13}C{1H} NMR: (126 MHz, $CDCl_3$) δ 157.8, 156.2, 133.0, 131.1, 129.0, 127.1, 126.6, 126.4, 126.3, 125.7, 123.9, 123.4, 123.0, 113.9, 111.8, 104.6, 103.8, 103.3, 102.9, 99.3, 19.0, 19.0, 11.7, 11.6 ppm.

HRMS: (APCI$^+$) Calcd for $C_{38}H_{50}ONaSi_2$ $[M+Na]^+$: 601.3295; found: 601.3298

Melting Point: 133 °C

UV/Vis (CH_2Cl_2): λ_{max} (ε) = 395 (25350) 375 (14710) 356 (12845 M^{-1} cm^{-1}).

7,14-Bis((triisopropylsilyl)ethynyl)dinaphtho[1,2-b:2',1'-d]benzofuran (4i):

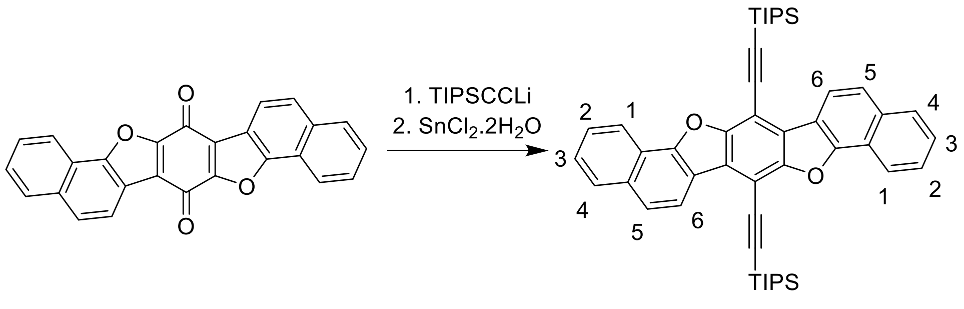

n-BuLi (2.16 mL, 5.41 mmol, 2.5 M in hexanes) was added dropwise to a solution of (triisopropylsilyl)acetylene (1.21 mL, 5.41 mmol) in dry THF (3 mL) at –78 °C and stirred for 30 minutes, followed by the addition of dinaphtho[1,2-d:1',2'-d']benzo[1,2-b:4,5-b']difuran-8,16-dione (**3i**, 300 mg, 0.77 mmol) in one portion. The reaction mixture was allowed to warm to room temperature and stirred for 16 hours. A solution of $SnCl_2·2H_2O$ (4.63 mmol, 1.05 g) in 3 mL of 10% aqueous HCl was then added and stirring continued for an additional hour. The reaction mixture was extracted with CH_2Cl_2 (20 mL),

washed with water (3 × 15 mL), dried over $MgSO_4$, and purified by flash column chromatography on SiO_2, eluent: hexanes/CH_2Cl_2 (≈20:1) with 1% Et_3N, affording **4i** as an orange solid (32%, 120 mg).

Rf: 0.90 (Hexanes/EtOAc 9.5:0.5)

^{1}H NMR: (600 MHz, $CDCl_3$) δ 8.64 (d, *J* = 8.5 Hz, 2H; H–C (6)), 8.47 (d, *J* = 8.1 Hz, 2H; H–C (1)), 8.03 (d, *J* = 8.0 Hz, 2H; H–C (4)), 7.85 (d, *J* = 8.5 Hz, 2H; H–C (5)), 7.69 (t, *J* = 6.9 Hz, 2H; H–C (2)), 7.61 (t, *J* = 6.9 Hz, 2H; H–C (3)), 1.44-1.30 ppm (m, 42H; TIPS);

^{13}C{1H} NMR: (101 MHz, $CDCl_3$): δ 154.0, 153.3, 133.5, 128.6, 126.8, 126.7, 124.0, 123.6, 121.3, 121.2, 119.9, 119.6, 103.4, 100.8, 99.4, 19.0, 11.7 ppm.

HRMS: (APCI$^+$) Calcd for $C_{48}H_{55}O_2Si_2$ $[M+H]^+$: 719.3741; found: 719.3730

Melting Point: 380 °C

UV/Vis (CH_2Cl_2): λ_{max} (ε) = 396 (38725), 375 (38660), 358 (21090), 326 (15540), 287 nm (34435 M^{-1} cm^{-1}).

7,14-Bis((triisopropylsilyl)ethynyl)dinaphtho[2,1-b:1',2'-d]benzofuran (4j):

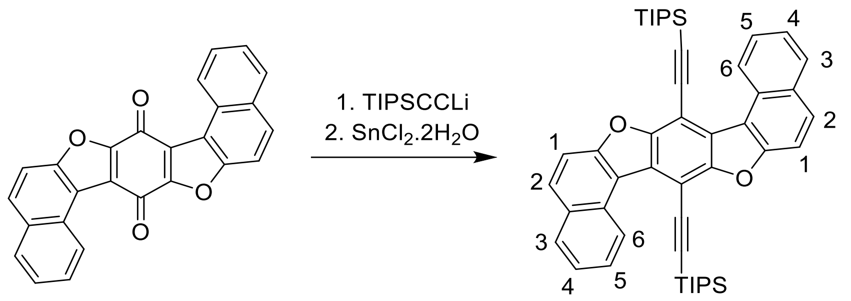

n-BuLi (1.43 mL, 3.59 mmol, 2.5 M in hexanes) was added dropwise to a solution of (triisopropylsilyl)acetylene (0.81 mL, 3.59 mmol) in dry THF (3 mL) at –78 °C and stirred for 30 minutes, followed by the addition of dinaphtho[2,1-b:2',3'-d]furan-8,13-dione (200 mg, 0.51 mmol) in one portion. The reaction mixture was allowed to warm to room temperature and stirred for 16 hours. A solution of $SnCl_2 \cdot 2H_2O$ (3.07 mmol, 697 mg) in 3 mL of 10% aqueous HCl was then added and stirring continued for an additional hour. The reaction mixture was extracted with CH_2Cl_2 (20 mL), washed with water (3 × 10 mL), dried over $MgSO_4$, and purified by flash column chromatography on SiO_2, eluent: hexanes/CH_2Cl_2 (≈20:1) with 1% Et_3N, affording **4j** as an orange solid (24%, 91 mg).

Rf: 0.90 (Hexane/EtOAc 9.5:0.5)

^{1}H NMR: (400 MHz, $CDCl_3$) δ 10.36 (d, *J* = 8.6 Hz, 2H; H–C (6)), 8.04-8.00 (m, 4H; H–C (1, 3)), 7.76 (d, *J* = 8.8 Hz, 2H; H–C (2)), 7.69 (t, *J* = 6.8 Hz, 2H; H–C (4)), 7.57 (t, *J* = 6.8 Hz, 2H; H–C (5)), 1.43-1.34 ppm (m, 42H; TIPS);

^{13}C{1H} NMR: (101 MHz, $CDCl_3$) δ 156.6, 155.9, 131.0, 130.5, 129.5, 129.3, 127.9, 126.8, 124.7, 123.1, 118.7, 112.7, 106.8, 102.6, 101.9, 19.0, 11.8 ppm.

HRMS: (APCI$^+$) Calcd for $C_{48}H_{55}O_2Si_2$ $[M+H]^+$: 719.3662; found: 719.3658

Melting Point: 368 °C

UV/Vis (CH_2Cl_2): λ_{max} (ε) = 411 (44970), 388 (46140), 306 (30220), 269 (48045), 229 nm (35955 M^{-1} cm^{-1}).

2,10-dimethoxy-7,14-Bis((triisopropylsilyl)ethynyl)dinaphtho[2,1-b:1',2'-d]benzofuran (4k):

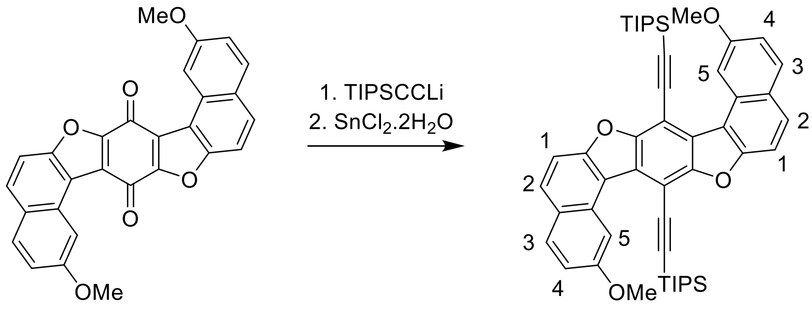

n-BuLi (2.41 mL, 6.03 mmol, 2.5 M in hexanes) was added dropwise to a solution of (triisopropylsilyl)acetylene (1.35 mL, 6.03 mmol) in dry THF (5 mL) at –78 °C and stirred for 30 minutes, followed by the addition of 2-methoxydinaphtho[2,1-b:2',3'-d]furan-8,13-dione (**3k**, 386 mg, 0.86 mmol) in one portion. The reaction mixture was allowed to warm to room temperature and stirred for 16 hours. A solution of $SnCl_2{\cdot}2H_2O$ (5.16 mmol, 1.17 g) in 5 mL of 10% aqueous HCl was then added and stirring continued for an additional hour. The reaction mixture was extracted with CH_2Cl_2 (25 mL), washed with water (3 × 15 mL), dried over $MgSO_4$, and purified by flash column chromatography (SiO_2, hexanes/CH_2Cl_2, 20:1), affording **4k** as an orange solid (39%, 260 mg).

Rf: 0.90 (Hexanes/EtOAc 9:1)

^{1}H NMR: (500 MHz, $CDCl_3$) δ 9.63 (d, *J* = 2.6 Hz, 2H; H–C (5)), 7.97 (d, *J* = 8.9 Hz, 2H; H–C (3)), 7.92 (d, *J* = 8.7 Hz, 2H; H–C (1)), 7.59 (d, *J* = 8.7 Hz, 2H; H–C (2)), 7.27 (dd, *J* = 8.9, 2.6 Hz, 2H; H–C (4)), 4.00 (s, 6H; OMe), 1.47-1.31 ppm (m, 42H; TIPS);

^{13}C{1H} NMR: (126 MHz, $CDCl_3$) δ 158.8, 156.5, 156.4, 130.8, 130.2, 126.1, 122.7, 117.7, 112.6, 112.4, 110.2, 107.4, 102.5, 101.7, 55.9, 19.0, 11.7 ppm.

HRMS: ($APCI^+$) Calcd for $C_{50}H_{59}O_4Si_2$ $[M+H]^+$: 779.3952; found: 779.3946

Melting Point: 321 °C

UV/Vis (CH_2Cl_2): λ_{max} (ε) = 422 (63360), 398 (49275), 381 (66915), 363 (29870), 304 (64900), 272 nm (89420 M^{-1} cm^{-1}).

S 3. Proposed Mechanism and Control Experiments

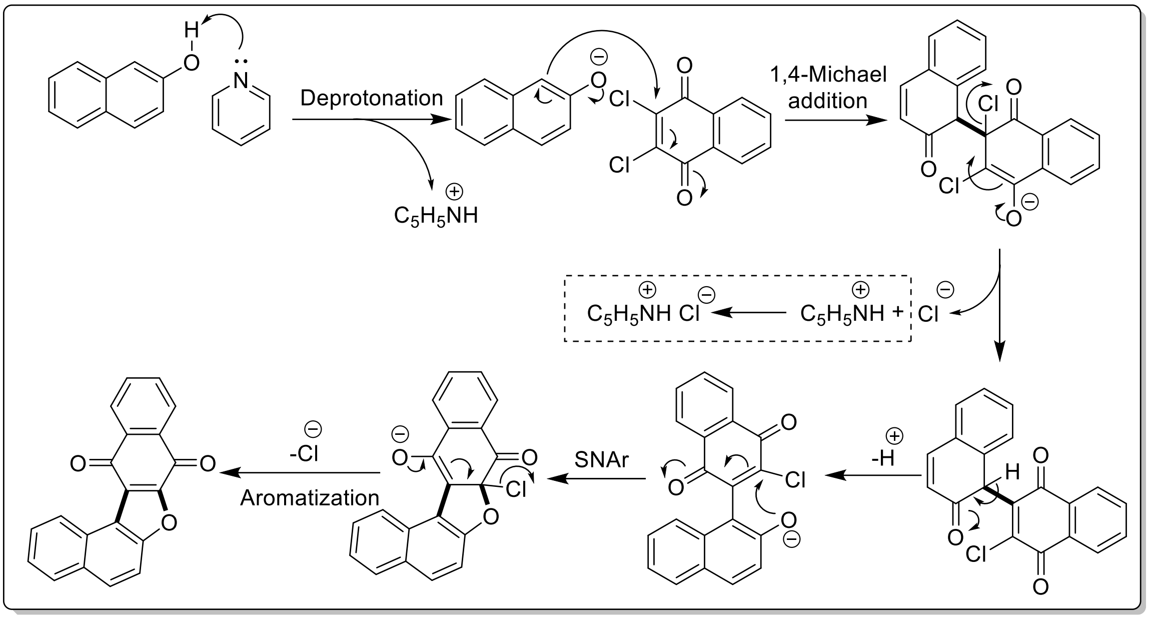

Figure S 1: Plausible mechanism proposed for the synthesis of furan-fused quinones.

Control experiments established that pyridine is essential for the formation of quinone (**3**), serving as both base and solvent. Optimal yields were obtained at 105 °C. Omission of pyridine or substitution with other bases such as Et_3N led to significantly reduced or no product formation, suggesting that pyridine plays a unique role beyond simple proton abstraction, possibly involving stabilization or activation of key intermediates. The free hydroxy group in 2-naphthol is also critical; replacement with a methoxy group completely suppressed reactivity, indicating that nucleophilic attack is fundamental to the transformation. Additionally, lower reaction temperatures or changes in solvent led to diminished yields, underscoring the importance of both thermal input and the specific solvent environment. These findings highlight the necessity of the hydroxy functionality, pyridine as a multifunctional promoter, and elevated temperature for efficient quinone formation. Thus, the combination of a free hydroxy group, pyridine as a dual-role base and solvent, and elevated temperature is mechanistically essential for successful quinone formation.

Table S 1: Control experiments for the synthesis of quinones

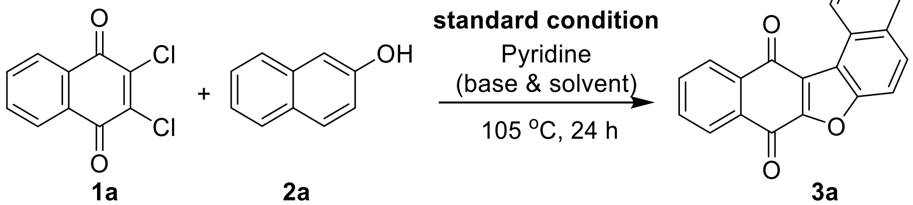

1a **2a** **3a**

Entry	Variation from the standard conditions	Product
1.	No deviation	91%
2.	2-methoxynaphthalene instead of 2-naphthol	No reaction
3.	No Base, DCE, 85 °C, 24 h	No reaction
4.	Pyridine, CH_2Cl_2, 25 °C	50%
5.	Without 2-naphthol	Insoluble solid formed
6.	No Base, toluene, 105 °C, 24 h	No desired product
7.	Et_3N, 90 °C, 24 h	30%

(a). Substrate **1a** (1 equiv), substrate **2a** (1.2 equiv), DCE: 1,2-dichloroethane; CH_2Cl_2: dichloromethane

S 4. Photophysical Measurements

Table S 2. Photophysical and Computational data for **4a**–**k**.

Compounds	UV–vis		Fluorescence				DFT
	λ_{abs} nm	eV	λ_{em} nm	eV	Stokes shift eV	Φ_{PL}	$\Delta E_{TD\ DFT}$
**4a**	416	2.98	420	2.95	0.03	0.16	3.26
**4b**	418	2.97	428	2.90	0.07	0.15	3.26
**4c**	416	2.98	448	2.77	0.21	0.52	3.26
**4d**	404	3.07	414	2.99	0.08	0.48	3.34
**4e**	486	2.55	495	2.51	0.05	0.24	2.79
**4f**	474	2.62	481	2.58	0.04	0.36	2.84
**4g**	455	2.73	493	2.52	0.21	0.66	3.06
**4h**	395	3.14	403	3.07	0.07	0.33	3.43
**4i**	396	3.13	414	3.0	0.13	0.55	3.50
**4j**	411	3.02	428	2.90	0.12	0.48	3.37
**4k**	422	2.94	442	2.81	0.13	0.50	3.30

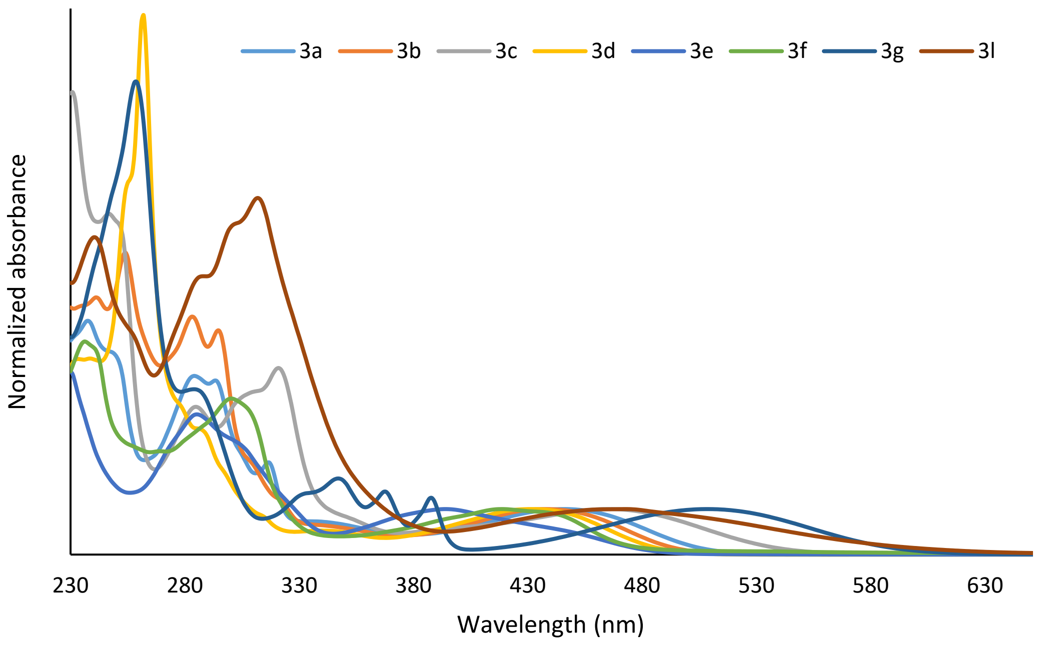

Figure S 2: UV-Vis absorption spectra of unsymmetrical quinones **3a**–**l** normalized at 380-580 nm region.

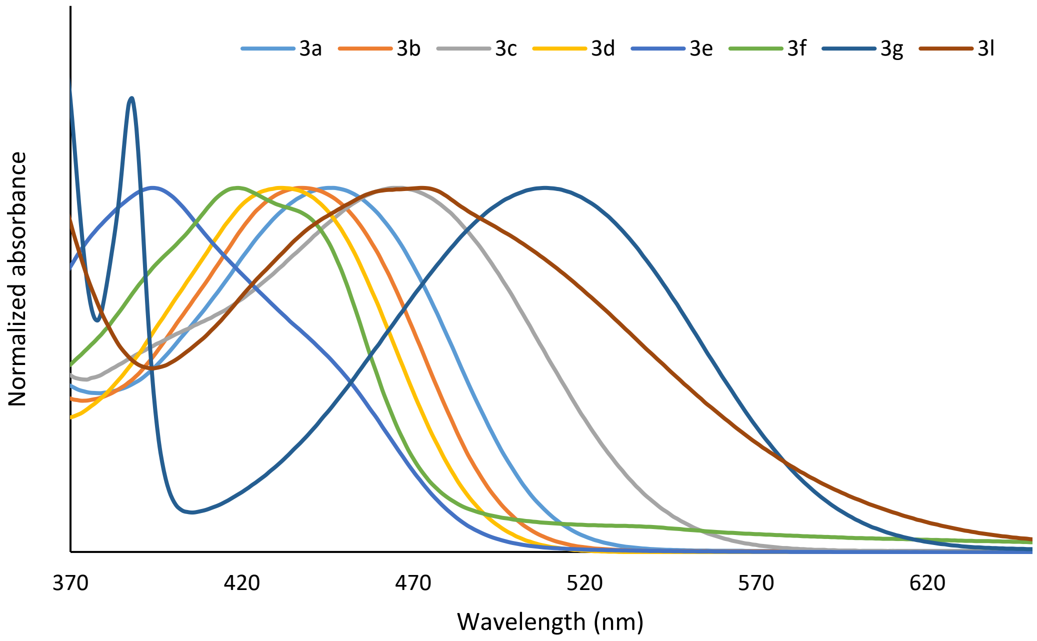

Figure S 3: Normalized UV-Vis absorption spectra of unsymmetrical quinones **3a–l**.

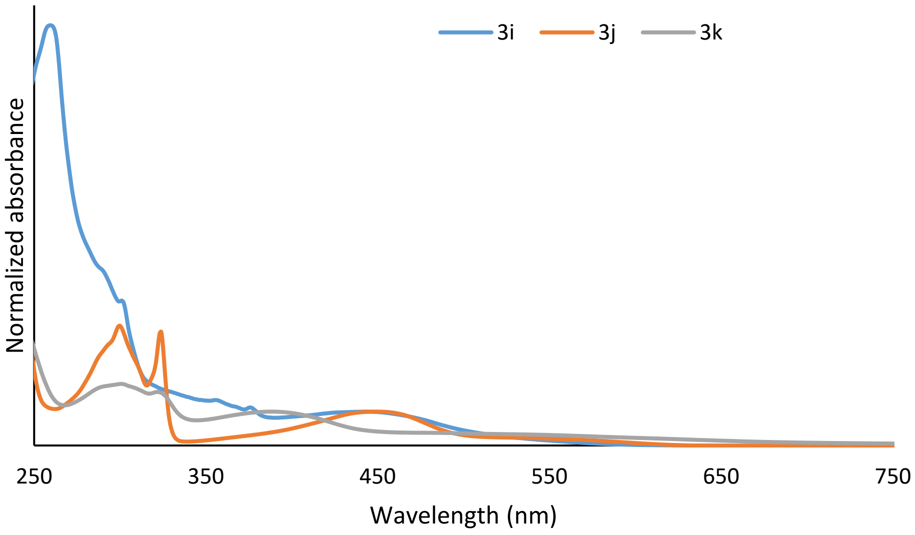

Figure S 4: UV-Vis absorption spectra of symmetrical quinones **3i–k** normalized in the 380–580 nm region.

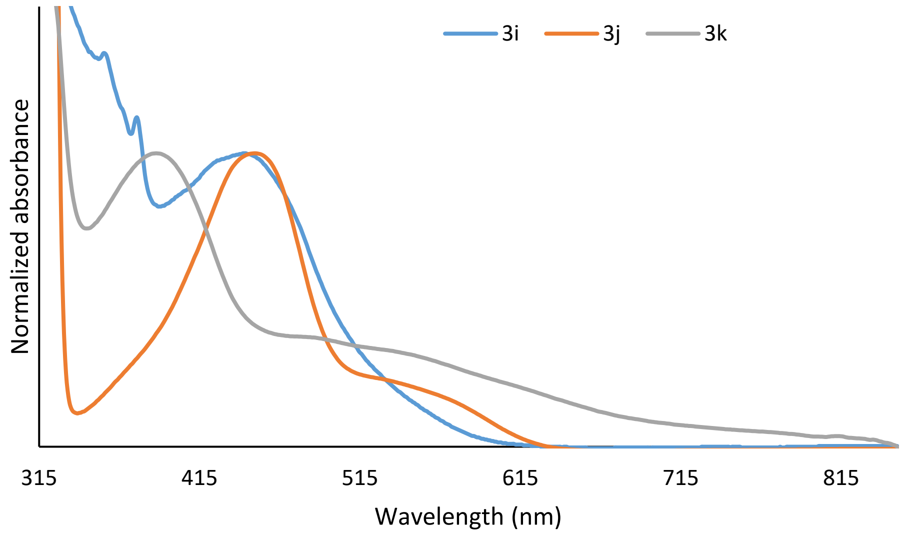

Figure S 5: Normalized UV-Vis absorption spectra of symmetrical quinones **3i**–**k** normalized in the 380–580 nm region.

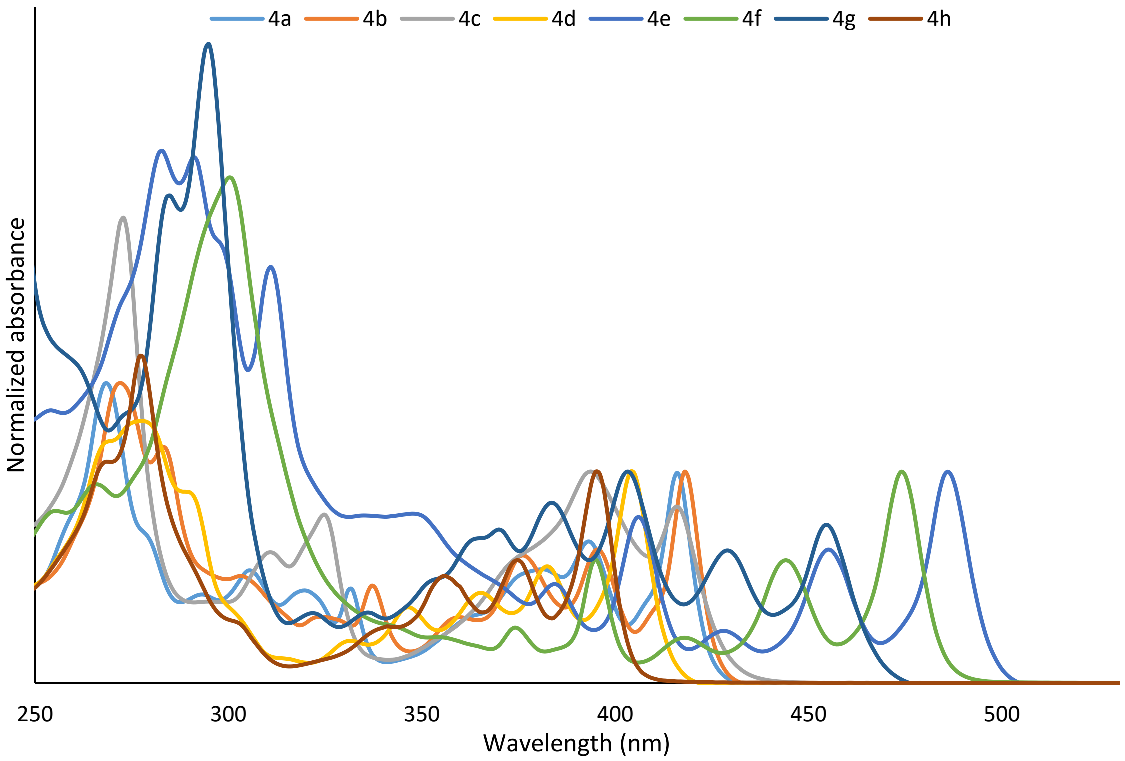

Figure S 6: UV-Vis absorption spectra of unsymmetrical furans **4a**–**h** normalized in the 380–510 nm region.

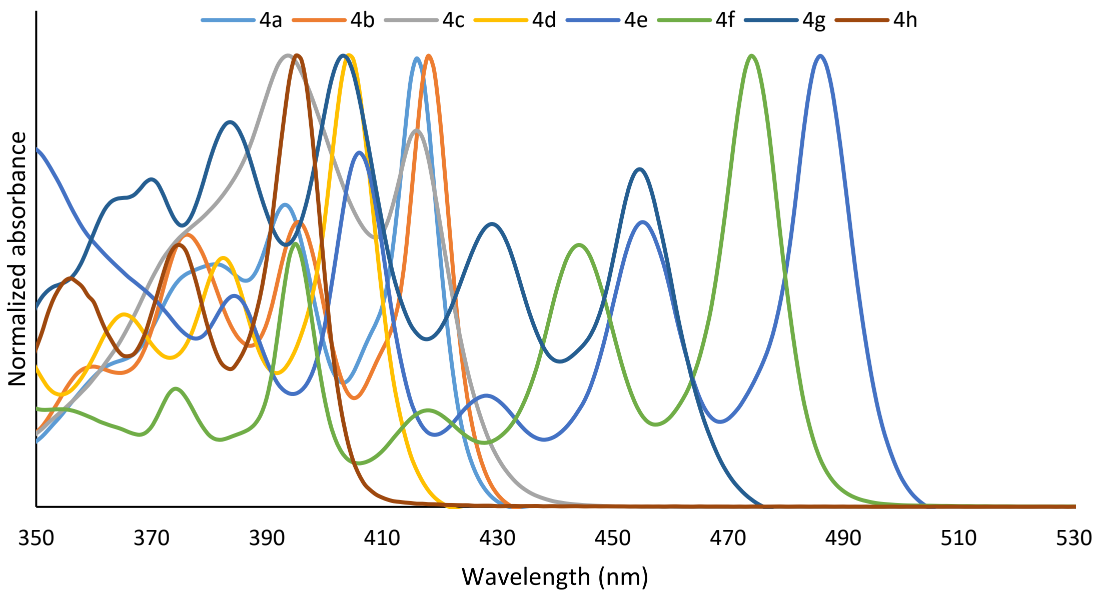

Figure S 7: Normalized UV-Vis absorption spectra of unsymmetrical furans **4a**–**h**.

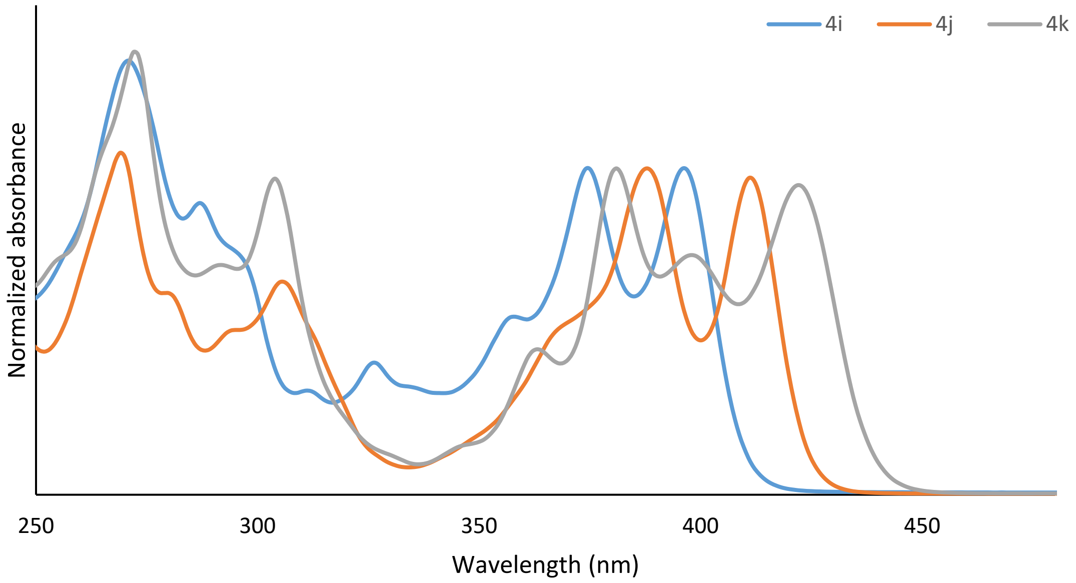

Figure S 8: UV-Vis absorption spectra of symmetrical furans **4i**–**k** normalized in the 350–510 nm region.

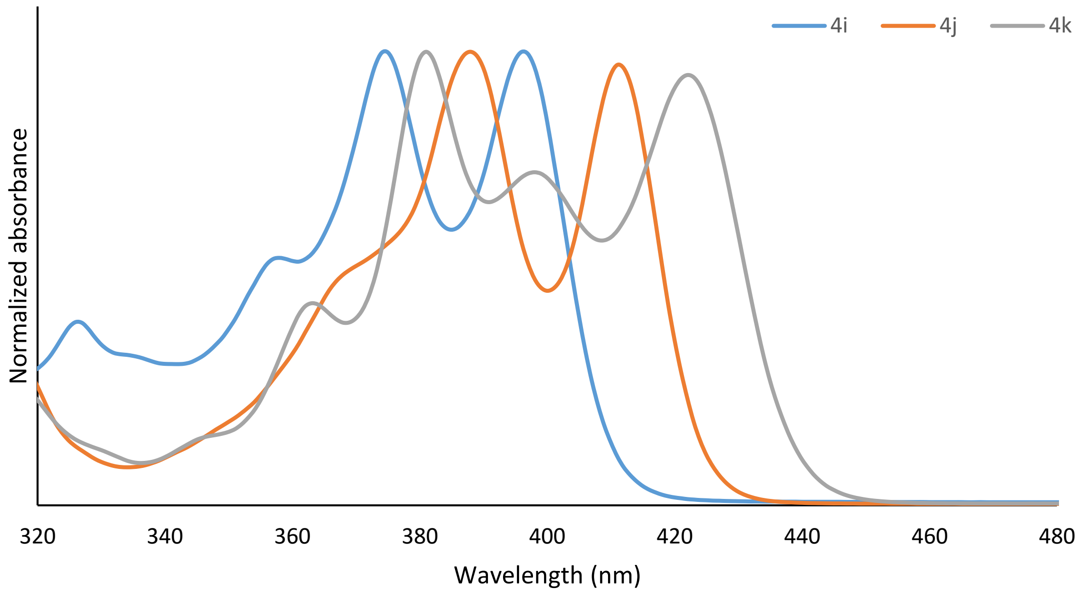

Figure S 9: Normalized UV-Vis absorption spectra of symmetrical furans **4i**–**k**

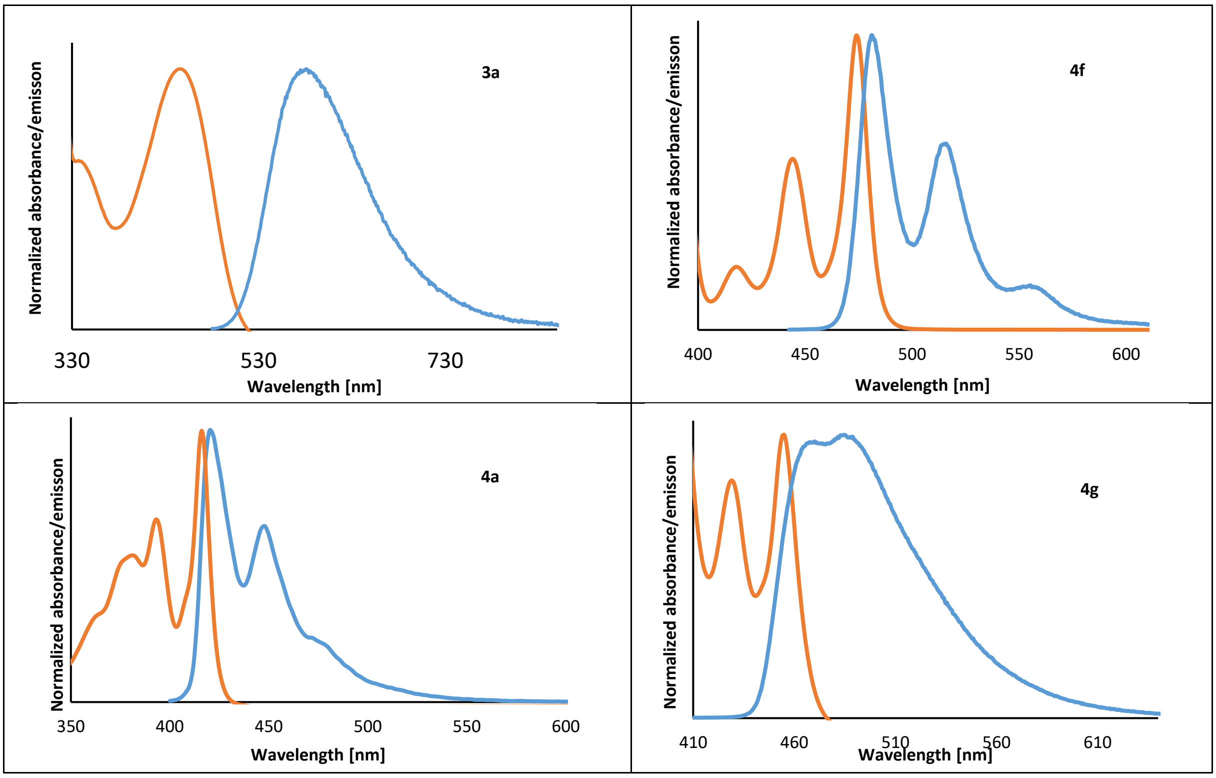

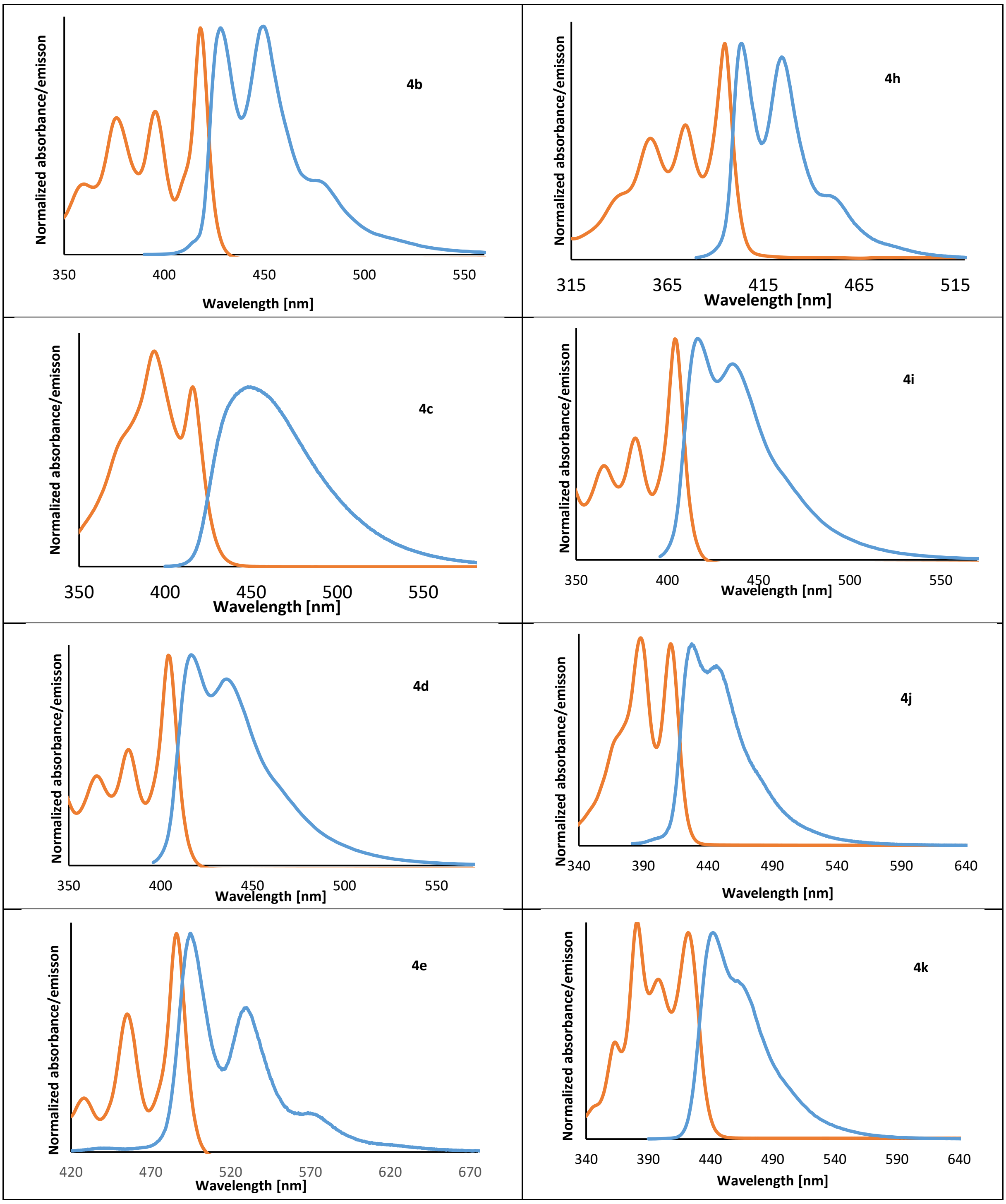

Figure S 10: The normalized absorption and emission spectra of compounds **3a** and **4a–k.**

S 5. Stability Studies

Photostability of the selected furan-fused compounds **4d**, **4f**, **4h**, and **4i**, featuring different numbers of fused aromatic rings was evaluated by irradiating their air-equilibrated CH_2Cl_2 solutions with a 4 W handheld UV lamp (λ = 254 nm) at room temperature. UV-Vis absorption spectra were recorded at selected time intervals (until complete decomposition) to monitor the decay of the parent compounds. (Figures **S11** to **S15**). Photostability profiles were compared by plotting the relative absorbance intensities of selected furan compounds alongside TIPS- pentacene.

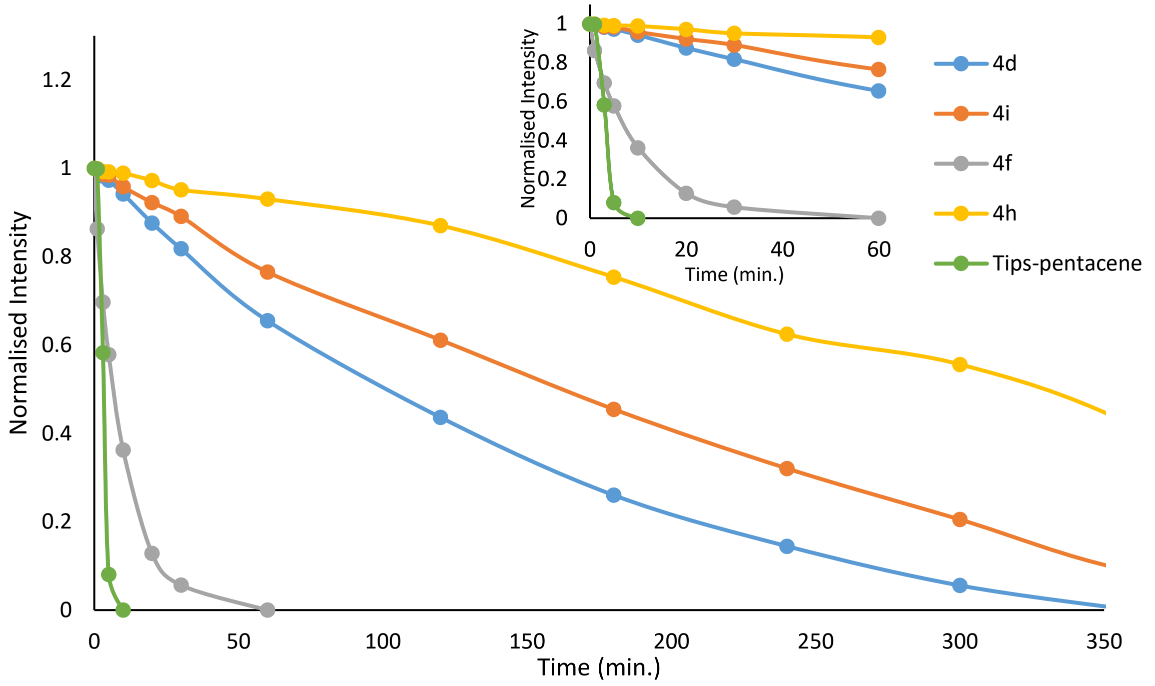

Figure S 11. Relative intensity of λ_{max} upon irradiation with 254 nm light (4W lamp) in aerated CH_2Cl_2 (corrected for absorption of forming new species; TIPS-Pentacene (CAS: 373596-08-8)

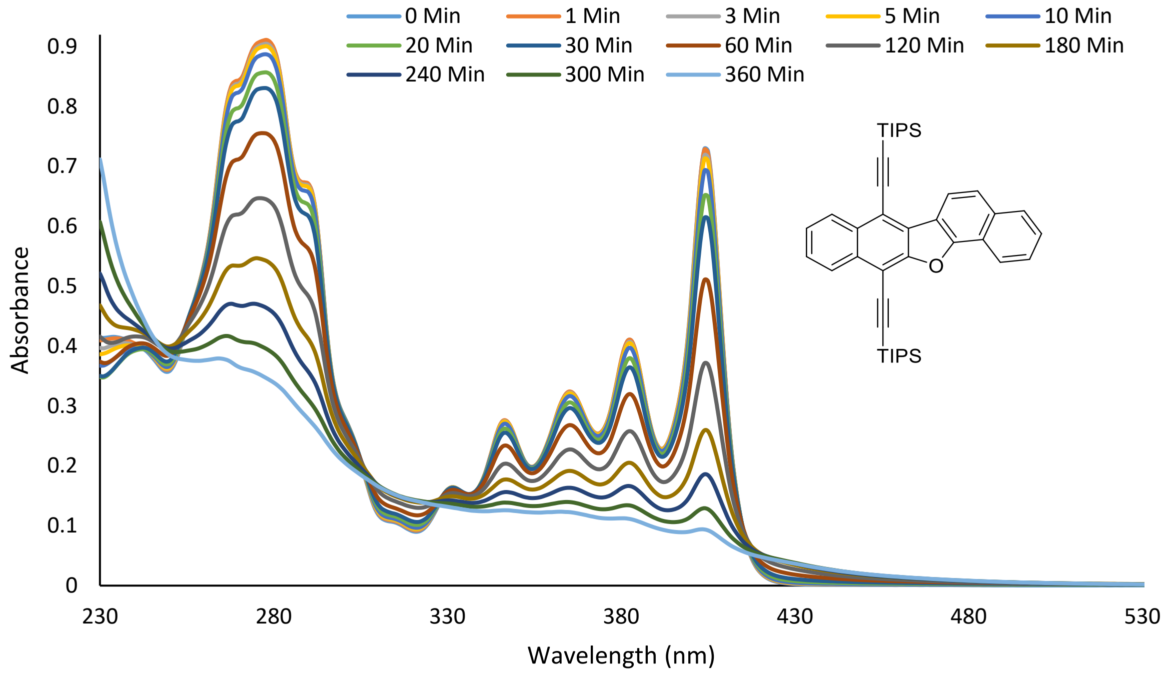

Figure S 12. Spectral changes upon irradiation of **4d** with 254 nm light (4W lamp) in aerated CH_2Cl_2.

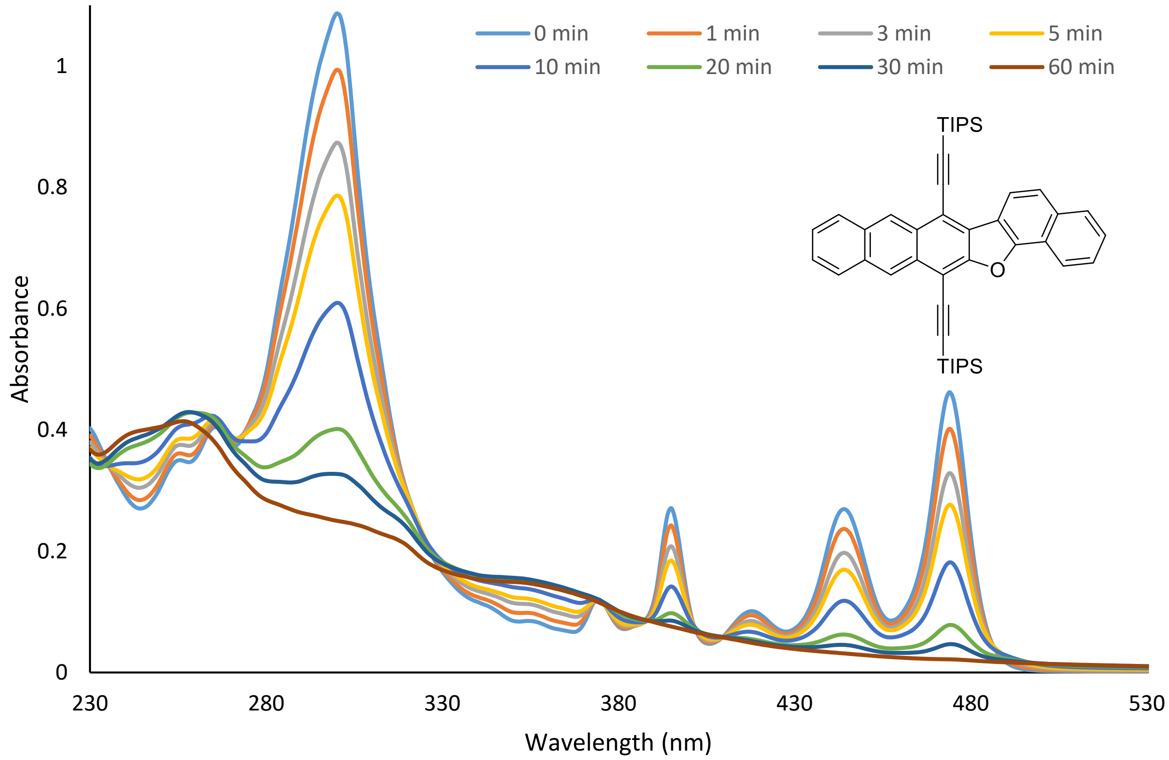

Figure S 13. Spectral changes upon irradiation of **4f** with 254 nm light (4W lamp) in aerated CH_2Cl_2.

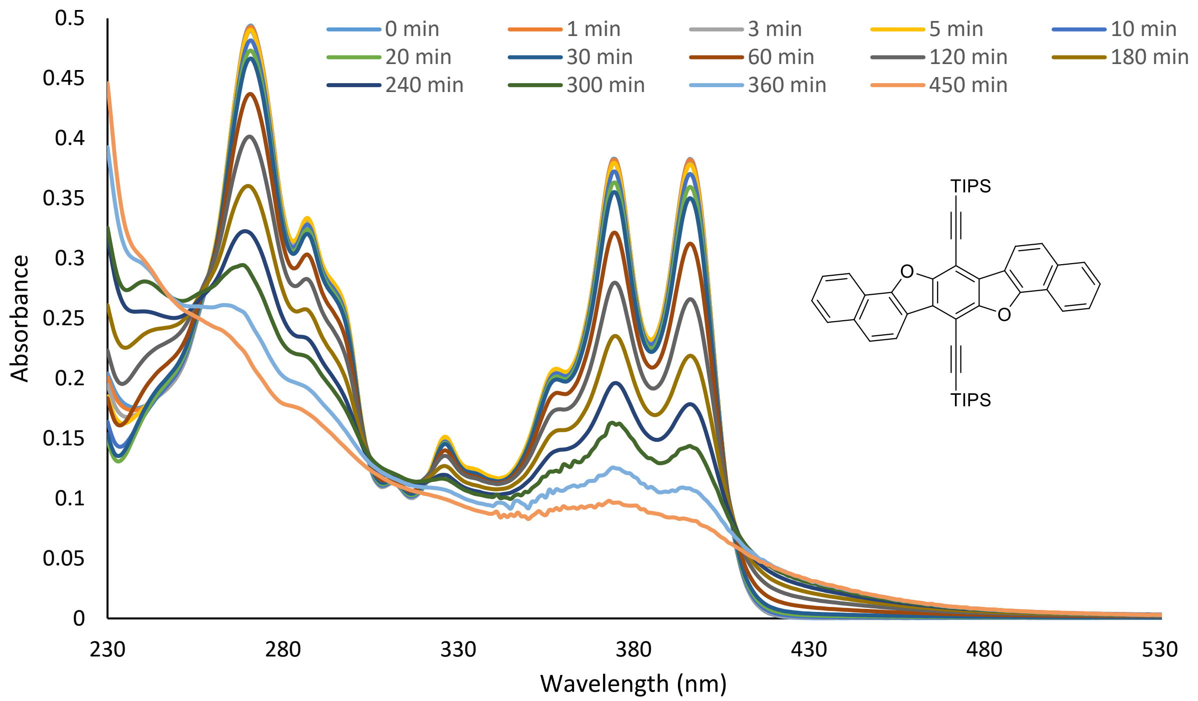

Figure S 14. Spectral changes upon irradiation of **4i** with 254 nm light (4W lamp) in aerated CH_2Cl_2.

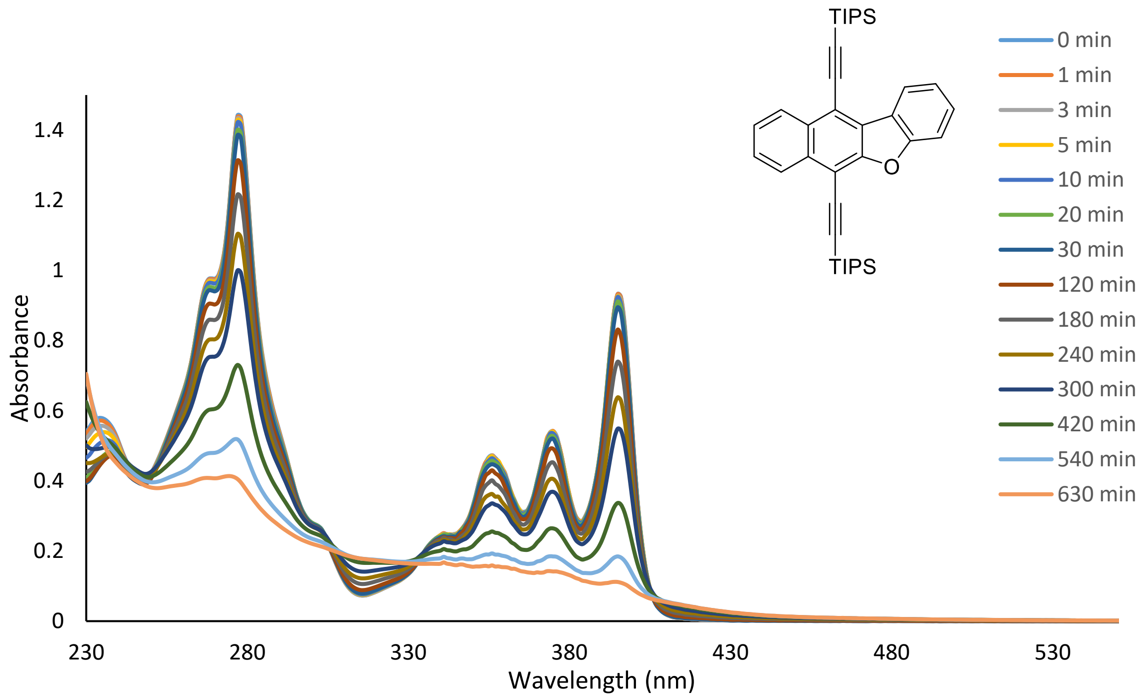

Figure S 15. Spectral changes upon irradiation of **4h** with 254 nm light (4W lamp) in aerated CH_2Cl_2.

S 6. Solvatochromism

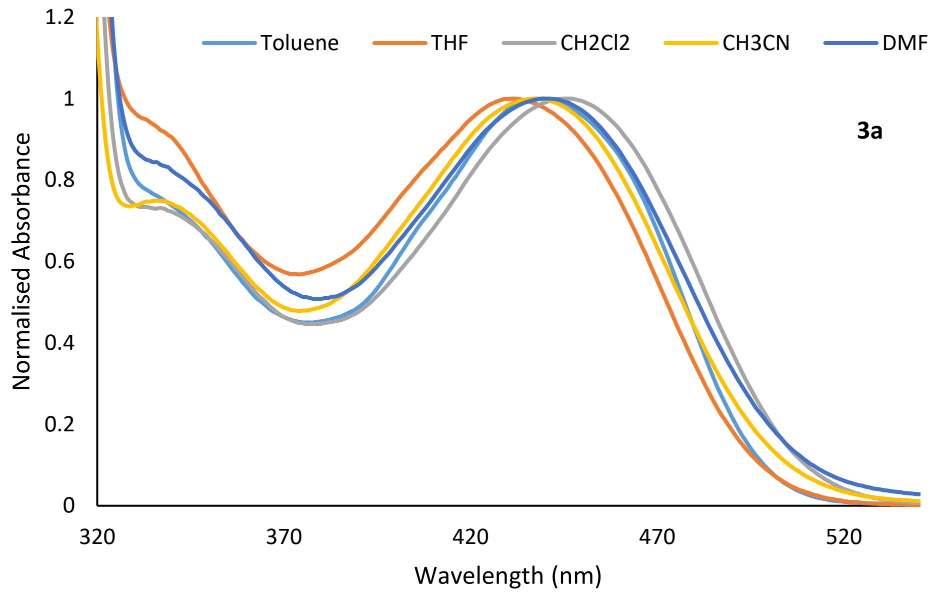

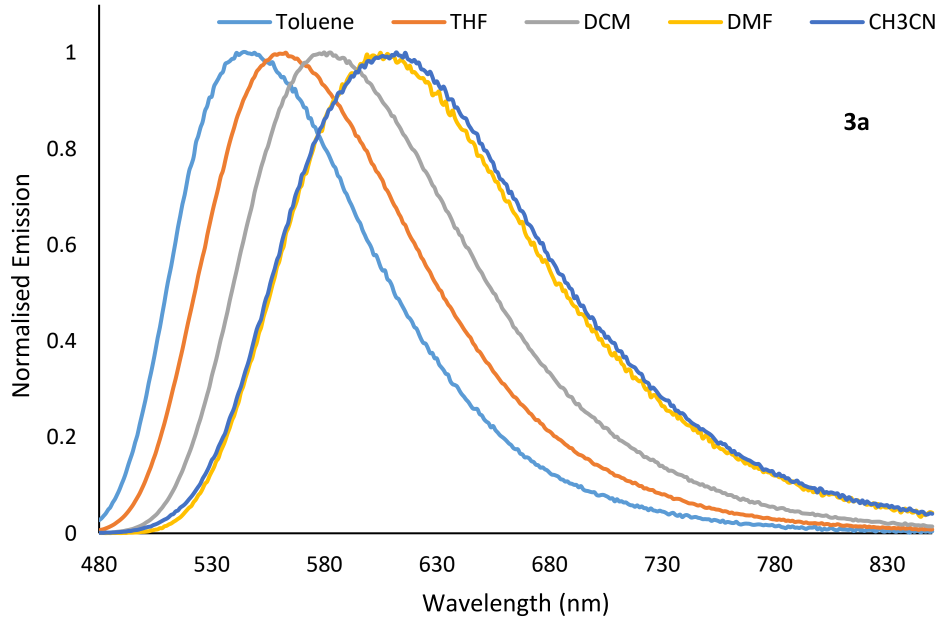

Figure S 16. Absorption and emission spectra of compound **3a** recorded in different solvents (toluene, THF, DCM, acetonitrile, and DMF), illustrating solvatochromism. The effect is especially evident in the emission spectra, which exhibit a pronounced bathochromic shift with increasing solvent polarity.

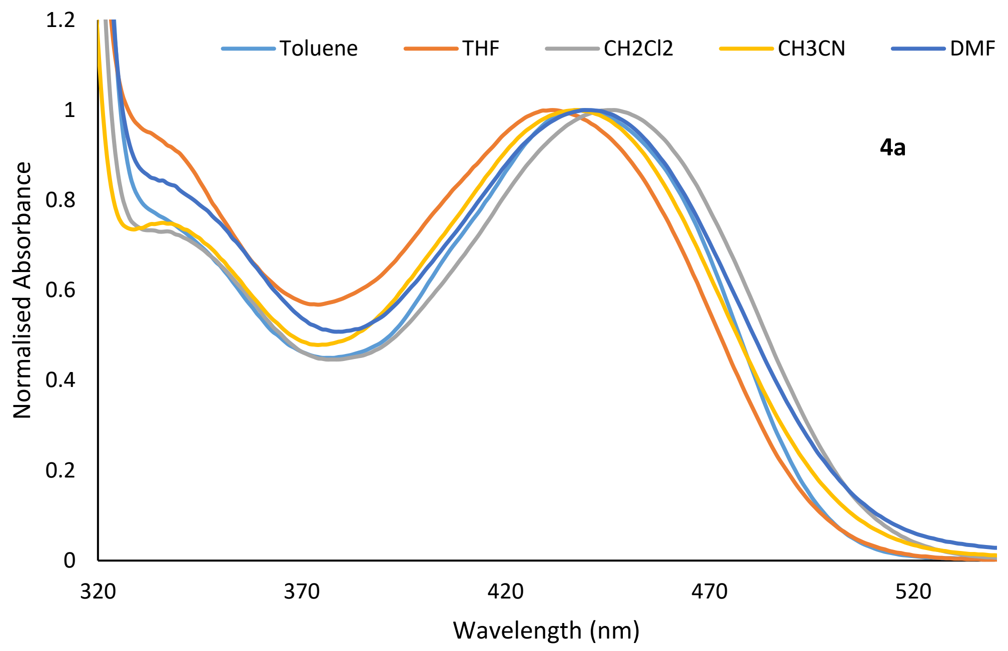

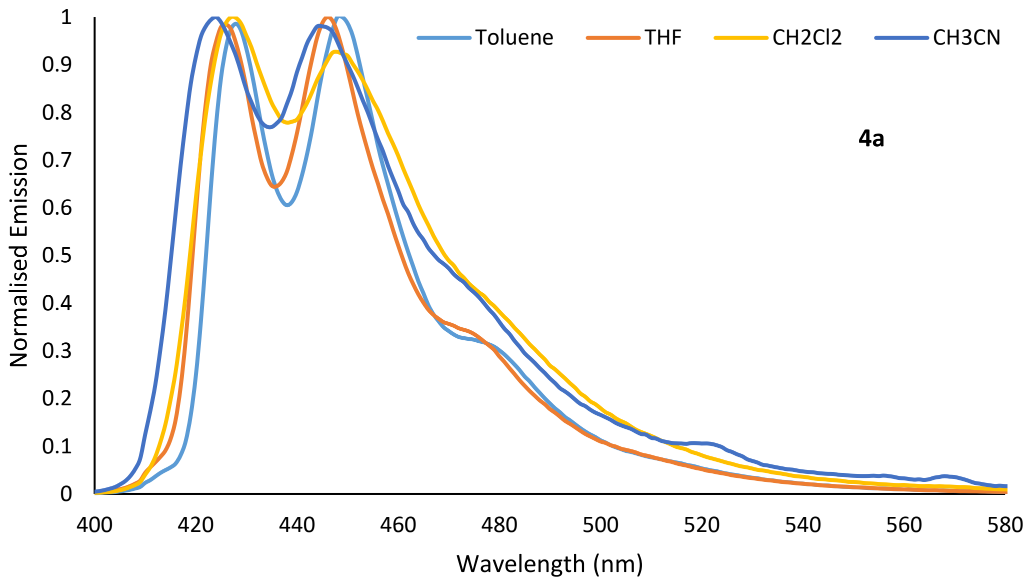

Figure S 17. Absorption and emission spectra of compound **4a** recorded in various solvents (toluene, THF, CH_2Cl_2, and CH_3CN) for the solvatochromism study.

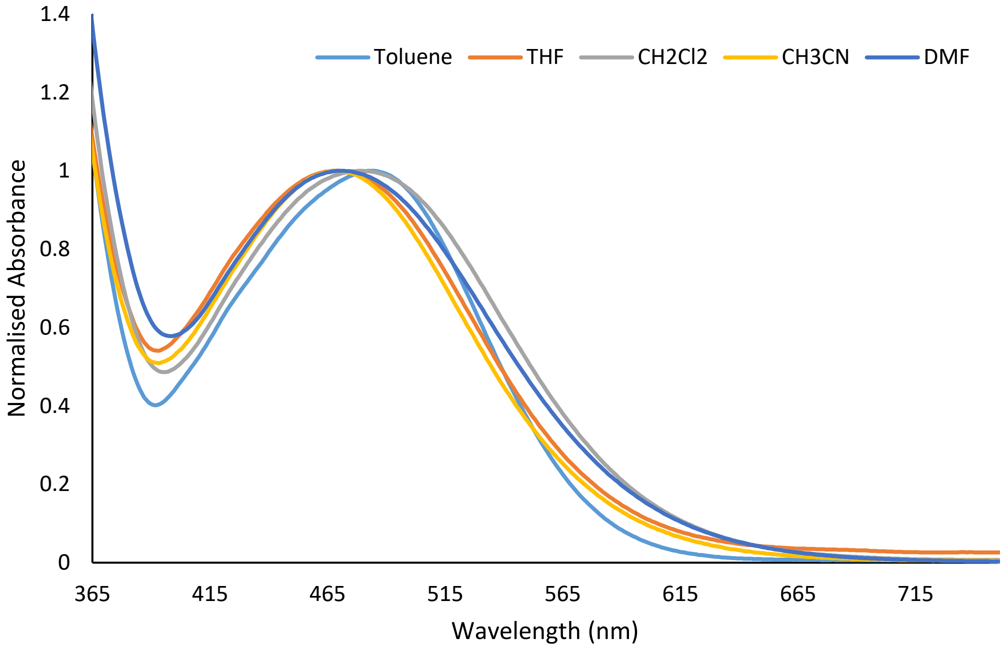

Figure S 18. Absorption compound **3l** recorded in different solvents (toluene, THF, CH_2Cl_2, CH_3CN, and DMF) for solvatochromism studies. The emission spectra of compound **3l** are inconclusive due to poor fluorescence intensity.

S 7. X-Ray Crystallographic Analysis

A fluorescent yellow needle-like crystals of **4i** specimen approximate dimensions 0.433 mm x 0.456 mm x 0.614 mm, was used for the X-ray crystallographic analysis. Crystal was grown by slow diffusion of hexane into a CH_2Cl_2 solution of **4i**. The X-ray intensity data were measured.

Data collections for **4i** were performed on Bruker X8 APEXII diffractometer at room temperature, using CuKα (λ = 1.54178 Å) radiation. Frames were integrated with the Bruker SAINT[3] software package using a narrow-frame algorithm. The structure was solved and refined using the Bruker SHELXTL Software Package[4]. Data were corrected for absorption effects using the face-indexed multi-scan method (SADABS)[5]. The structure was solved by direct methods SHELXS-2014 and refined with full-matrix least-squares calculations on F^2 using SHELX-2014[6].

Crystal data of **4i**: $C_{48}H_{54}O_2Si_2$, M_W = 719.09 g mol^{-1}, monoclinic space group C2/c, a = 18.404(2), b = 18.008(2), c = 15.0545(19) Å, β = 121.735(7)° V = 4243.4(9) $Å^3$, F(000) = 1544, d_{calc} = 1.126 g cm^{-3}, Z = 4, μ = 1.027 mm^{-1}, T = 296(2) K, 28062 reflections measured and corrected for multi-scan absorption with T_{min} = 0.571 and T_{max} = 0.665, 2831 reflections were unique (R_{int} = 0.1527), final R_1 = 0.1070, wR_2 = 0.3117, for 1159 observed reflections with [I > 2σ(I)]; GOF = 1.030. All hydrogen atoms were placed in calculated positions and refined as riding on their parent atoms with U_{iso} = 1.2 Ueq (N, C). All non-hydrogen atoms were refined anisotropically.

Crystallographic data have been deposited at the Cambridge Crystallographic Data Centre, 12 Union Road, 129 Cambridge CB21EZ, UK, and copies can be obtained on request, free of charge, by quoting the publication citation and the deposition number. CCDC number for **4i:** 2434127

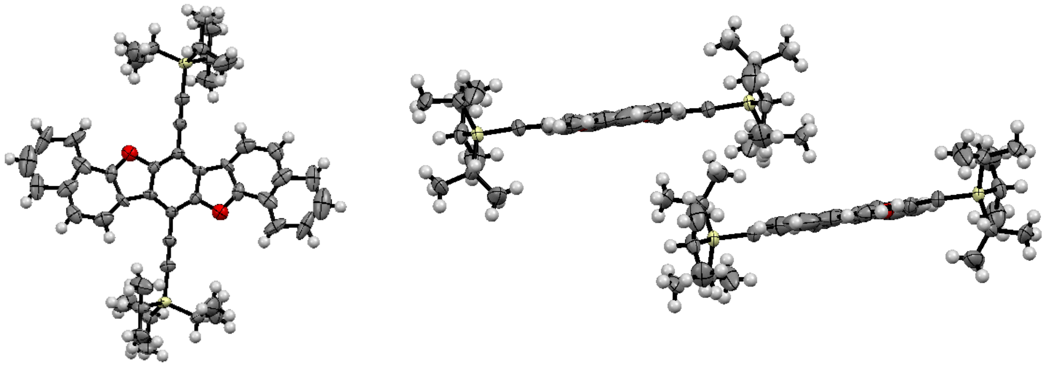

Figure S 19: ORTEP plot for single crystal structure of **4i**. Atomic displacement parameters at 296 K are drawn at 30 % probability level.

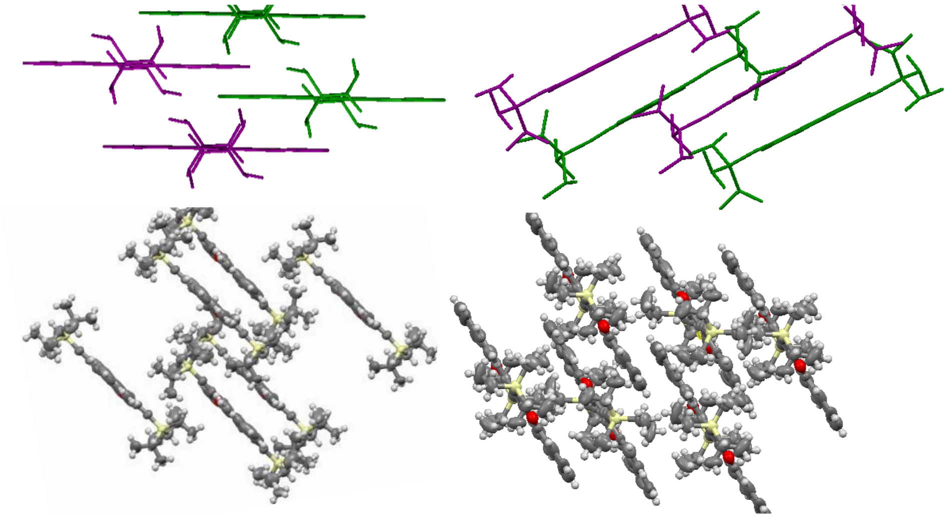

Figure S 20: Crystal packing of compound **4i** in the solid state, illustrating intermolecular interactions and molecular orientation within the unit cell.

S 8. Quantum Chemical Calculations

Calculations were performed using **Gaussian** software. Geometry optimizations and frequency calculations were carried out at the **CAM-B3LYP**[10]**/def2-TZVP**[11] level of theory. Vibrational frequency calculations were performed to confirm that optimized structures correspond to true minima (no imaginary frequencies). To account for solvation effects, the polarizable continuum model (PCM) was employed using dichloromethane as the solvent.

Table S 3. **HOMO** (Highest Occupied Molecular Orbital) and **LUMO** (Lowest Unoccupied Molecular Orbital) energies of the optimized structures are summarized. These values were obtained from the **CAM-B3LYP/def2-TZVP** calculations in dichloromethane using the **self-consistent reaction field (SCRF) method**.

Name	structure	HOMO	LUMO
4a	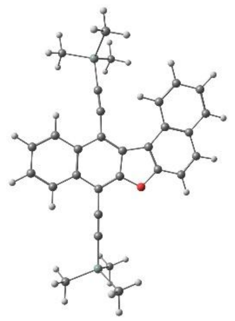	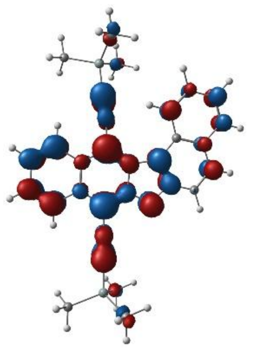	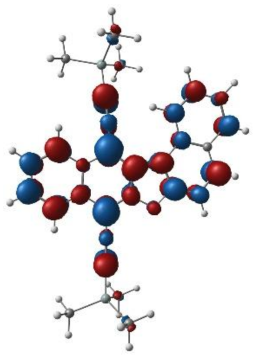
4b	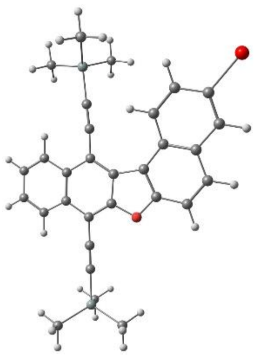	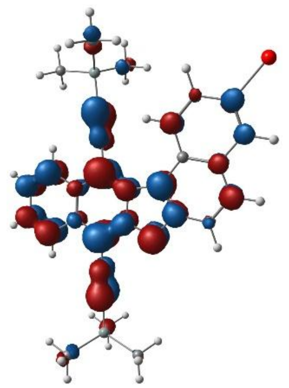	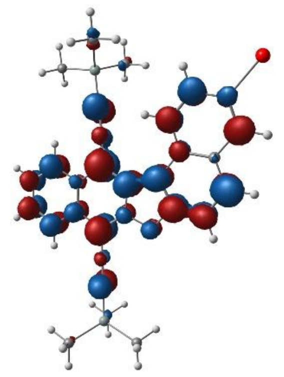
4c	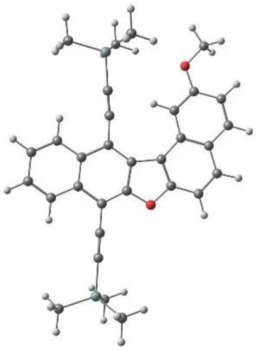	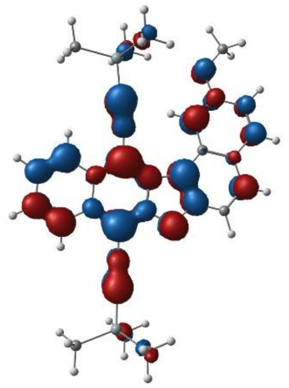	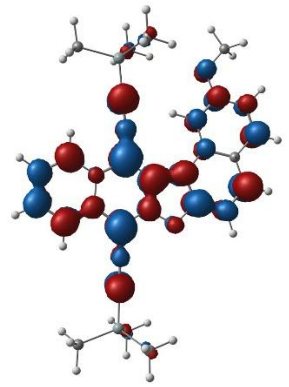

**4d**	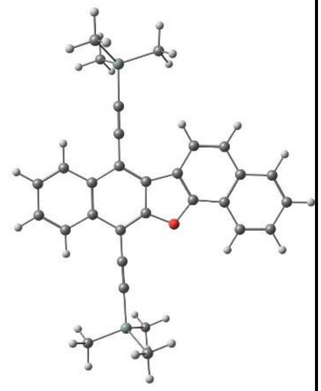	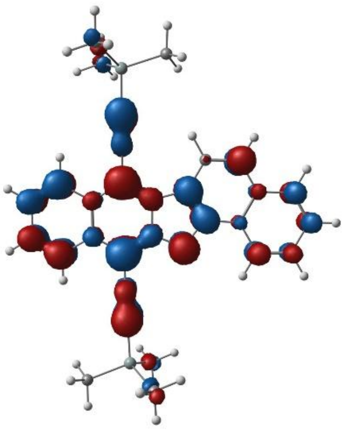	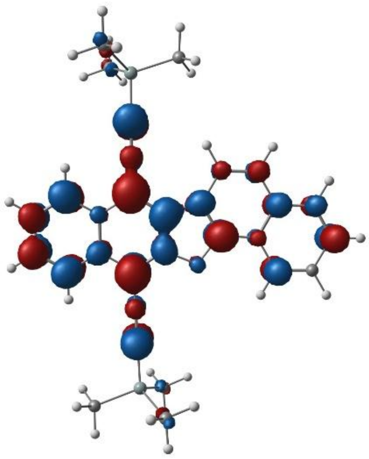
**4e**	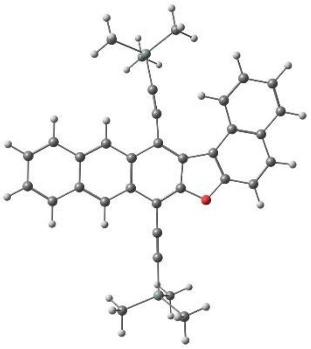	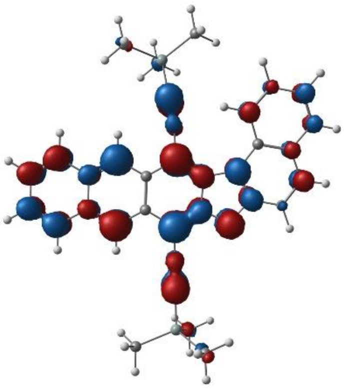	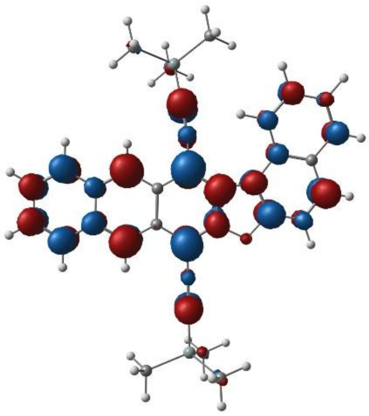
**4f**	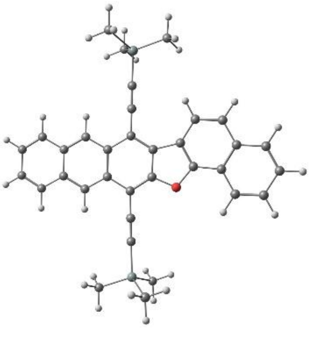	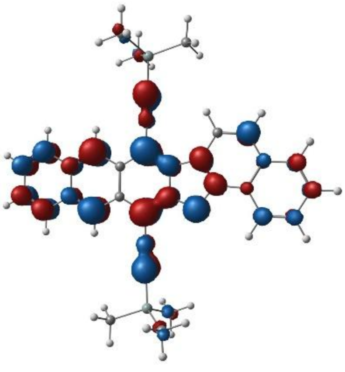	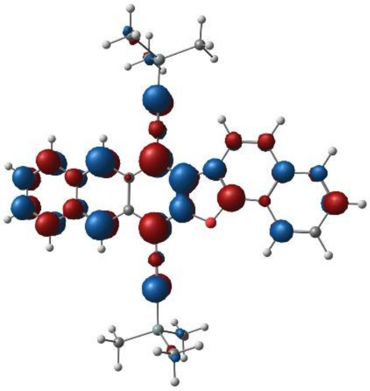
**4g**	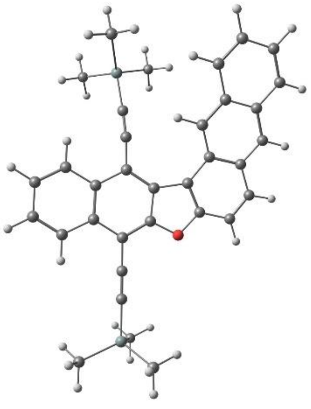	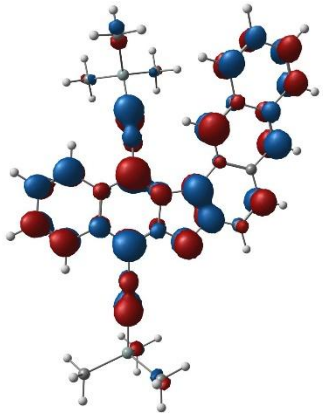	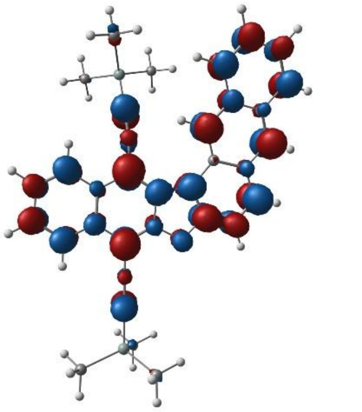

**4h**	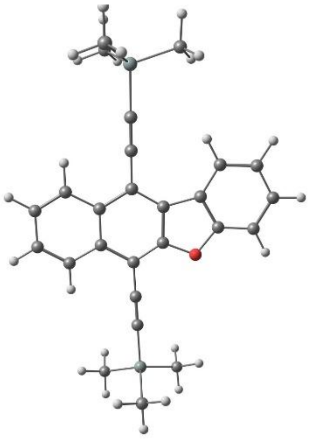	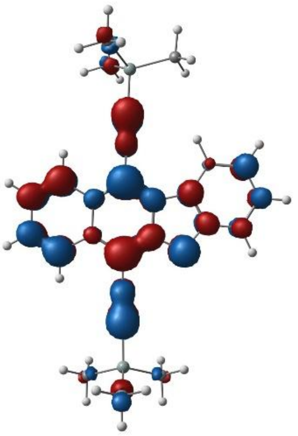	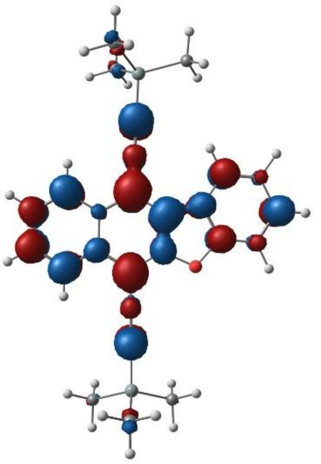
**4i**	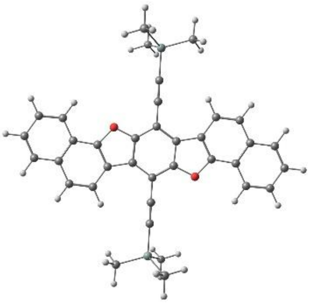	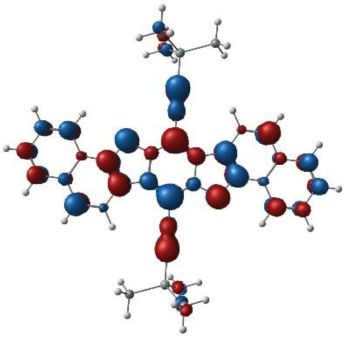	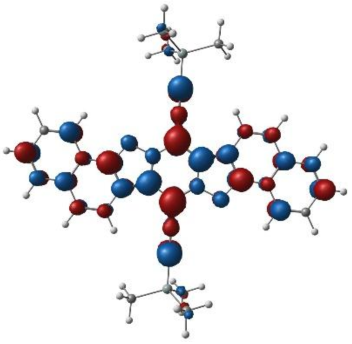
**4j**	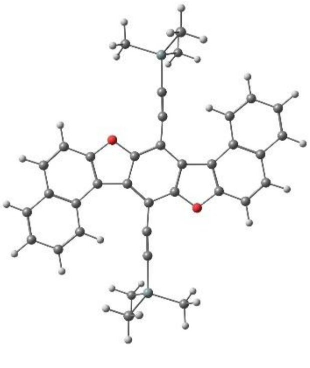	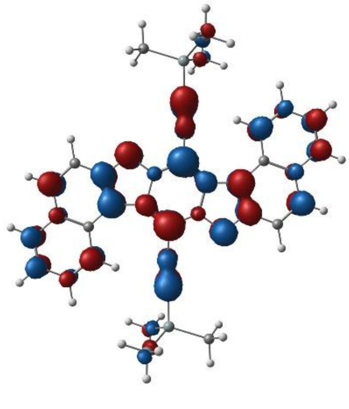	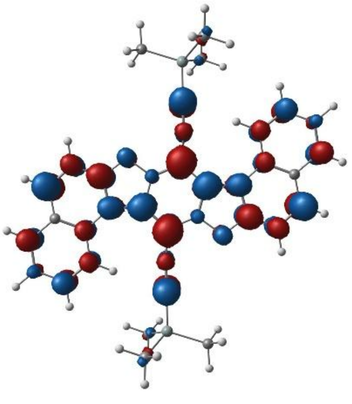
**4k**	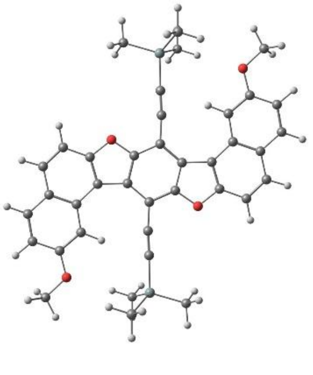	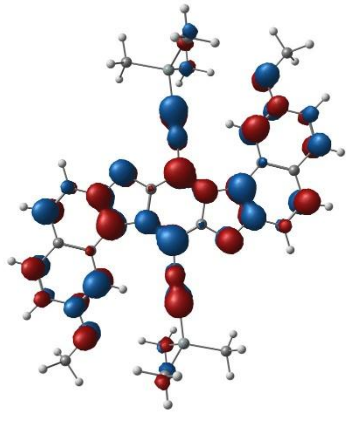	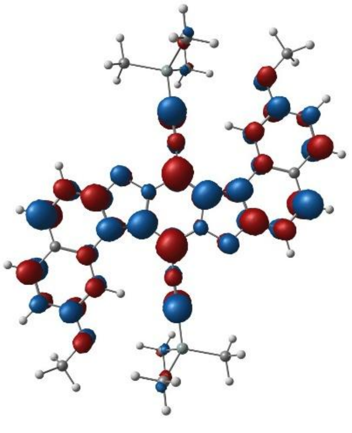

**3a**	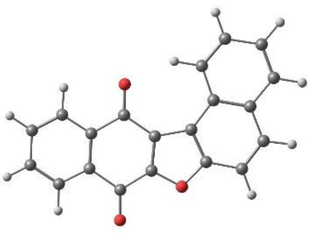	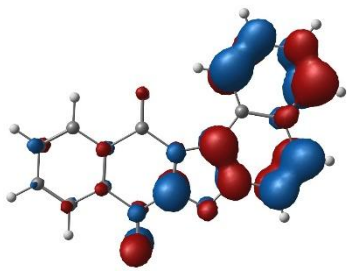	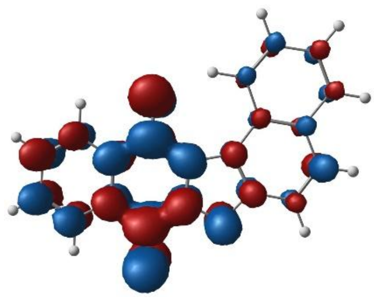
**3l**	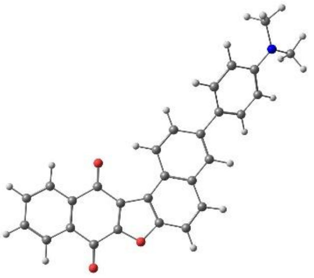	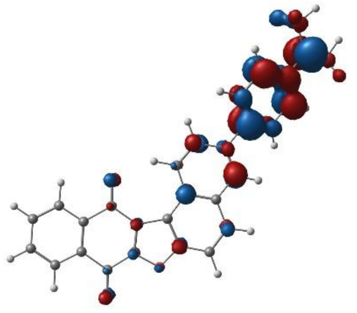	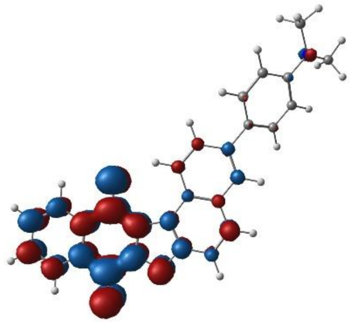

Table S 4. Calculated parameters of HOMO-to-LUMO transitions of selected compounds

**Compounds**	**Transition energy/nm**	**Transition energy/ev**	**Oscillator strength f**	**Transition FMOs**
**3a**	394.74	3.1409	0.194	75 -> 78 -0.22992<br>77 -> 78 0.65658
**3l**	411.51	3.0129	0.3109	105 -> 110 -0.14868<br>108 -> 110 0.46898<br>109 -> 110 0.49059
**4a**	379.87	3.2638	0.4833	122 -> 123 0.69325
**4b**	379.88	3.2638	0.4788	139 -> 140 0.69194
**4c**	380.50	3.2584	0.4212	130 -> 131 0.69197
**4d**	371.41	3.3382	0.6661	122 -> 123 0.69179
**4e**	445.15	2.7852	0.3625	135 -> 136 0.69749
**4f**	436.88	2.8379	0.4534	135 -> 136 0.69734
**4g**	404.46	3.0654	0.3415	135 -> 136 0.68659
**4h**	361.38	3.4309	0.6172	109 -> 110 0.69566
**4i**	353.99	3.5025	1.0266	145 -> 146 0.68370
**4j**	368.24	3.3669	0.6958	145 -> 146 0.68465
**4k**	375.84	3.2989	0.7207	159 -> 163 -0.10020<br>161 -> 162 0.68382

S 9. Calculations of Nucleus Independent Chemical Shift (NICS) Plots

Nucleus-independent chemical shifts (NICS) were computed to investigate the electronic properties and aromaticity of the studied polycyclic furans. Triisopropylsilyl (TIPS) groups were replaced with trimethylsilyl (TMS) groups to reduce computational cost. These calculations provide insights into the magnetic shielding effects associated with aromatic systems, which are critical for understanding the properties of these compounds. NICS values were initially calculated at a distance of 1 bohr (0.53 Å) above the plane of the polycyclic aromatic core, as this position maximizes the detection of ring currents due to the close proximity to the π-electron cloud; and at 1.7 Å above the aromatic plane because, at 0.53 Å, the influence of σ-electrons and localized bonding interactions can become significant, potentially leading to misleading interpretations of aromaticity. According to Stanger and Gershoni-Poranne, at the 1.7 Å height, these σ-bond effects are minimized, allowing for a more accurate assessment of the π-electron contributions to the aromatic character.[12]

NICS calculations were performed using Gaussian software at the B3LYP/def2-TZVPP level. A grid of ghost atoms (Bq) was placed 0.2 Å apart at a specified height above the molecular plane, where GIAO NMR calculations were performed to capture the aromaticity effects. Input files were generated using py.Aroma software.[13] The extracted isovalues were then visualized using a custom-built Python script, which employs the following libraries:

- **Pandas**: For reading and manipulating data from Excel files (*pd.read_excel()*).
- **Matplotlib**: For creating and displaying 2D grid plots (*plt.contourf(), plt.colorbar()*, etc.).
- **NumPy**: For numerical operations, including creating grids for plotting (*np.mgrid()*).
- **SciPy**: Specifically, the *griddata* function from the *scipy.interpolate* module is used to interpolate data points onto a grid for contour plotting.

Below, graphical representations of these results for the **3a, 3l** and **4a-4k** of the studied polycyclic furans are presented.

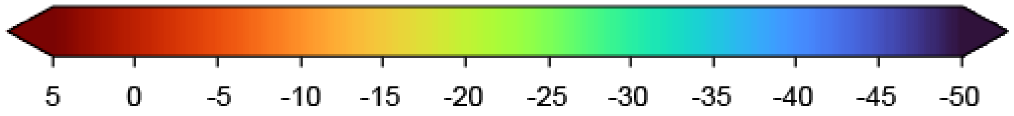

Figure S 21. Isovalue scale for NICS plots at the height 0,53 Å above the molecular plane.

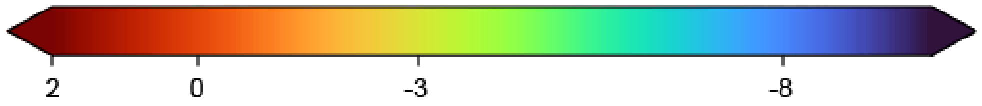

Figure S 22. Isovalue scale for NICS plots at the height 1.7 Å above the molecular plane.

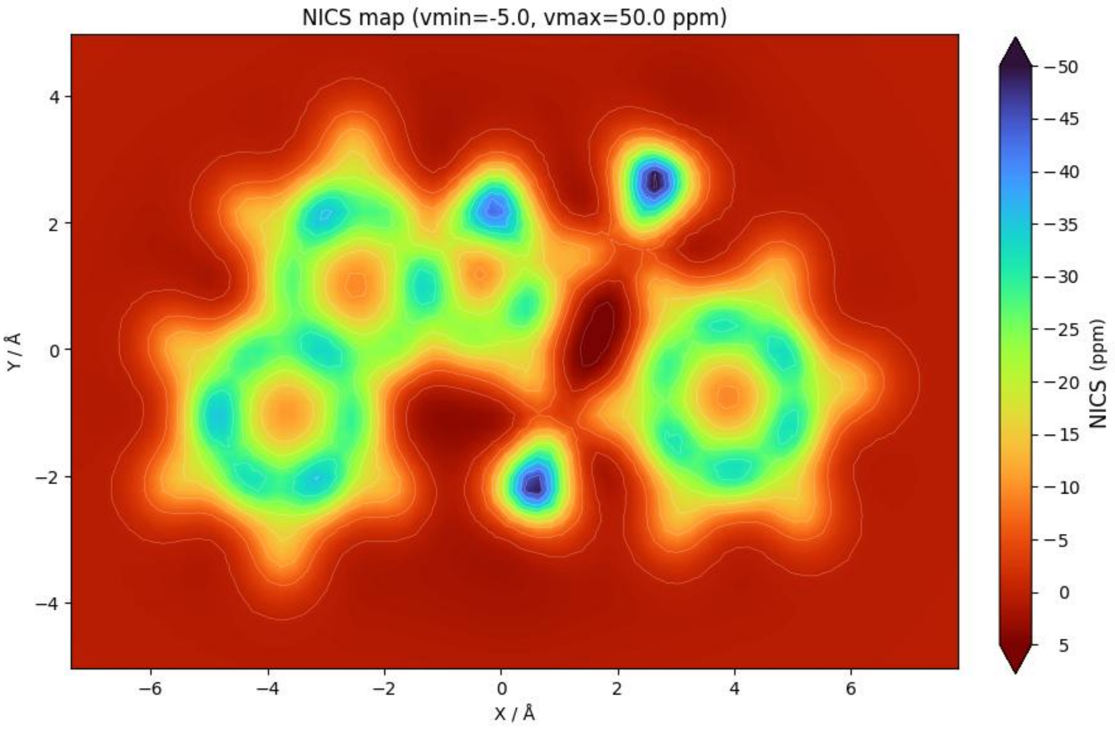

Figure S 23. NICS grid plot 0.53 Å above the molecular plane of **3a**.

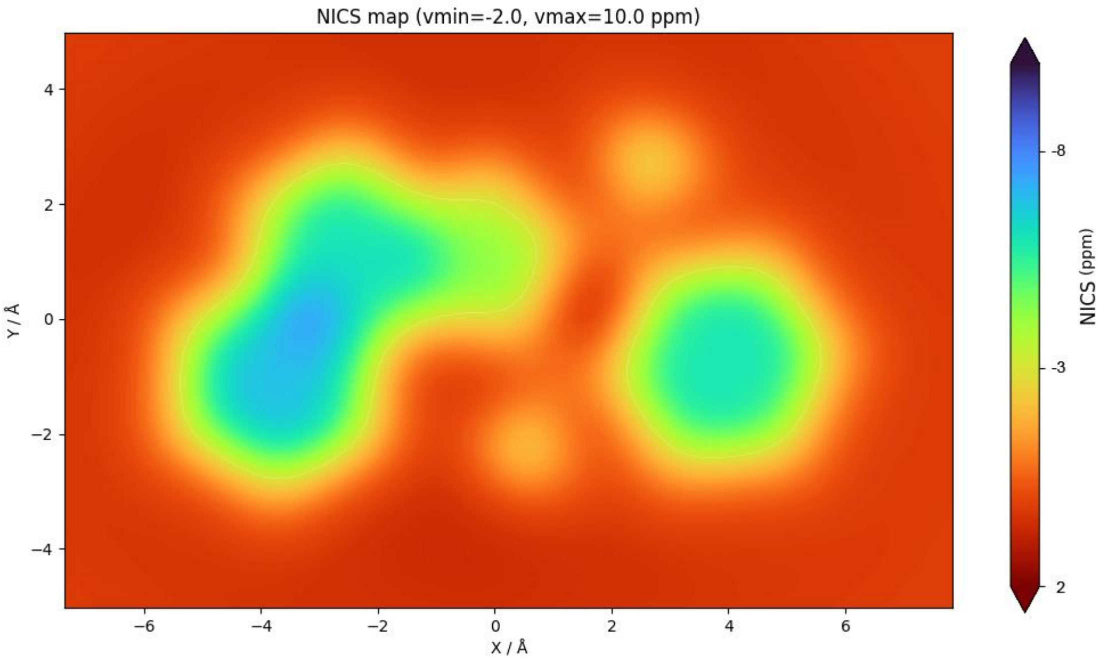

Figure S 24. NICS grid plot 1.7 Å above the molecular plane of **3a**.

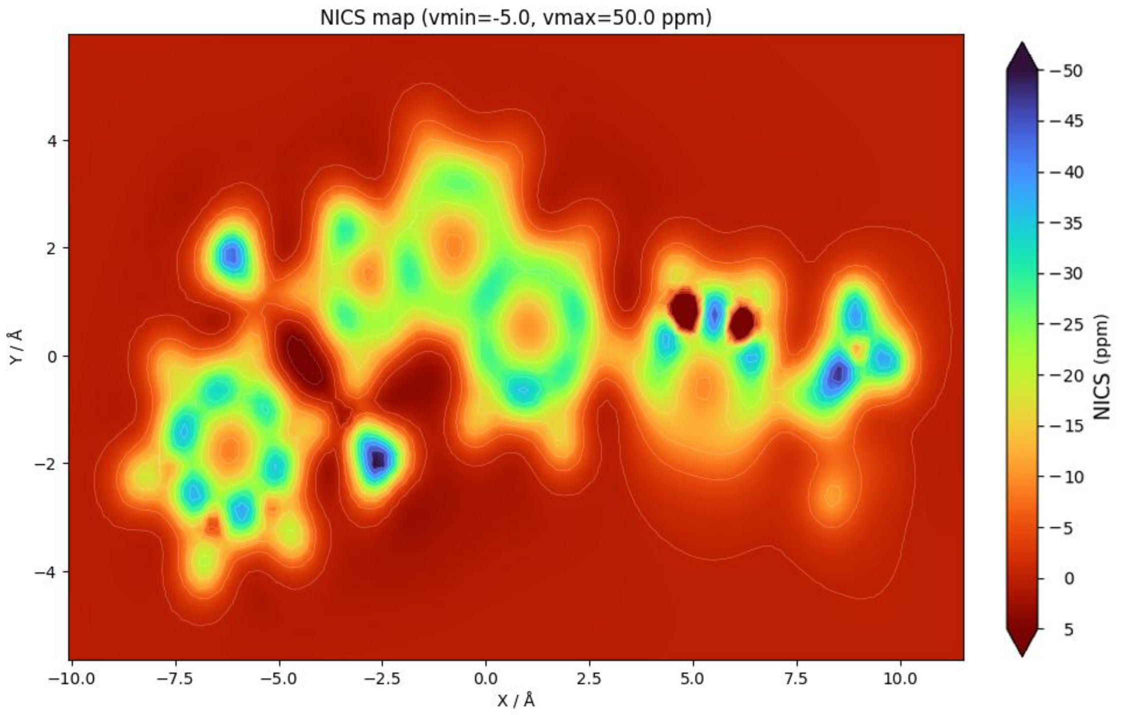

Figure S 25. NICS grid plot 0.53 Å above the molecular plane of **3l**.

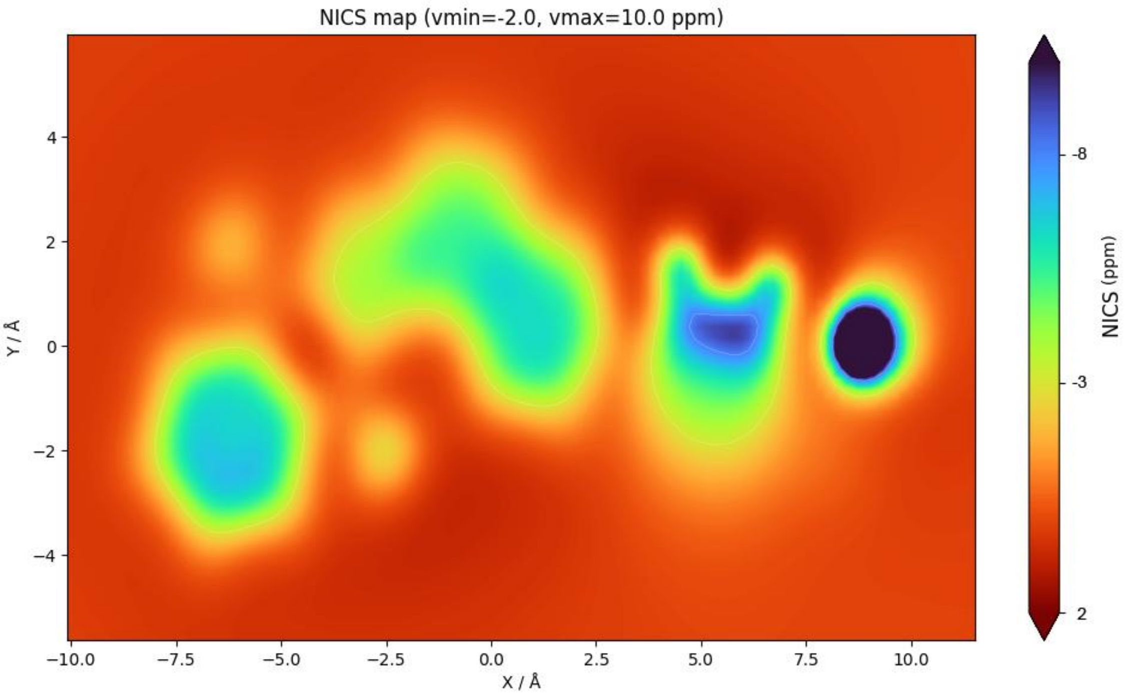

Figure S 26. NICS grid plot 1.7 Å above the molecular plane of **3l**.

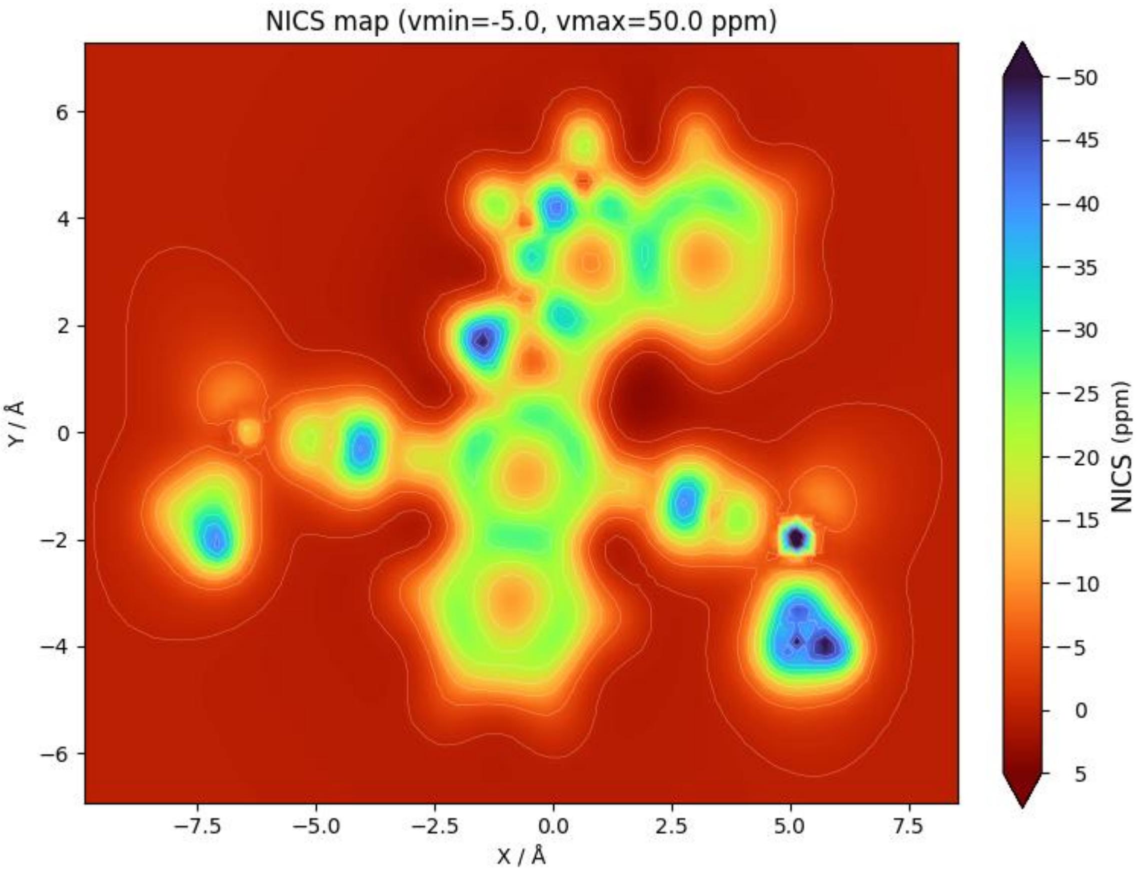

Figure S 27. NICS grid plot 0.53 Å above the molecular plane of **4a**.

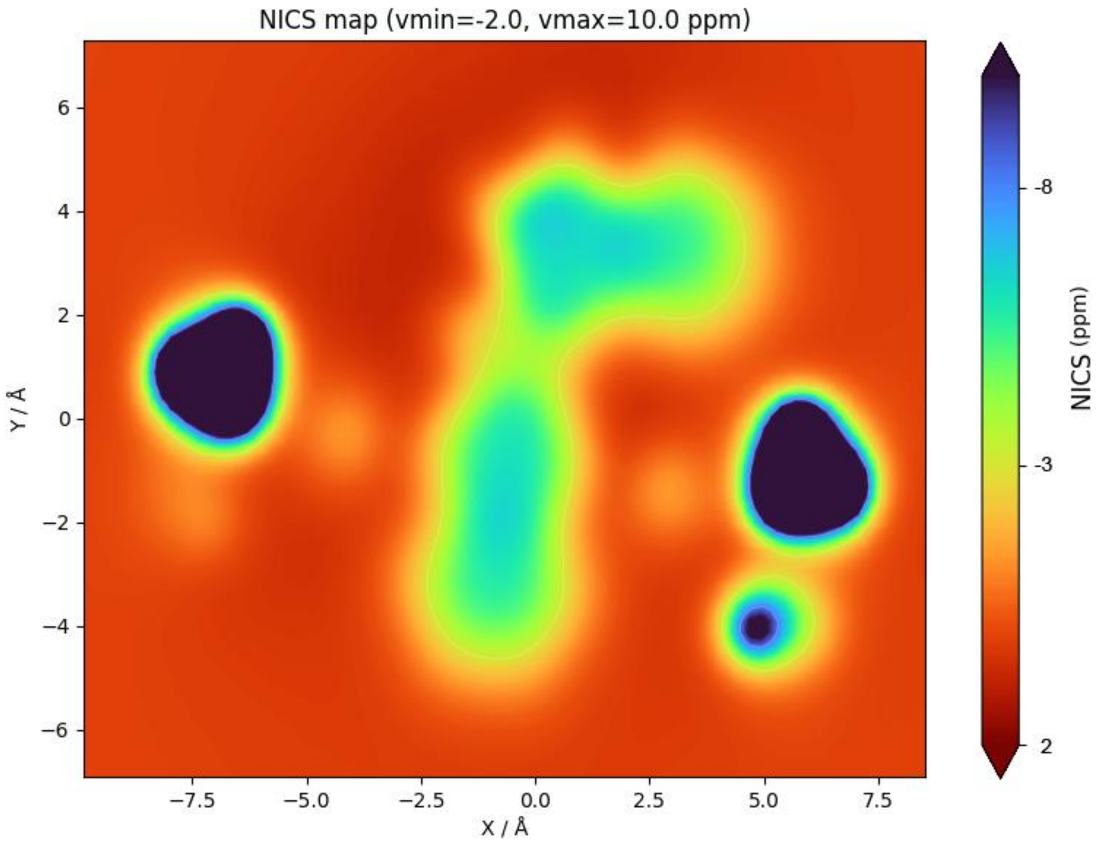

Figure S 28. NICS grid plot 1.7 Å above the molecular plane of **4a**.

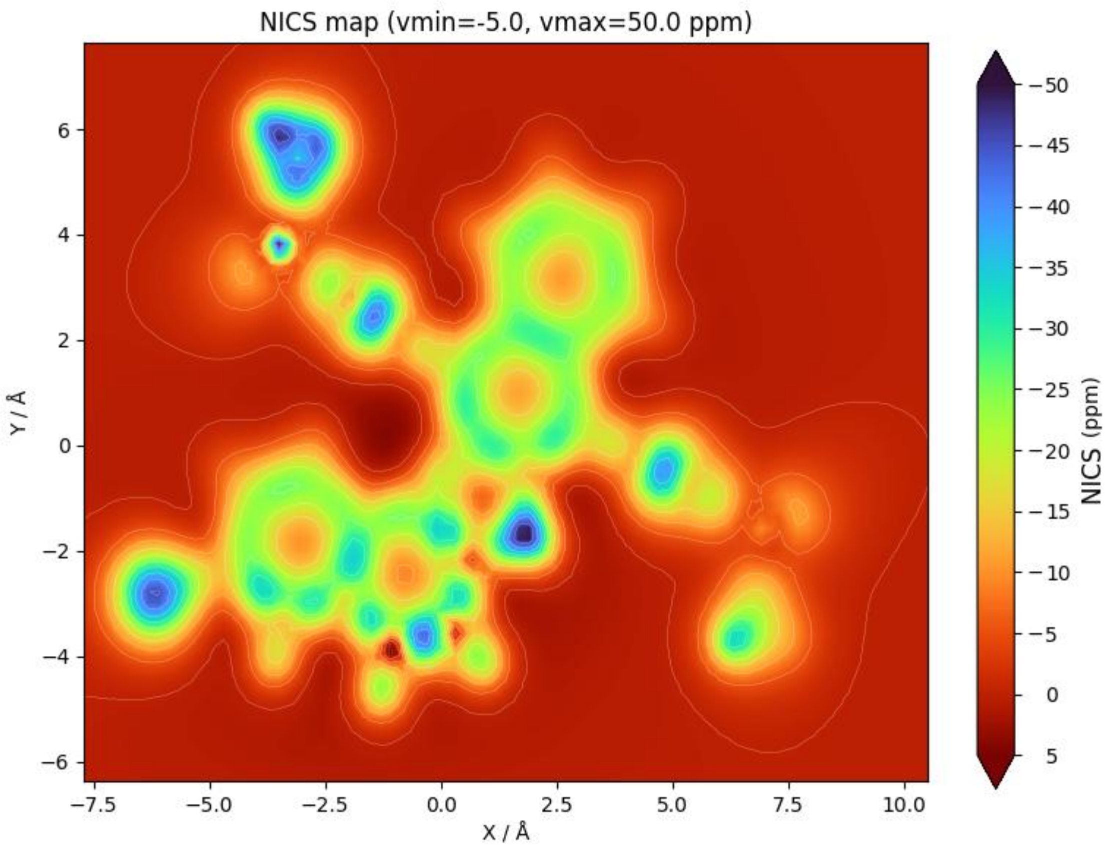

Figure S 29. NICS grid plot 0.53 Å above the molecular plane of **4b**.

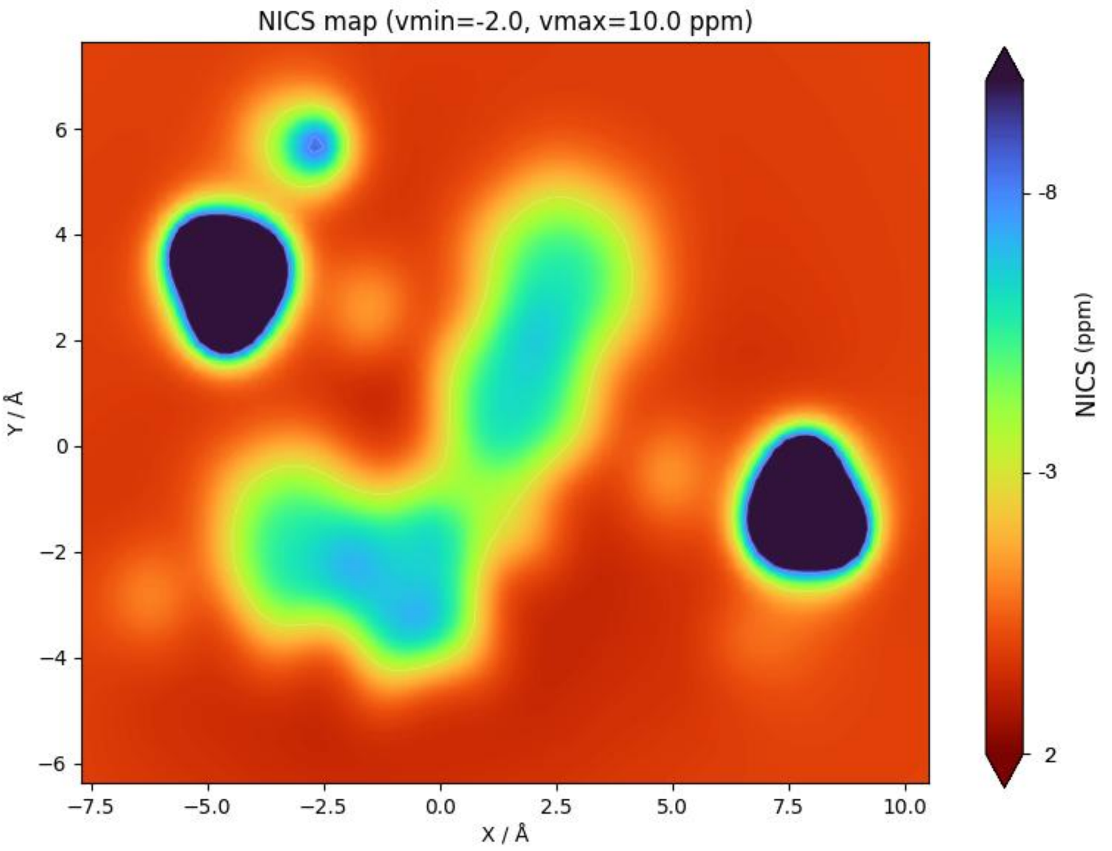

Figure S 30. NICS grid plot 1.7 Å above the molecular plane of **4b**.

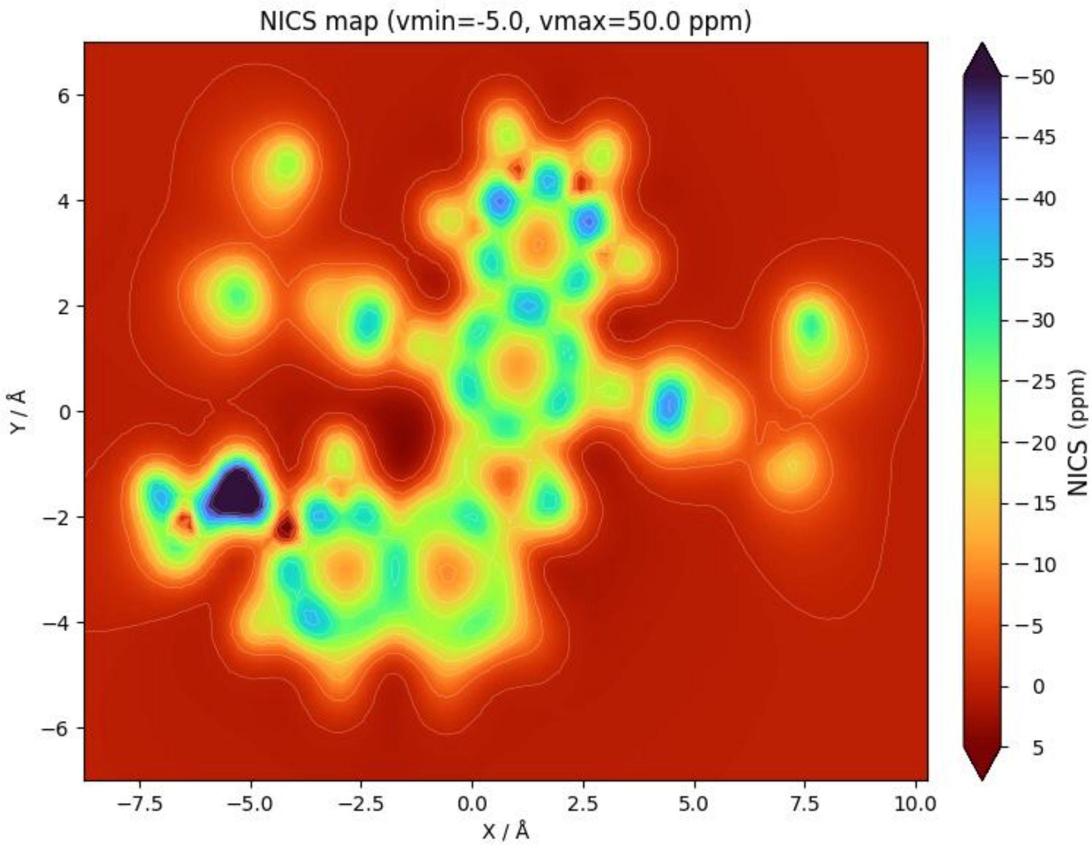

Figure S 31. NICS grid plot 0.53 Å above the molecular plane of **4c**.

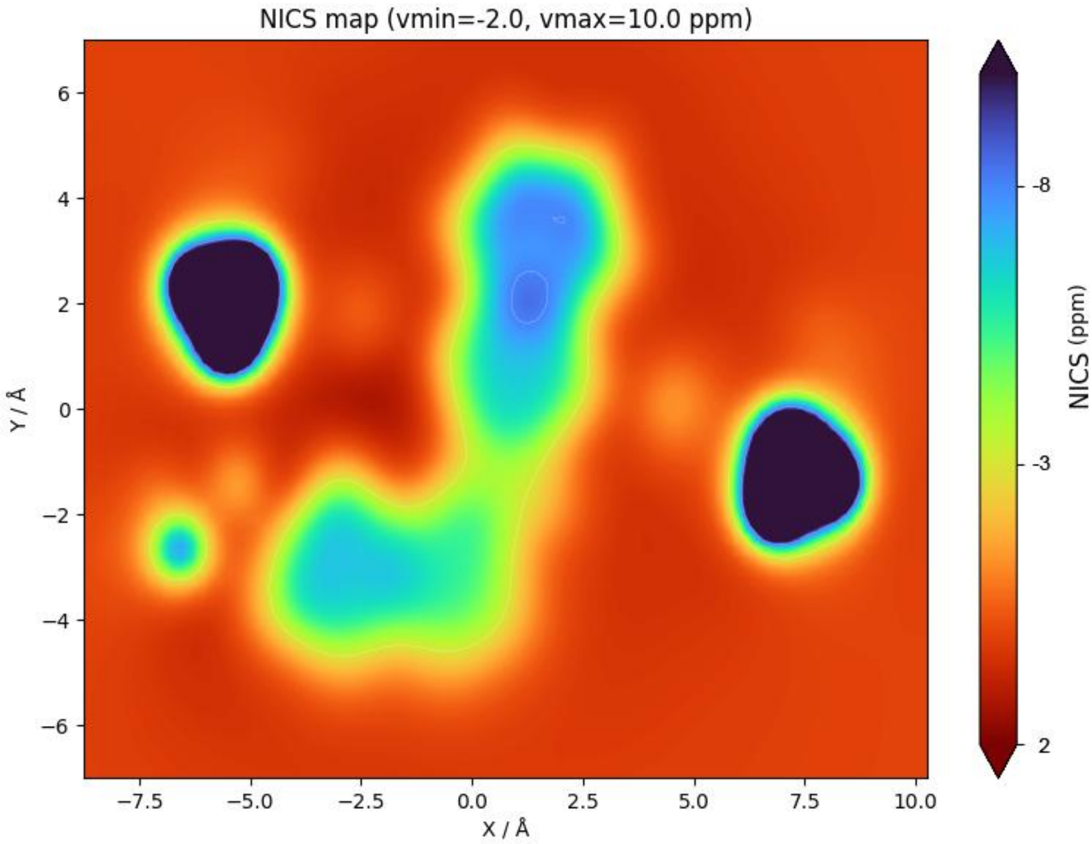

Figure S 32. NICS grid plot 1.7 Å above the molecular plane of **4c**.

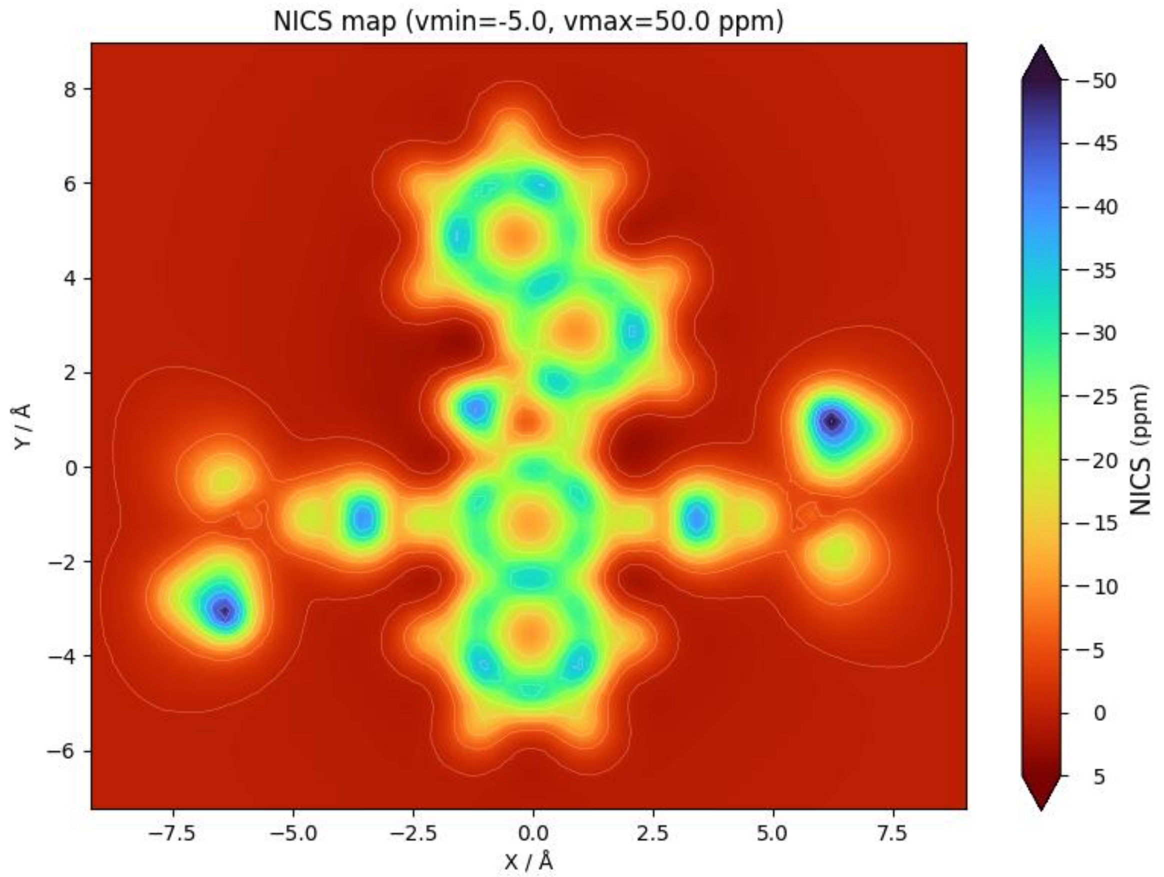

Figure S 33. NICS grid plot 0.53 Å above the molecular plane of **4d**.

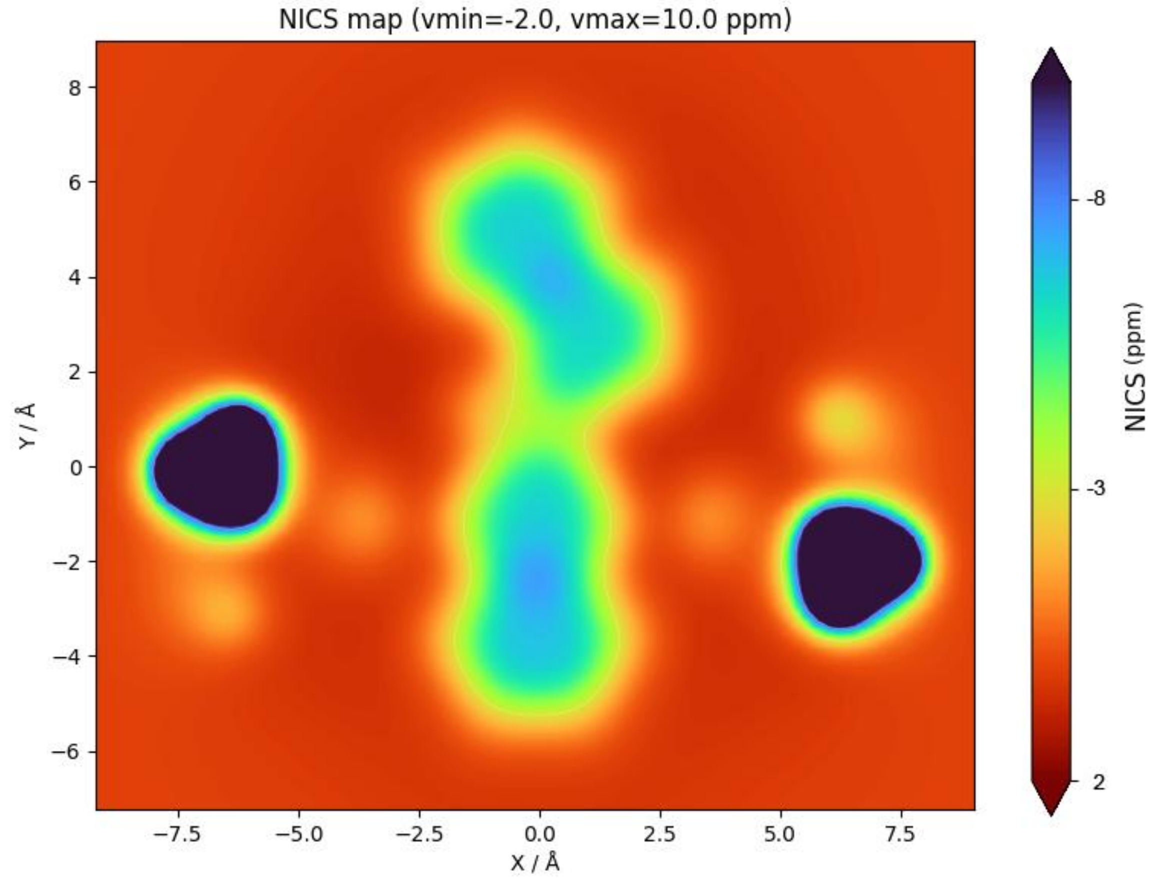

Figure S 34. NICS grid plot 1.7 Å above the molecular plane of **4d**.

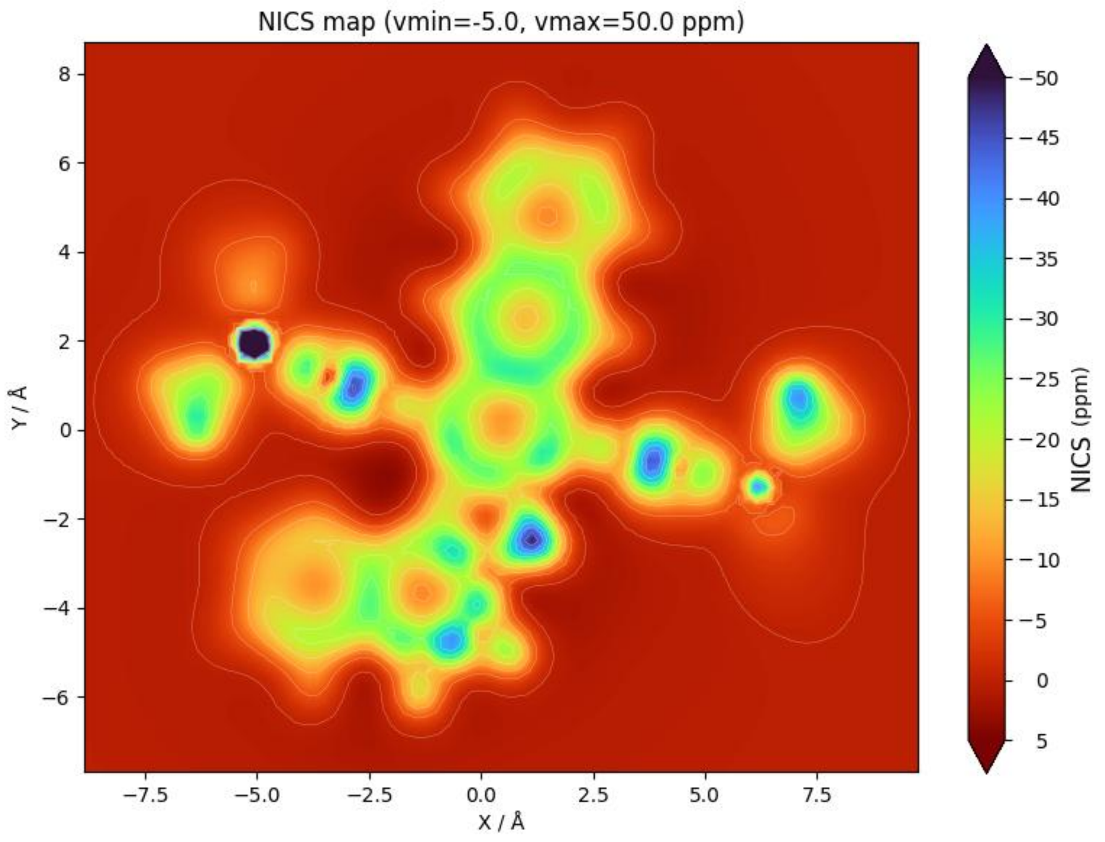

Figure S 35. NICS grid plot 0.53 Å above the molecular plane of **4e**.

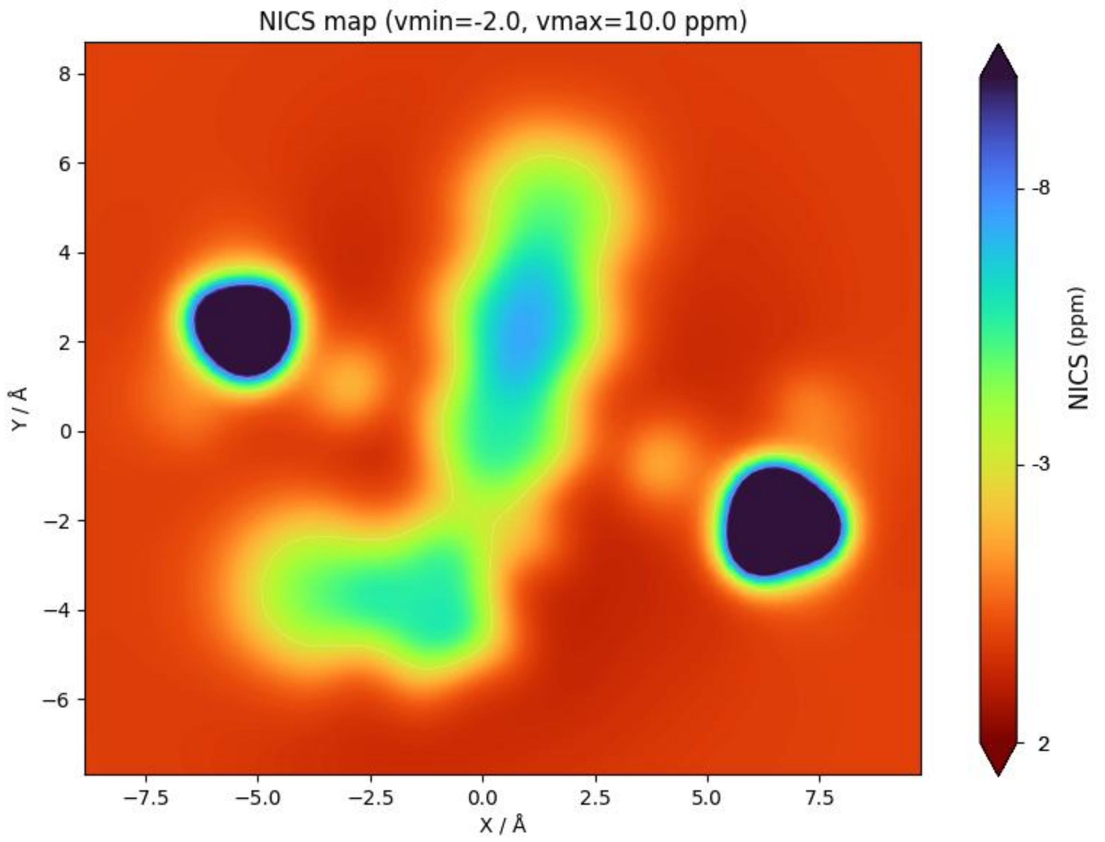

Figure S 36. NICS grid plot 1.7 Å above the molecular plane of **4e**.

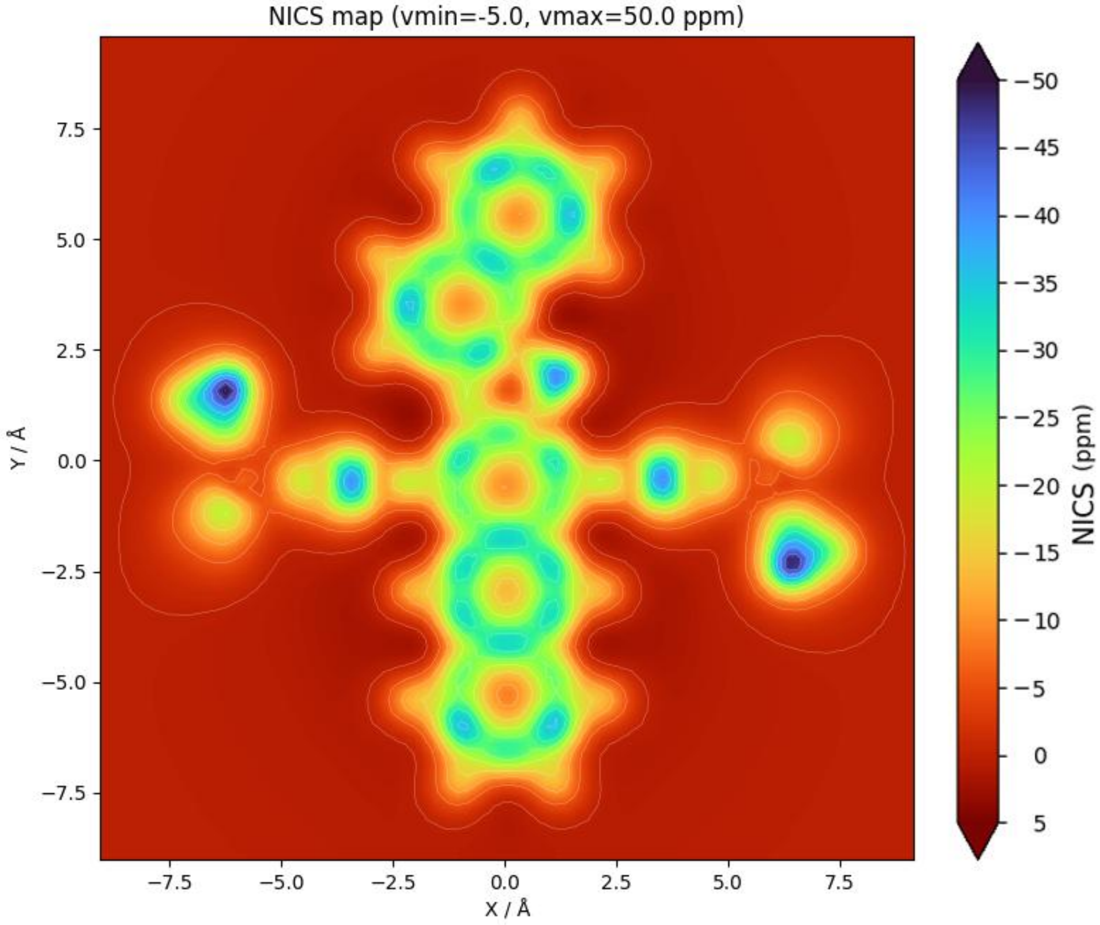

Figure S 37. NICS grid plot 0.53 Å above the molecular plane of **4f**.

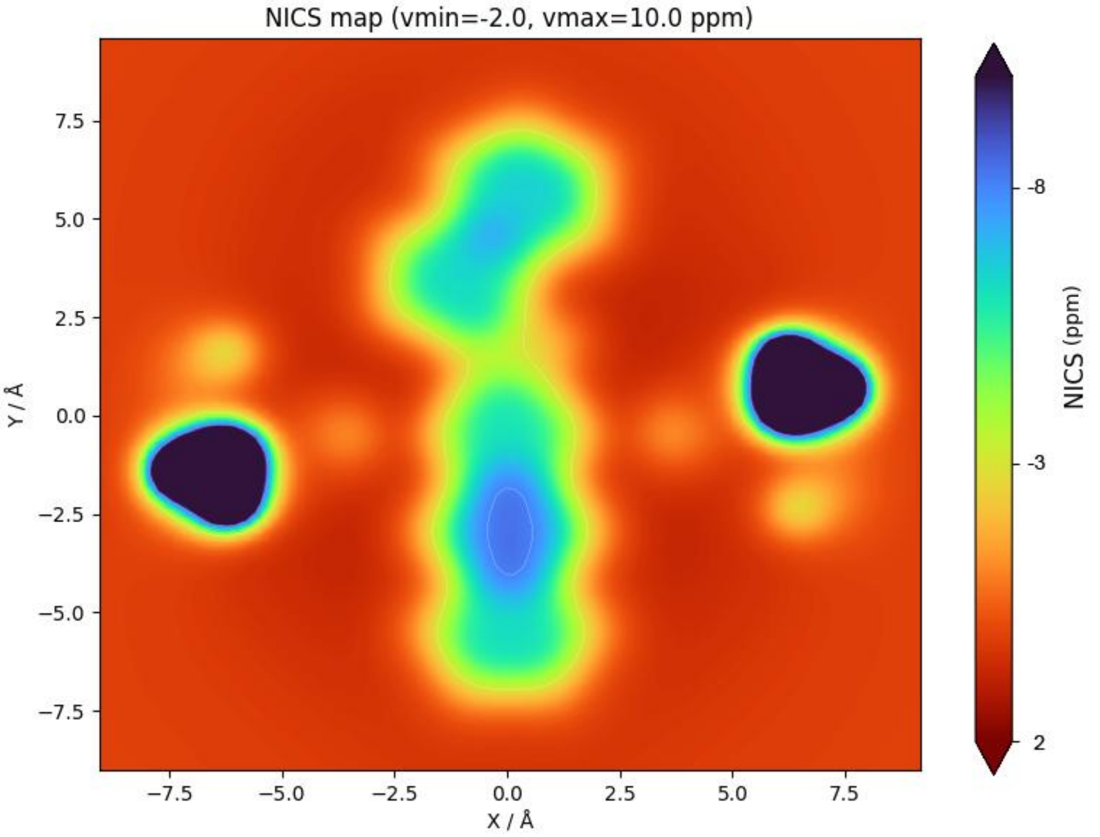

Figure S 38. NICS grid plot 1.7 Å above the molecular plane of **4f**.

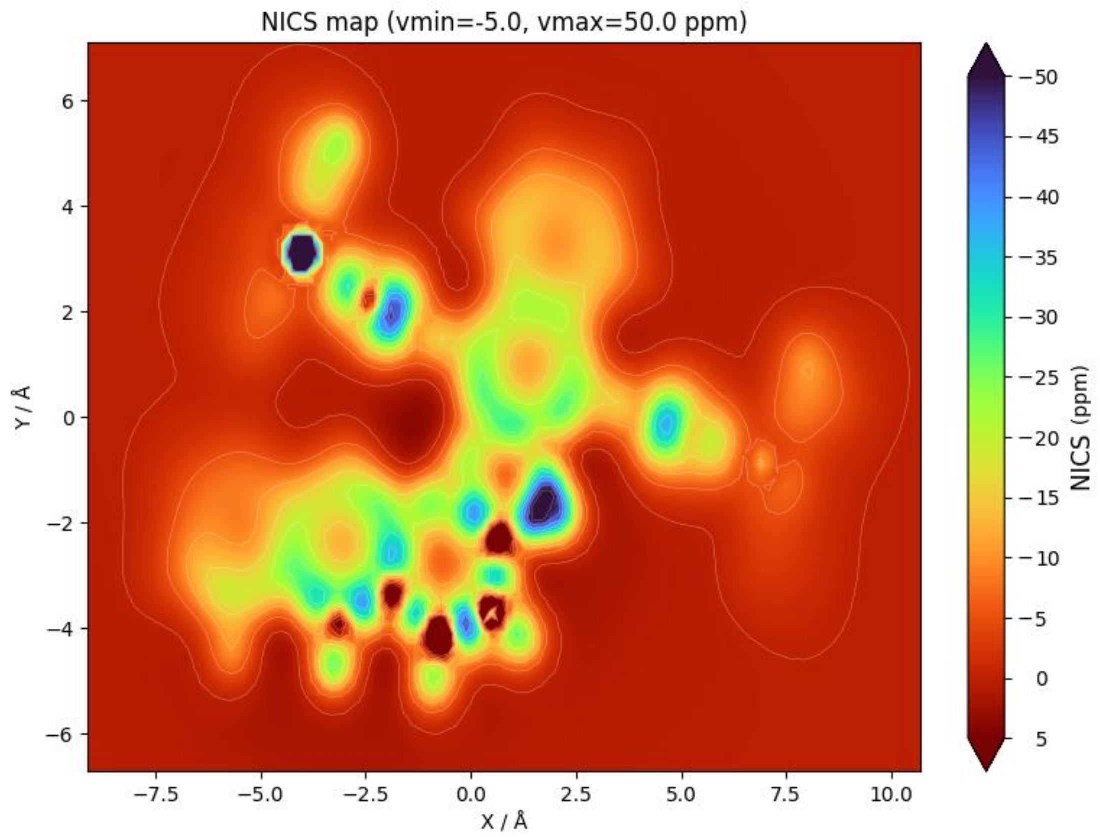

Figure S 39. NICS grid plot 0.53 Å above the molecular plane of **4g**.

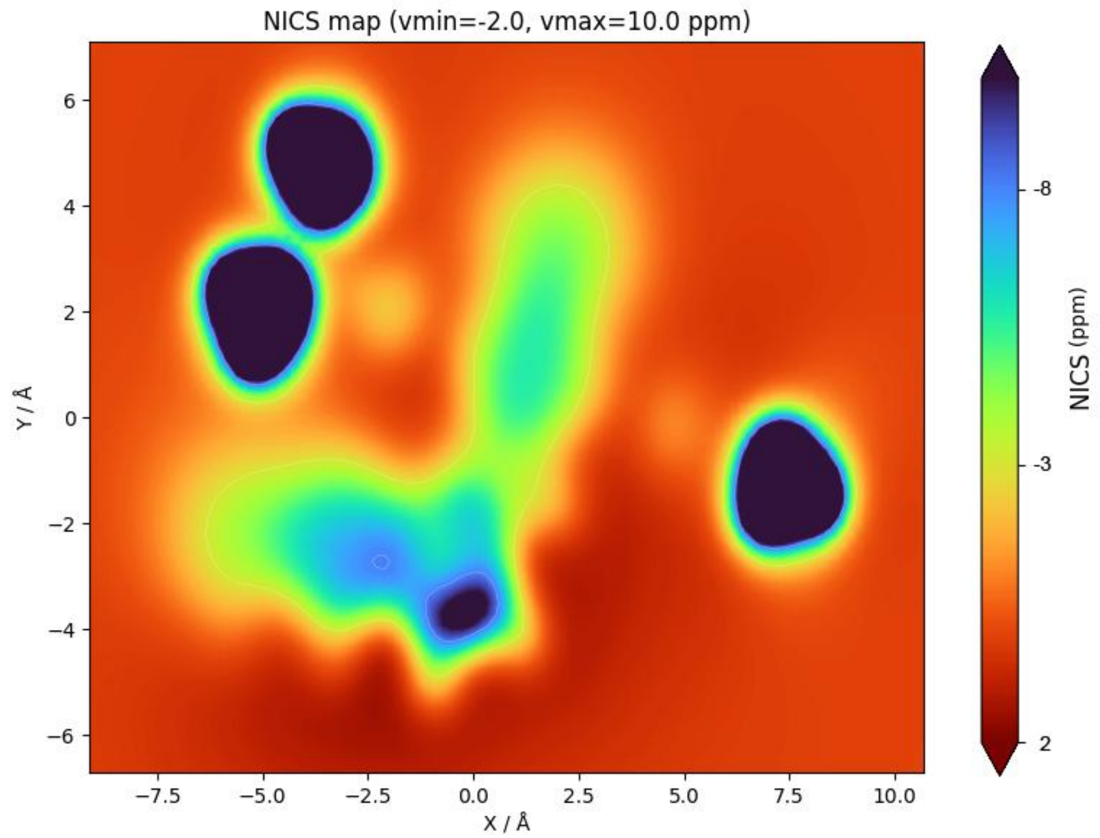

Figure S 40. NICS grid plot 1.7 Å above the molecular plane of **4g**.

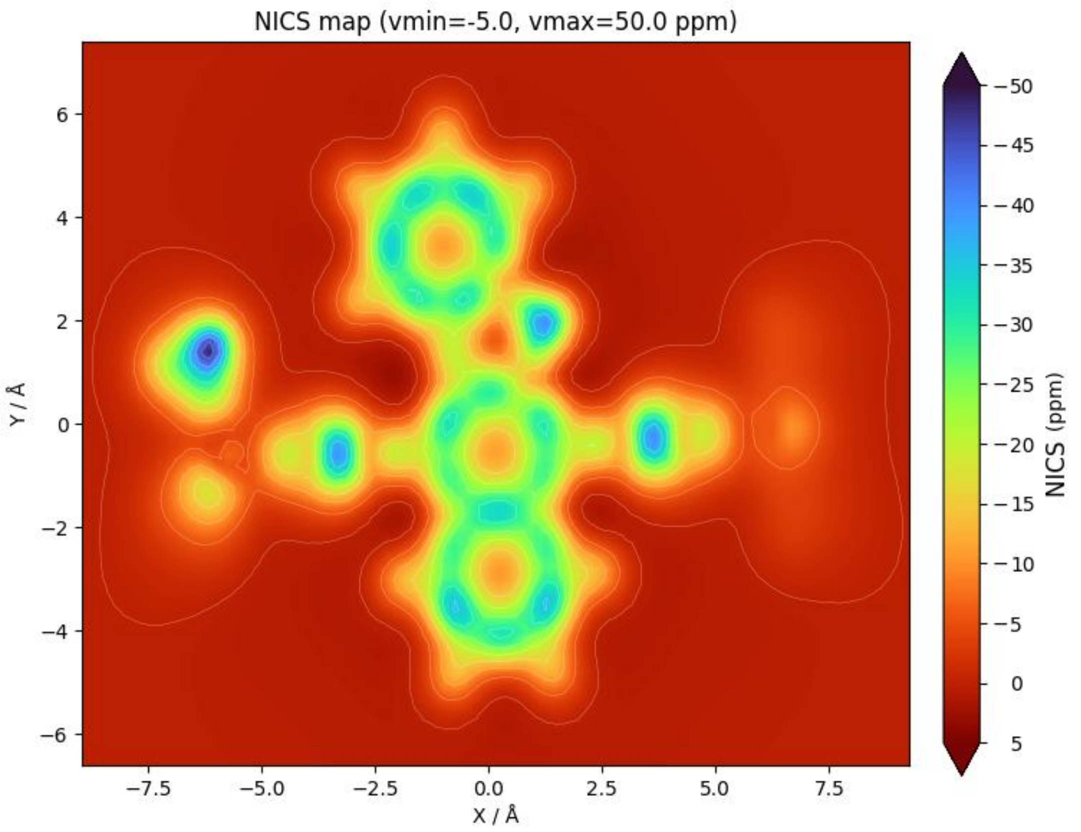

Figure S 41. NICS grid plot 0.53 Å above the molecular plane of **4h**.

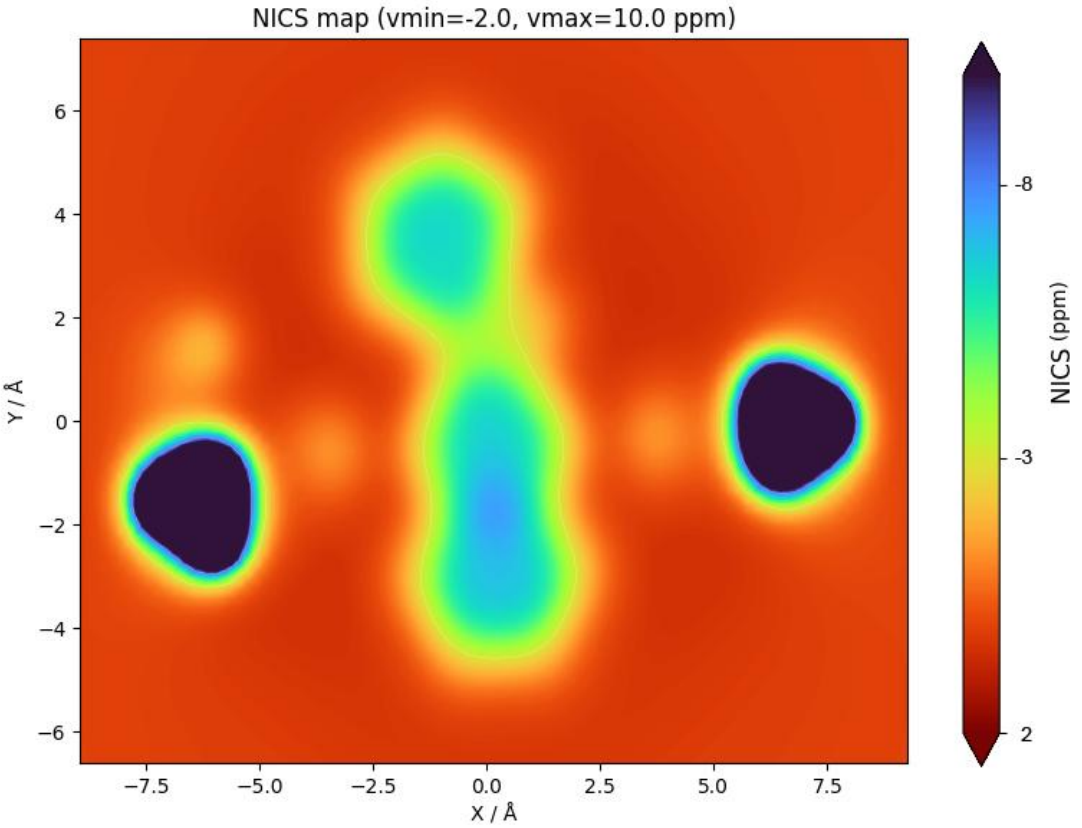

Figure S 42. NICS grid plot 1.7 Å above the molecular plane of **4h**.

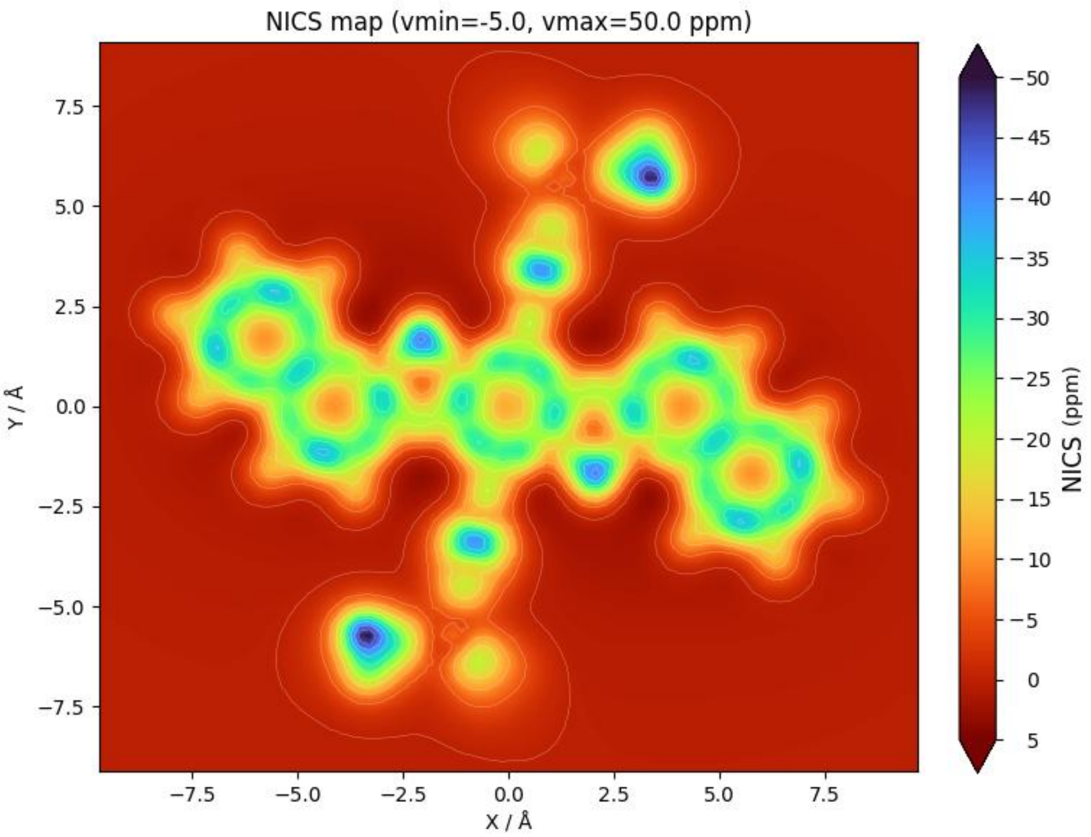

Figure S 43. NICS grid plot 0.53 Å above the molecular plane of **4i**.

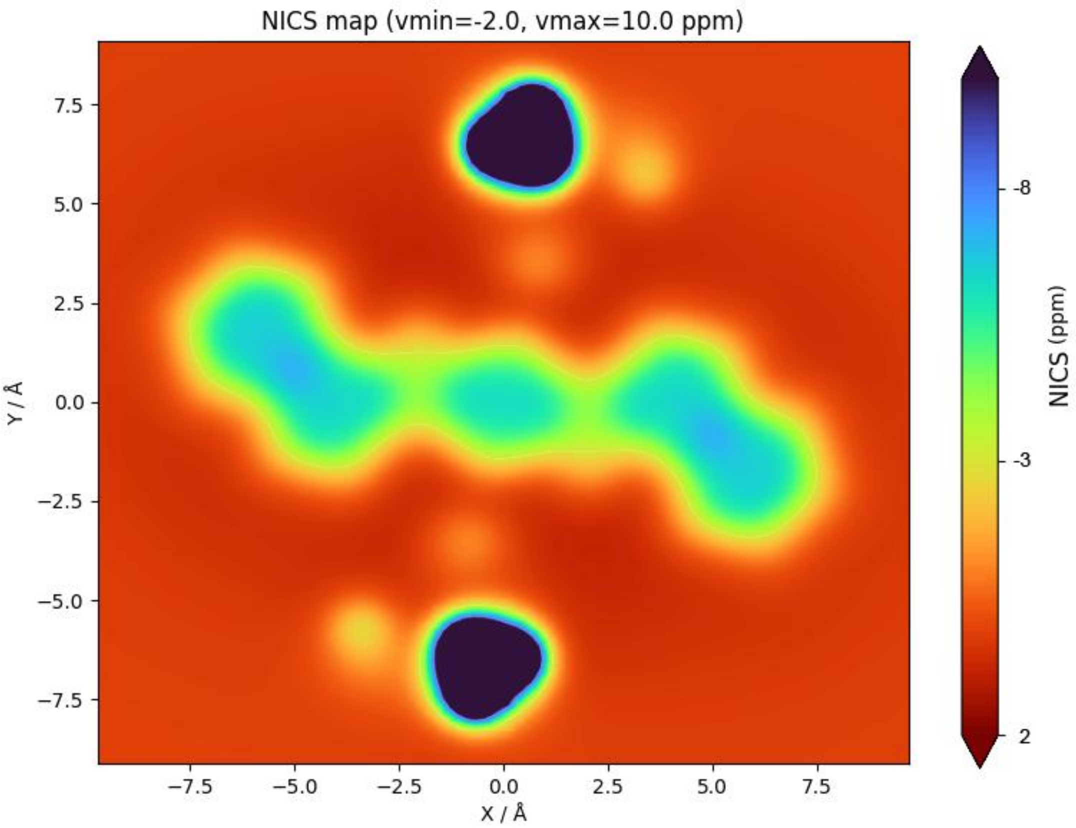

Figure S 44. NICS grid plot 1.7 Å above the molecular plane of **4i**.

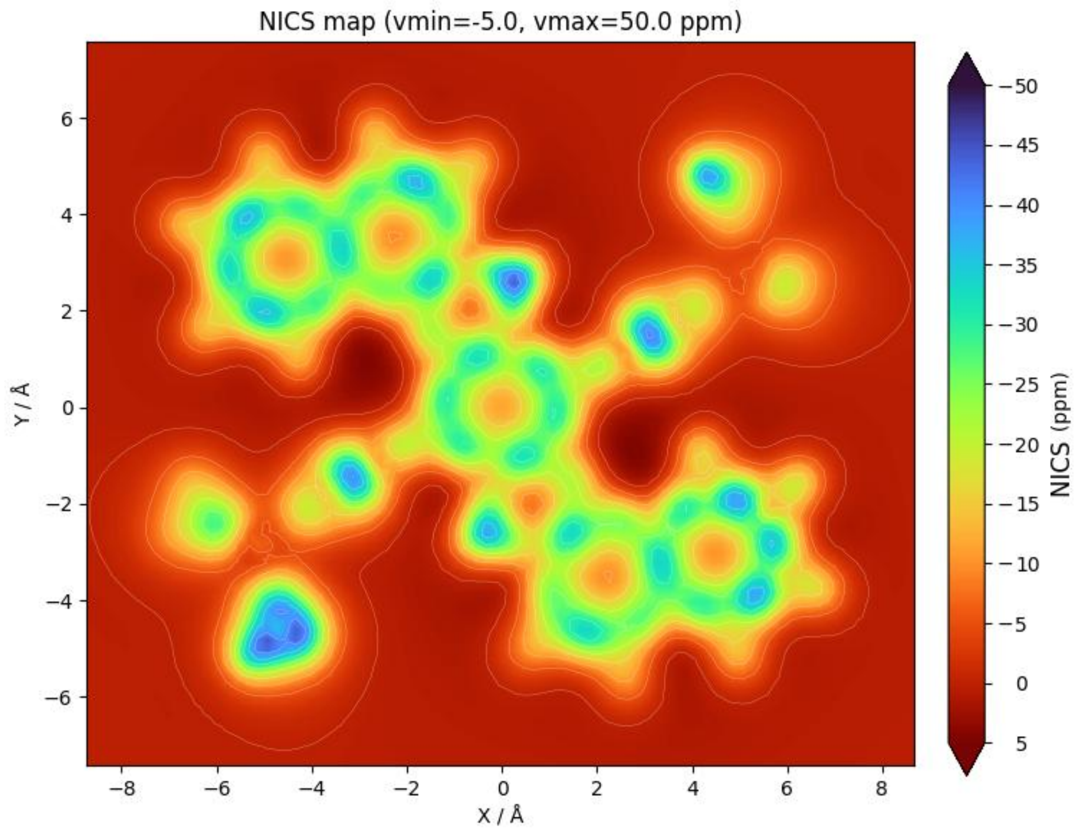

Figure S 45. NICS grid plot 0.53 Å above the molecular plane of **4j**.

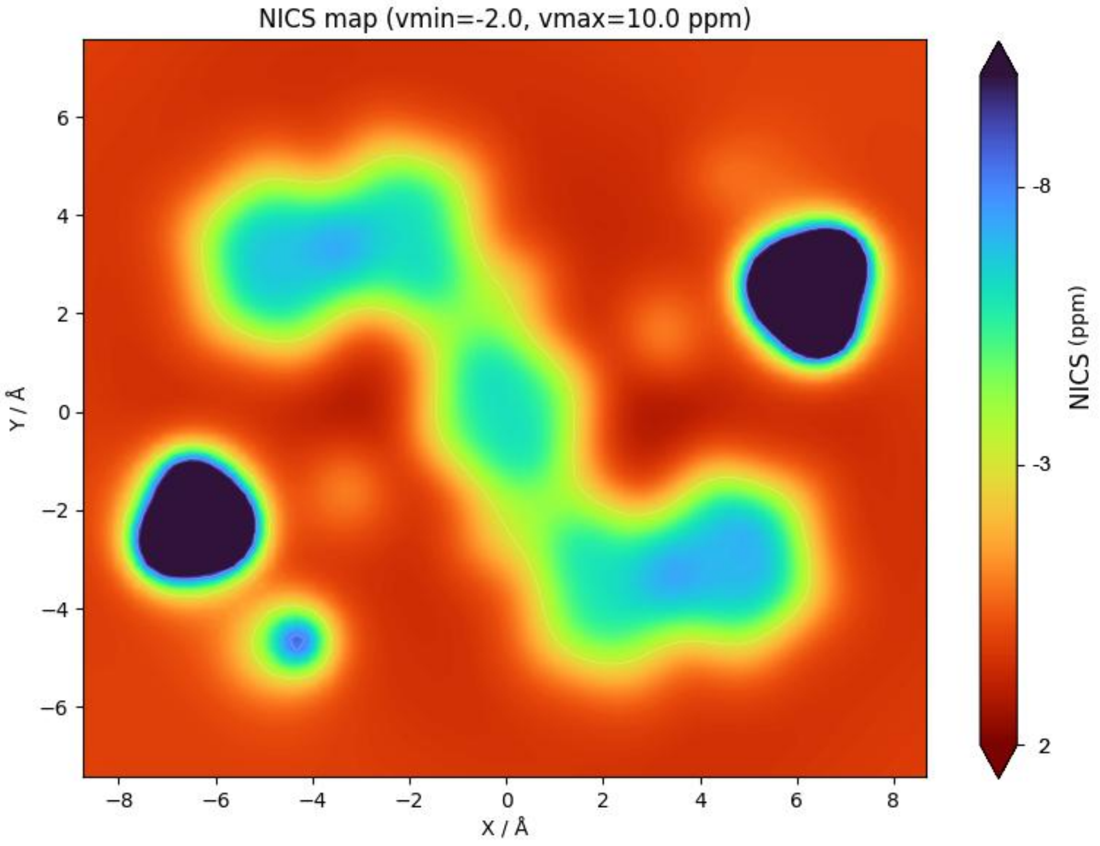

Figure S 46. NICS grid plot 1.7 Å above the molecular plane of **4j**.

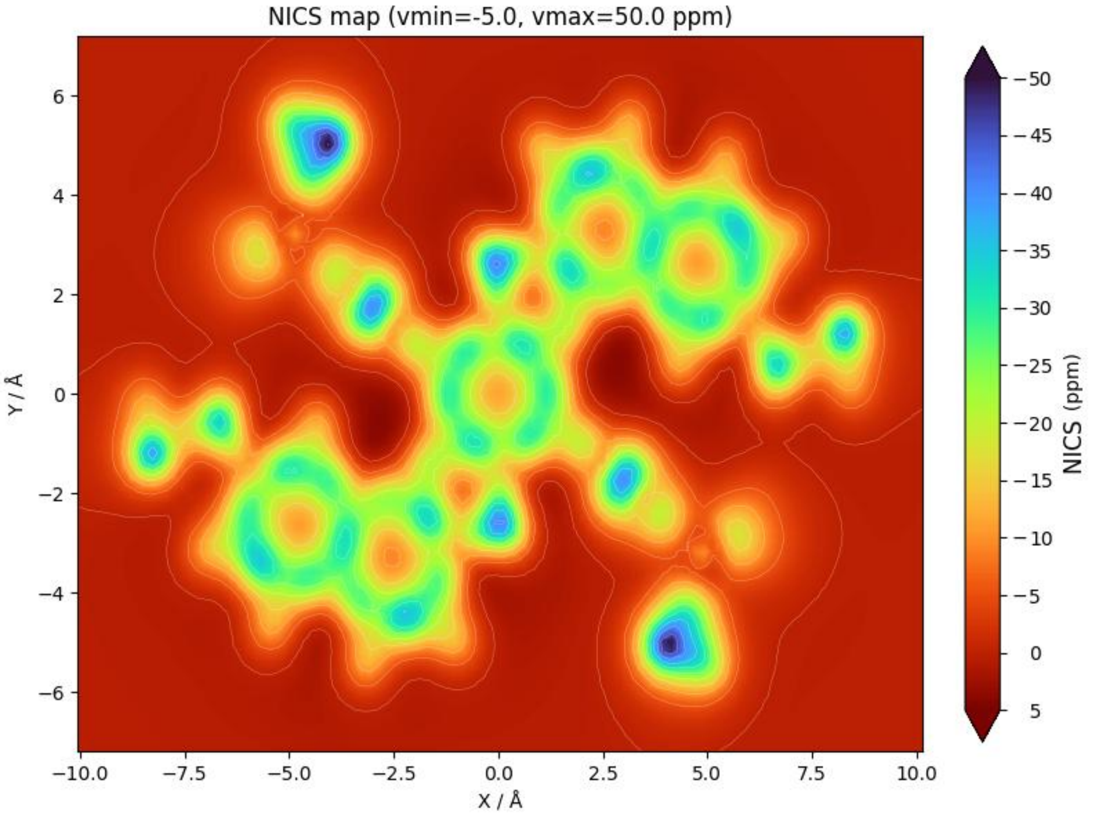

Figure S 47. NICS grid plot 0.53 Å above the molecular plane of **4k**.

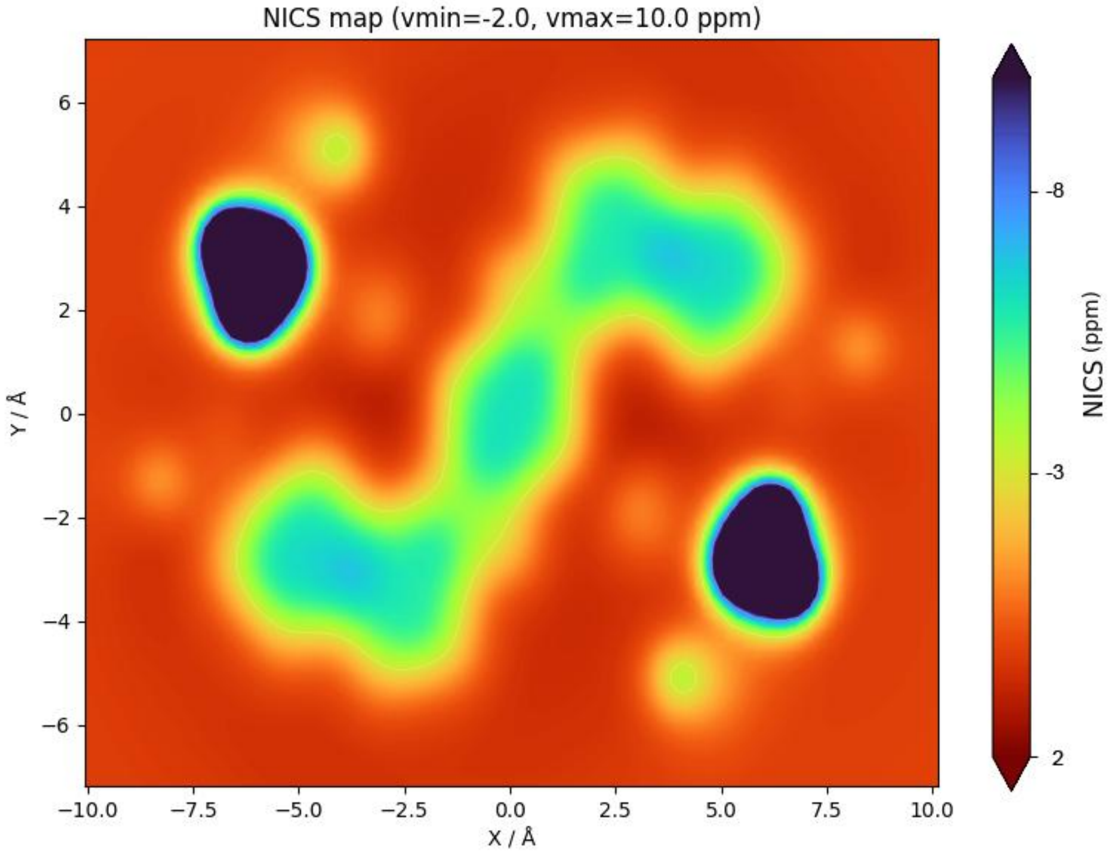

Figure S 48. NICS grid plot 1.7 Å above the molecular plane of 4k.

S 10. ^{1}H and ^{13}C{^{1}H} NMR Spectra

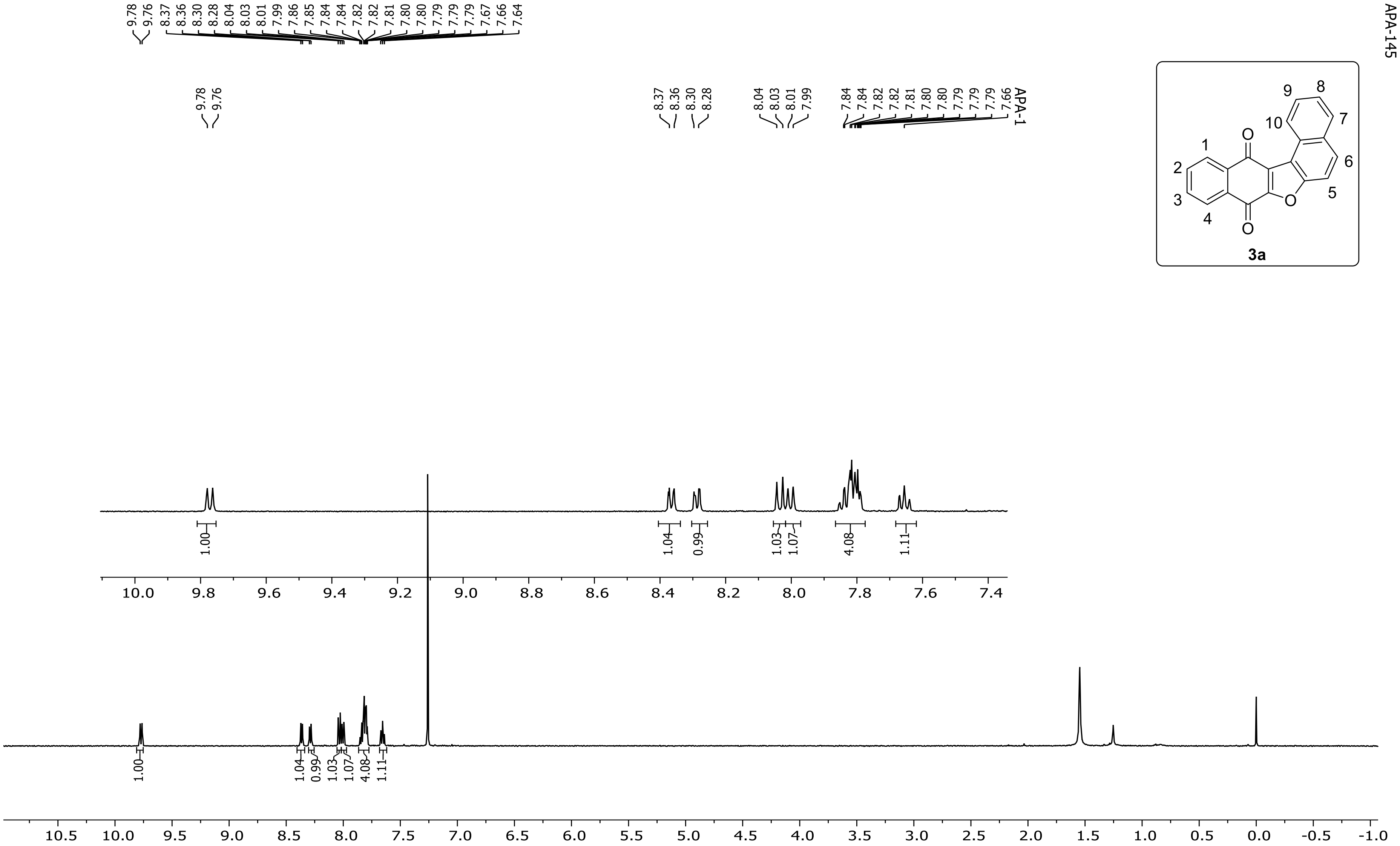

Figure S 49. ^{1}H NMR (top) spectra of **3a** in $CDCl_3$ at 500 MHz.

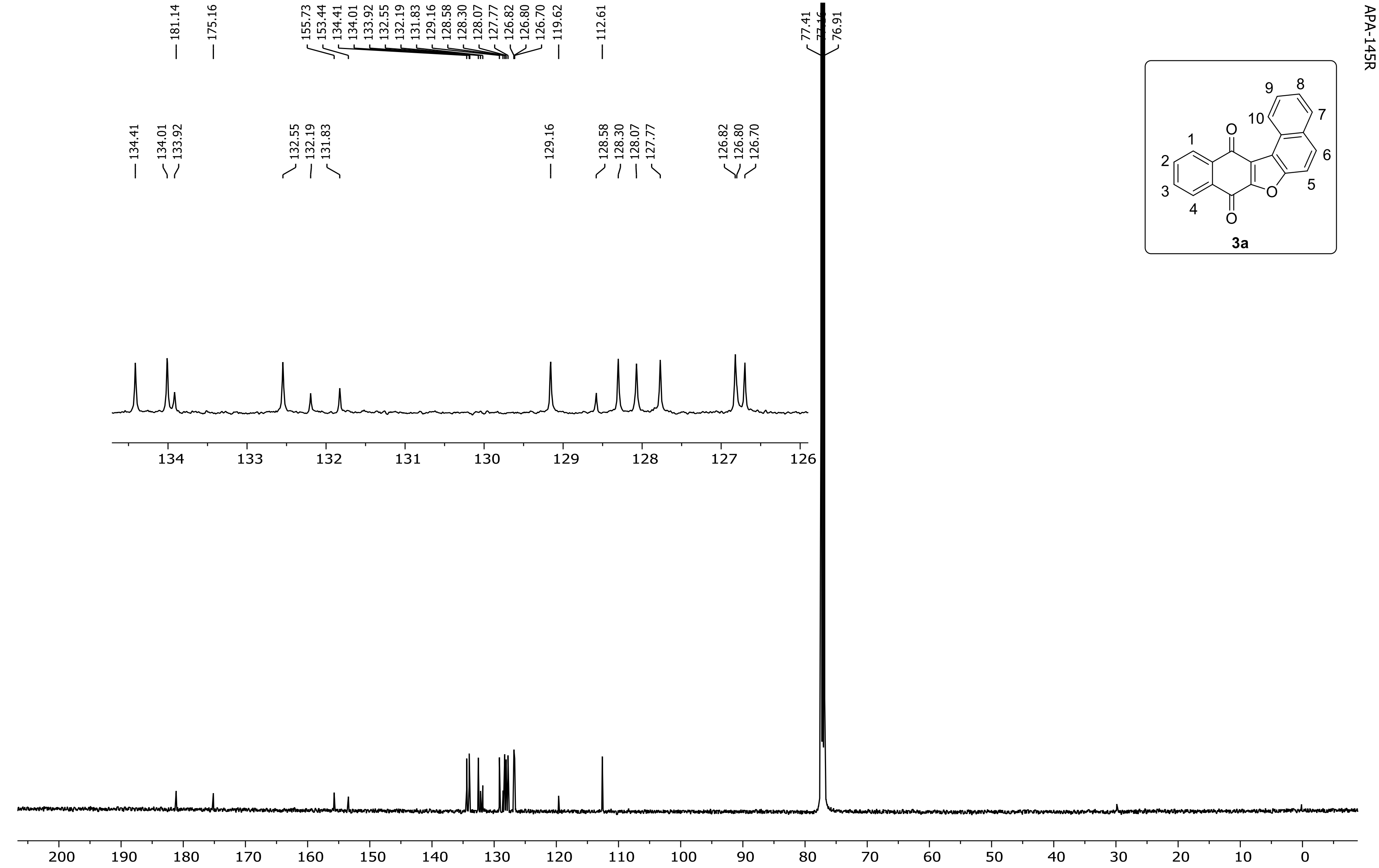

Figure S 50. $^{13}C\{^{1}H\}$ NMR (top) spectra of **3a** in $CDCl_3$ at 126 MHz.

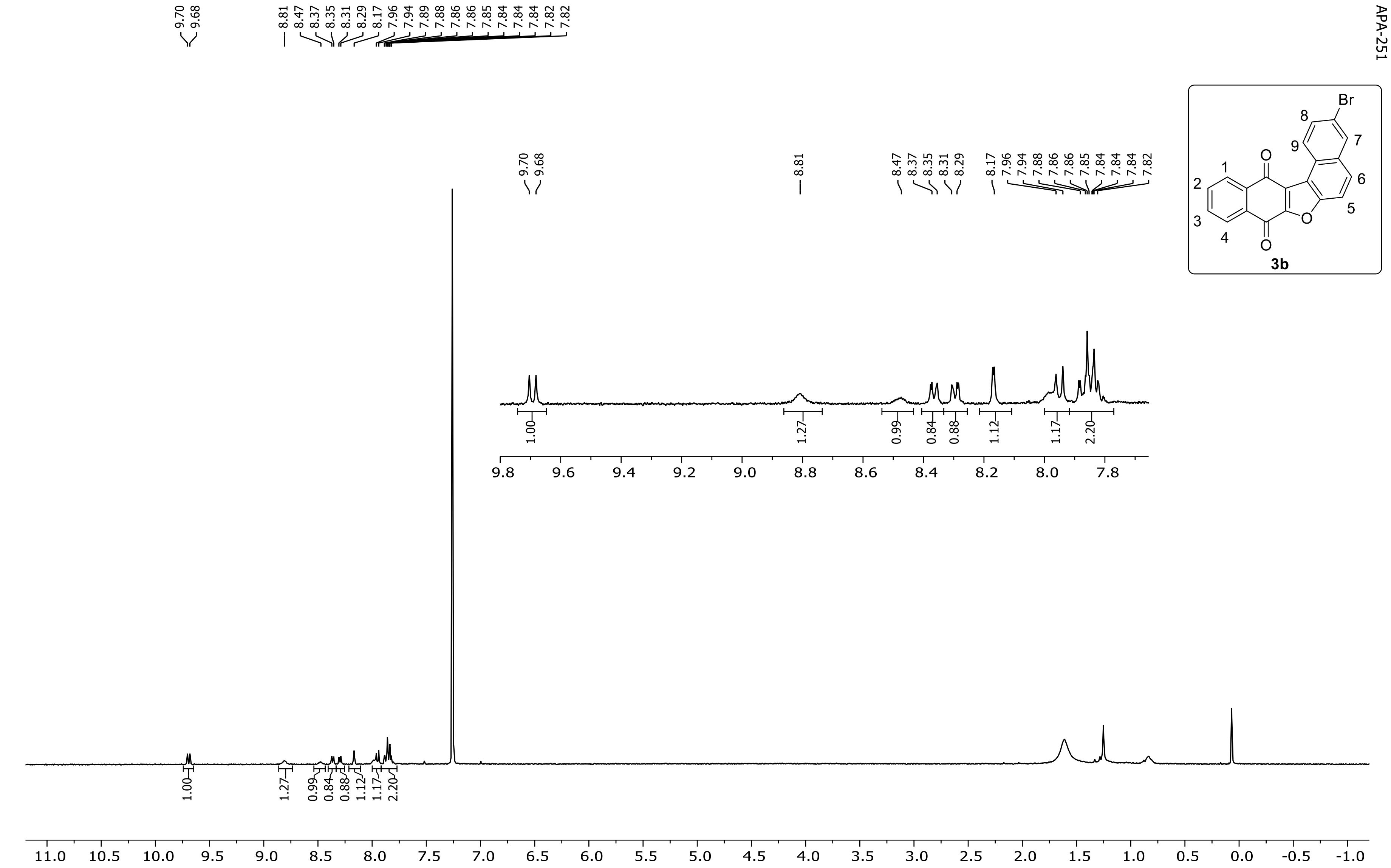

Figure S 51. ^{1}H NMR (top) spectra of **3b** in $CDCl_3$ at 400 MHz..

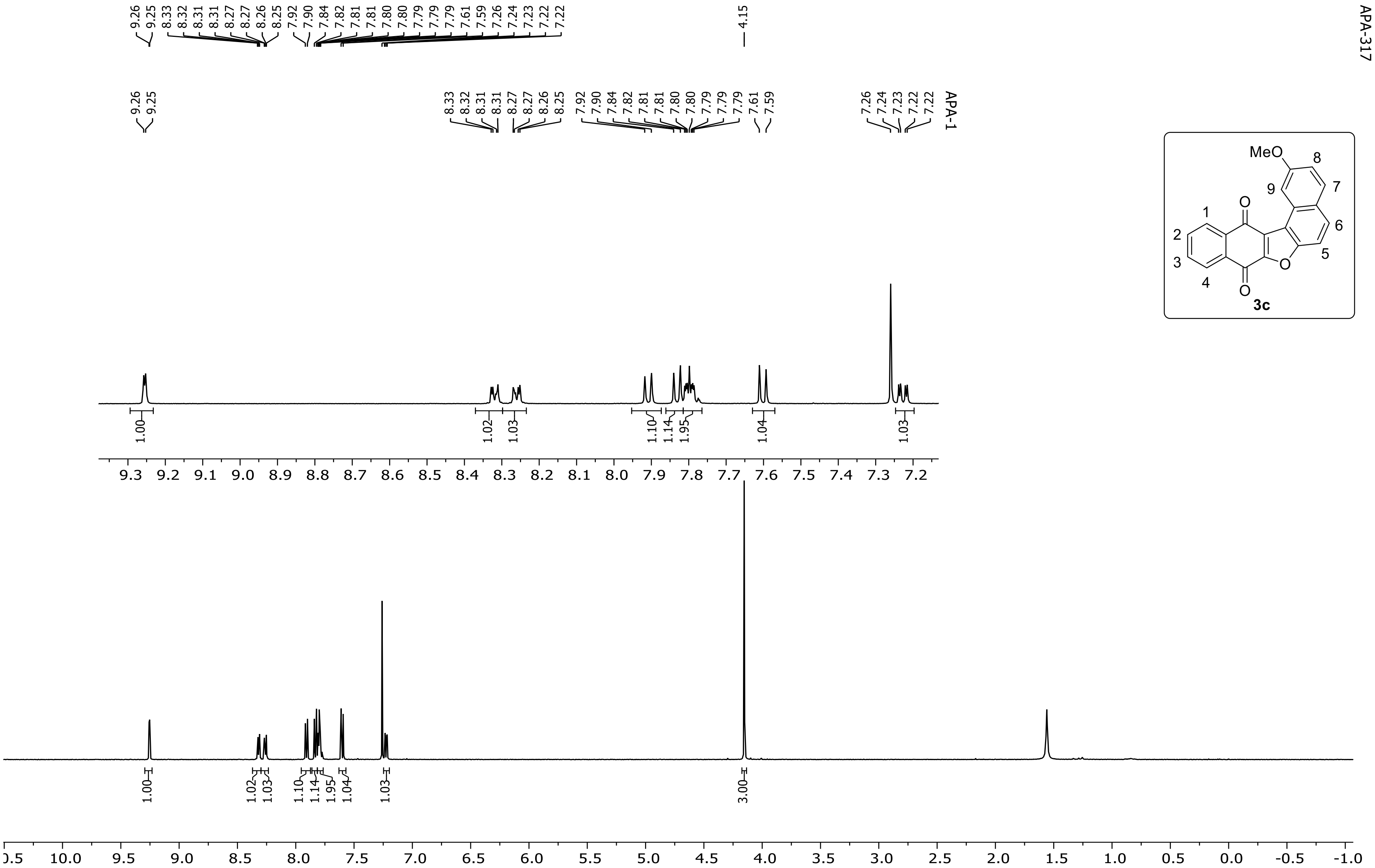

Figure S 52. ^{1}H NMR (top) spectra of **3c** in $CDCl_3$ at 500 MHz..

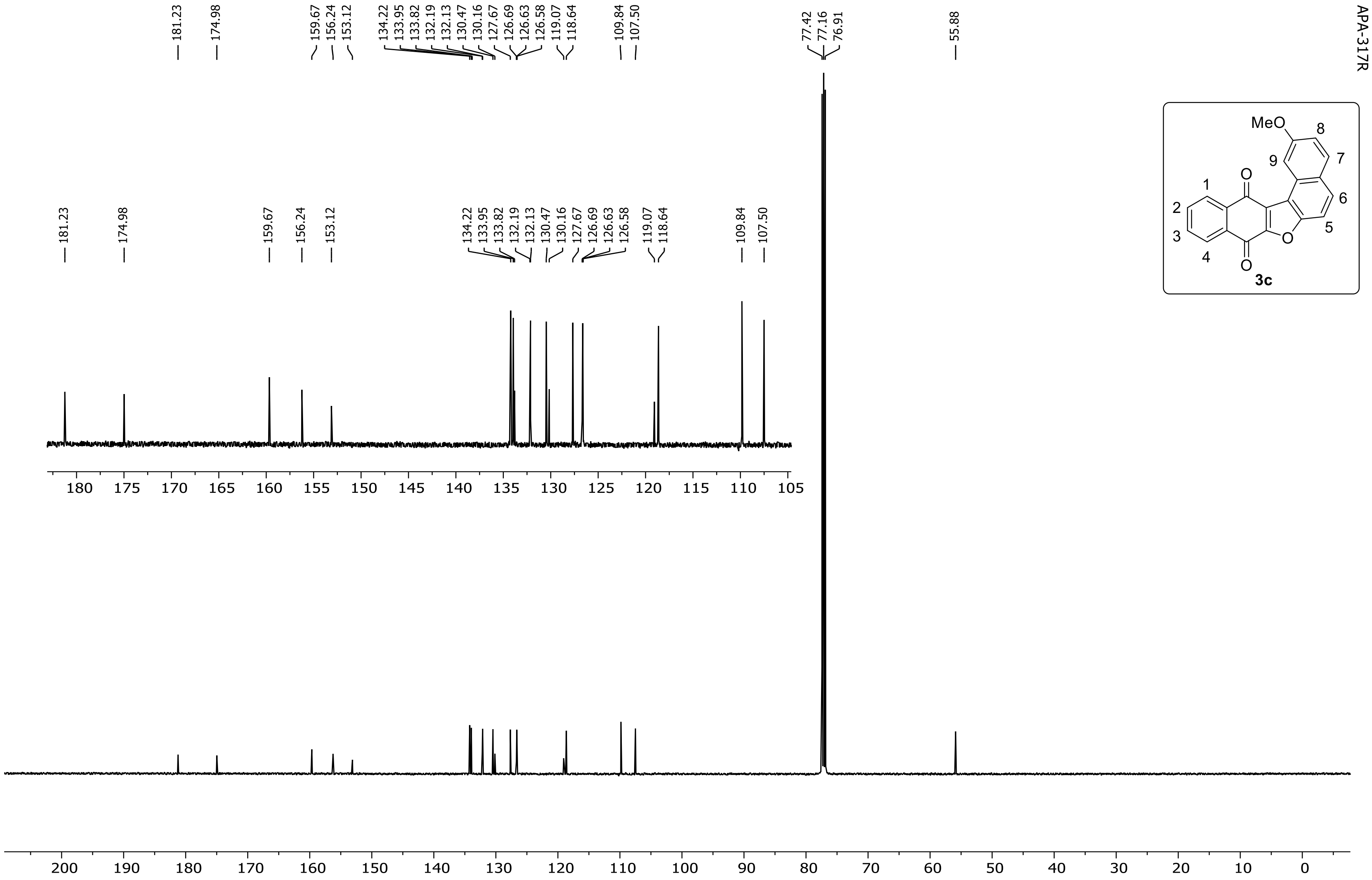

Figure S 53. $^{13}C\{^{1}H\}$ NMR (top) spectra of **3c** in $CDCl_3$ at 126 MHz..

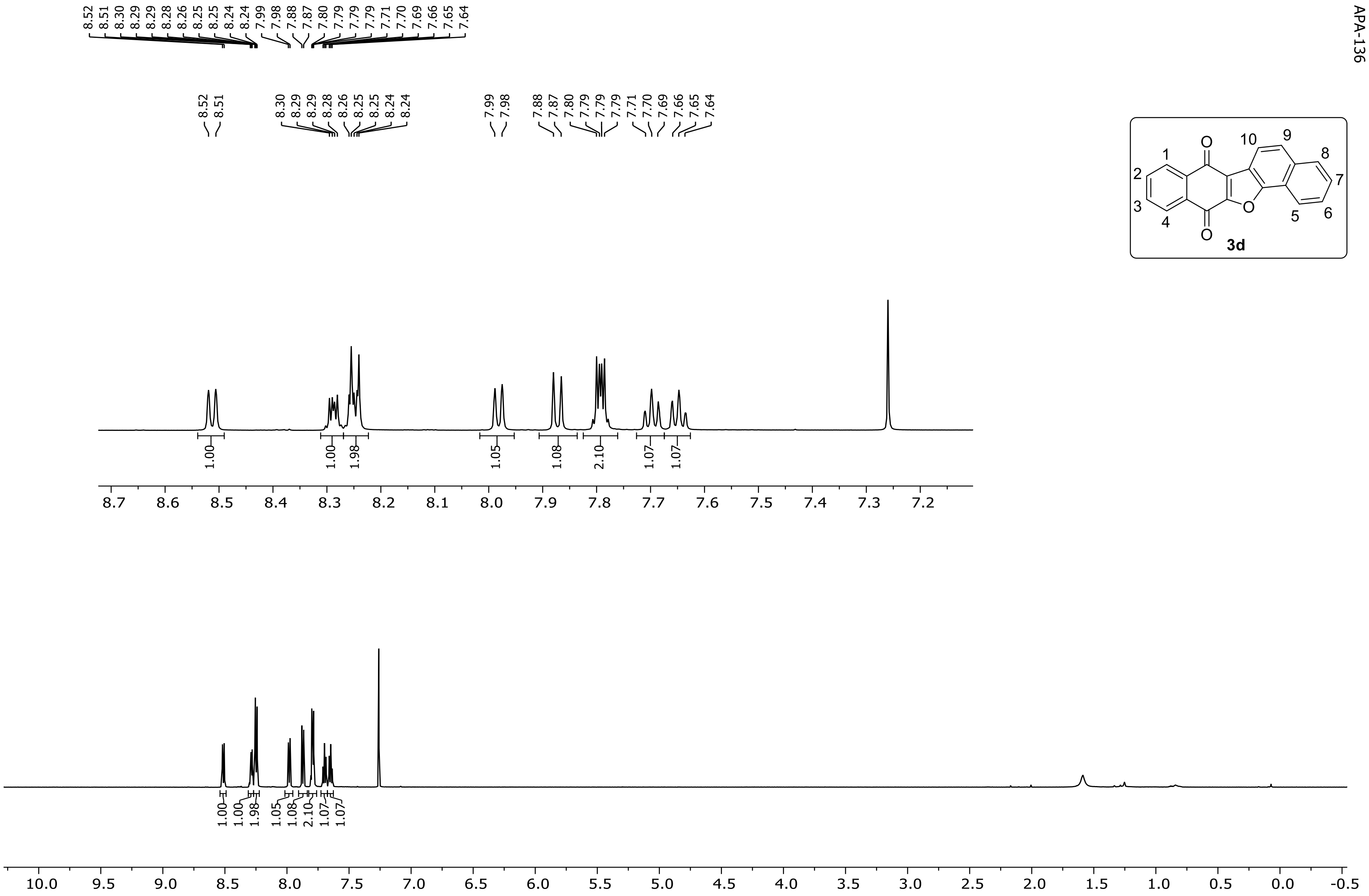

Figure S 54. ^{1}H NMR (top) spectra of **3d** in $CDCl_3$ at 600 MHz..

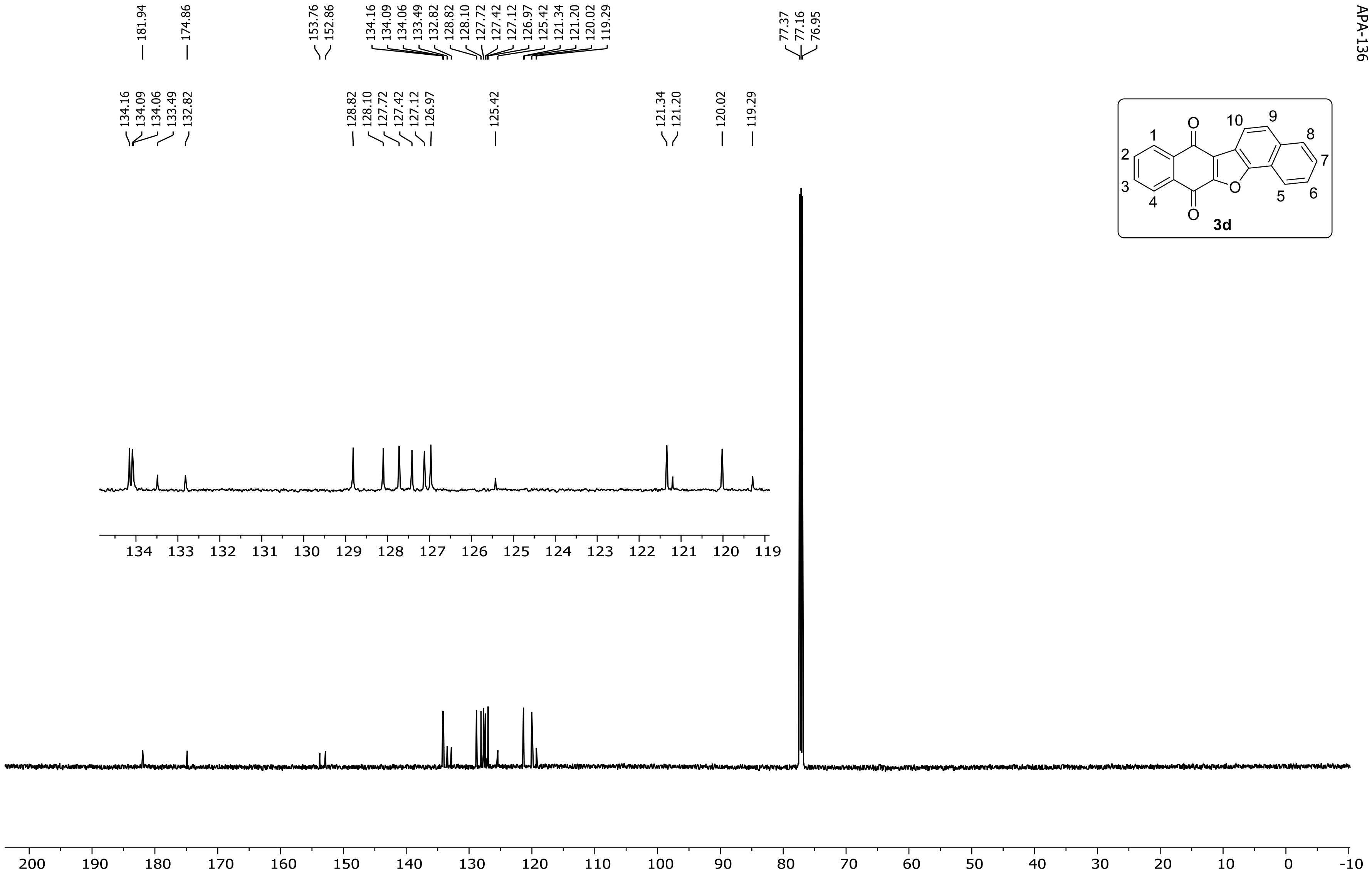

Figure S 55. $^{13}C\{^{1}H\}$ NMR (top) spectra of **3d** in $CDCl_3$ at 151 MHz.

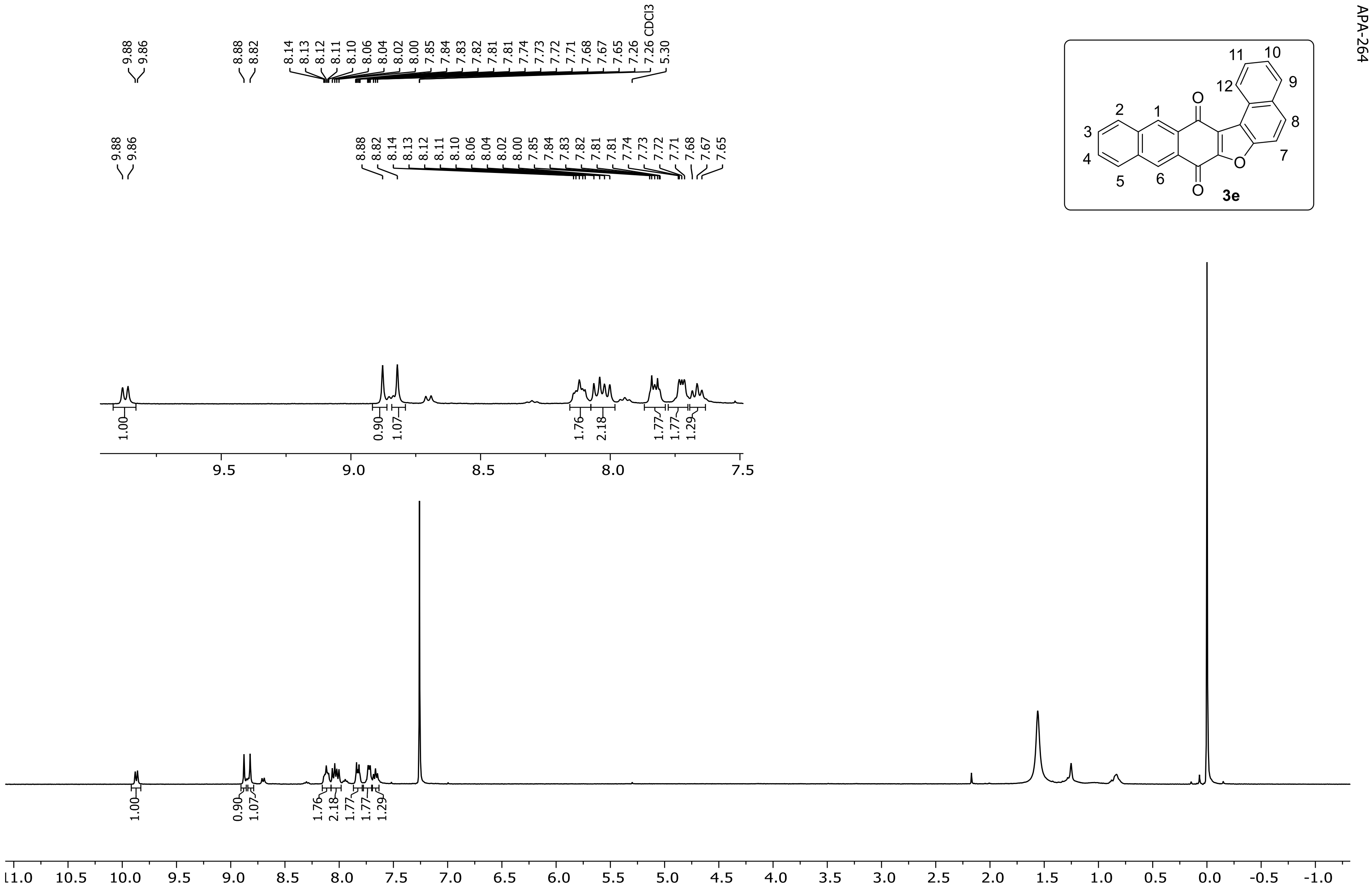

Figure S 56. ^{1}H NMR (top) spectra of **3e** in $CDCl_3$ at 400 MHz.

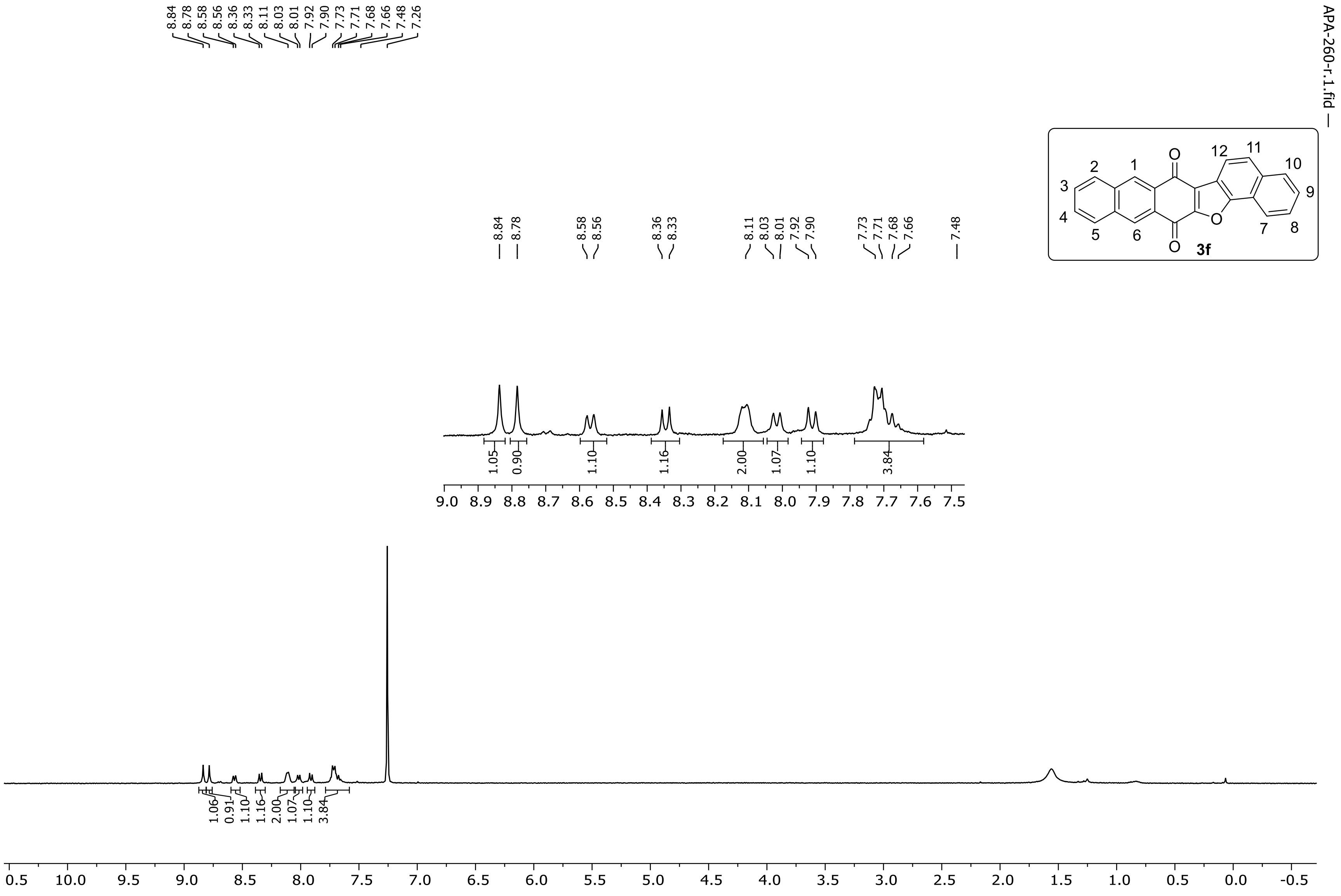

Figure S 57. ^{1}H NMR (top) spectra of **3f** in $CDCl_3$ at 400 MHz...

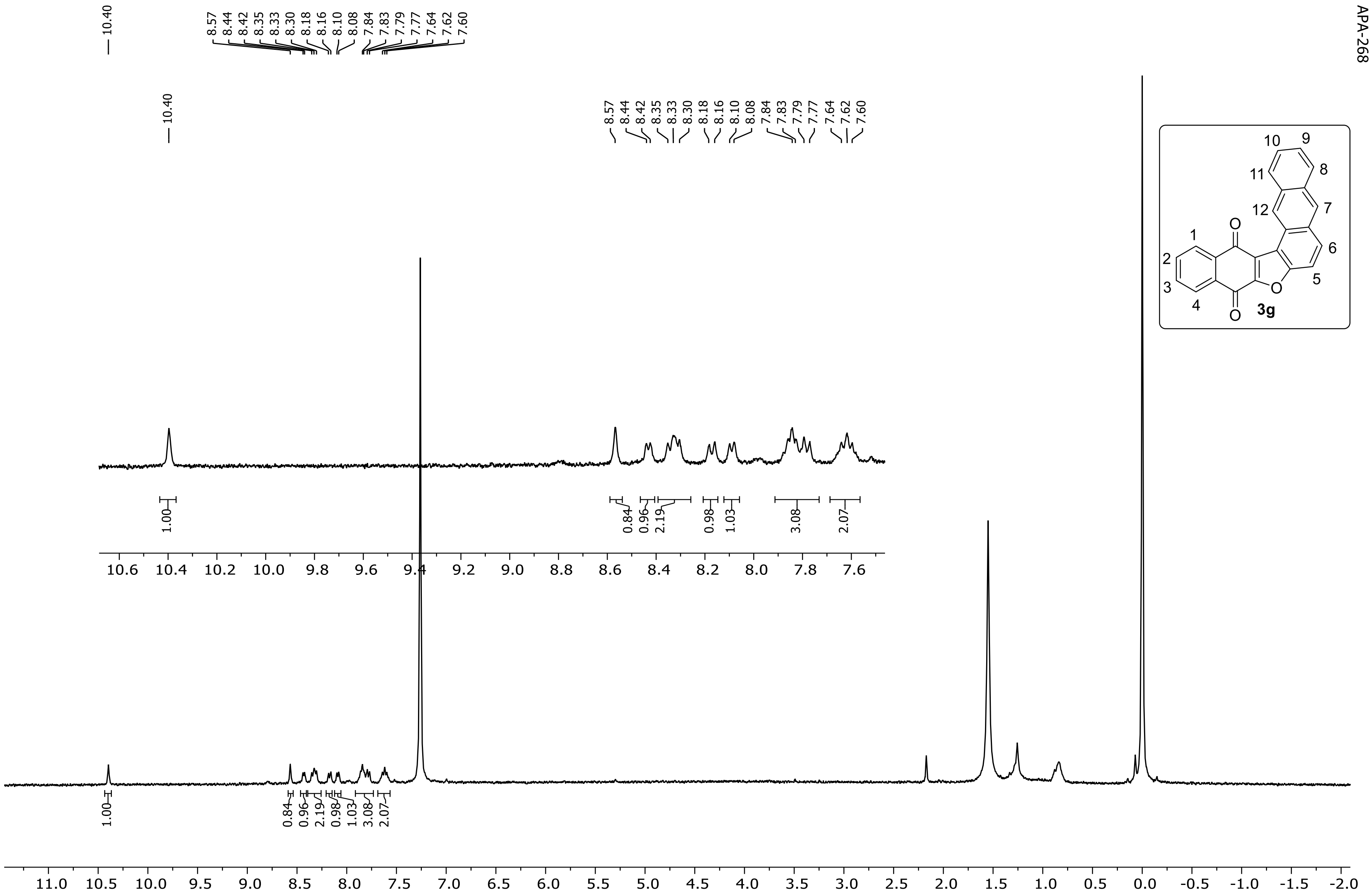

Figure S 58. ^{1}H NMR (top) spectra of **3g** in $CDCl_3$ at 400 MHz...

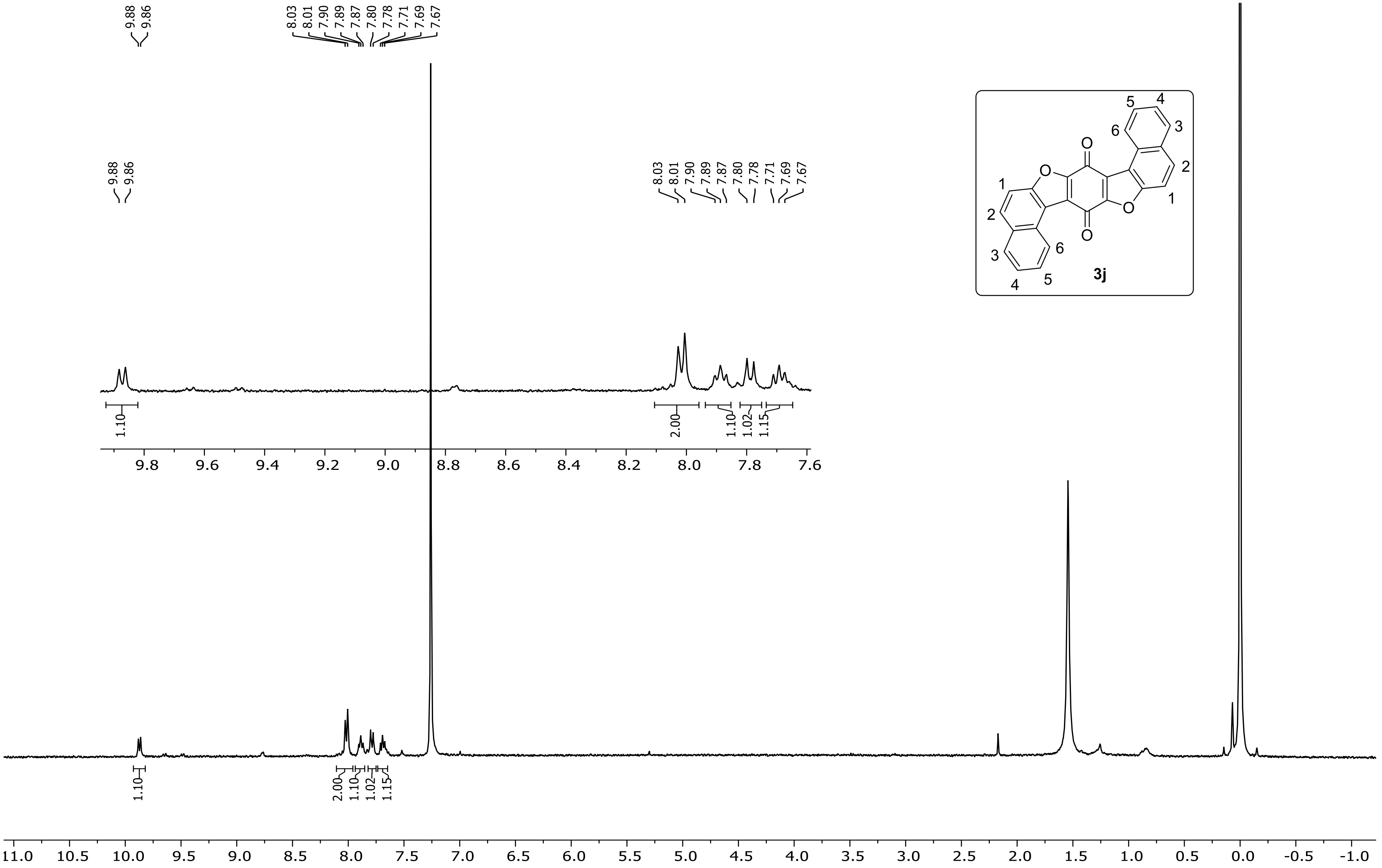

Figure S 59. ^{1}H NMR (top) spectra of **3j** in $CDCl_3$ at 400 MHz.

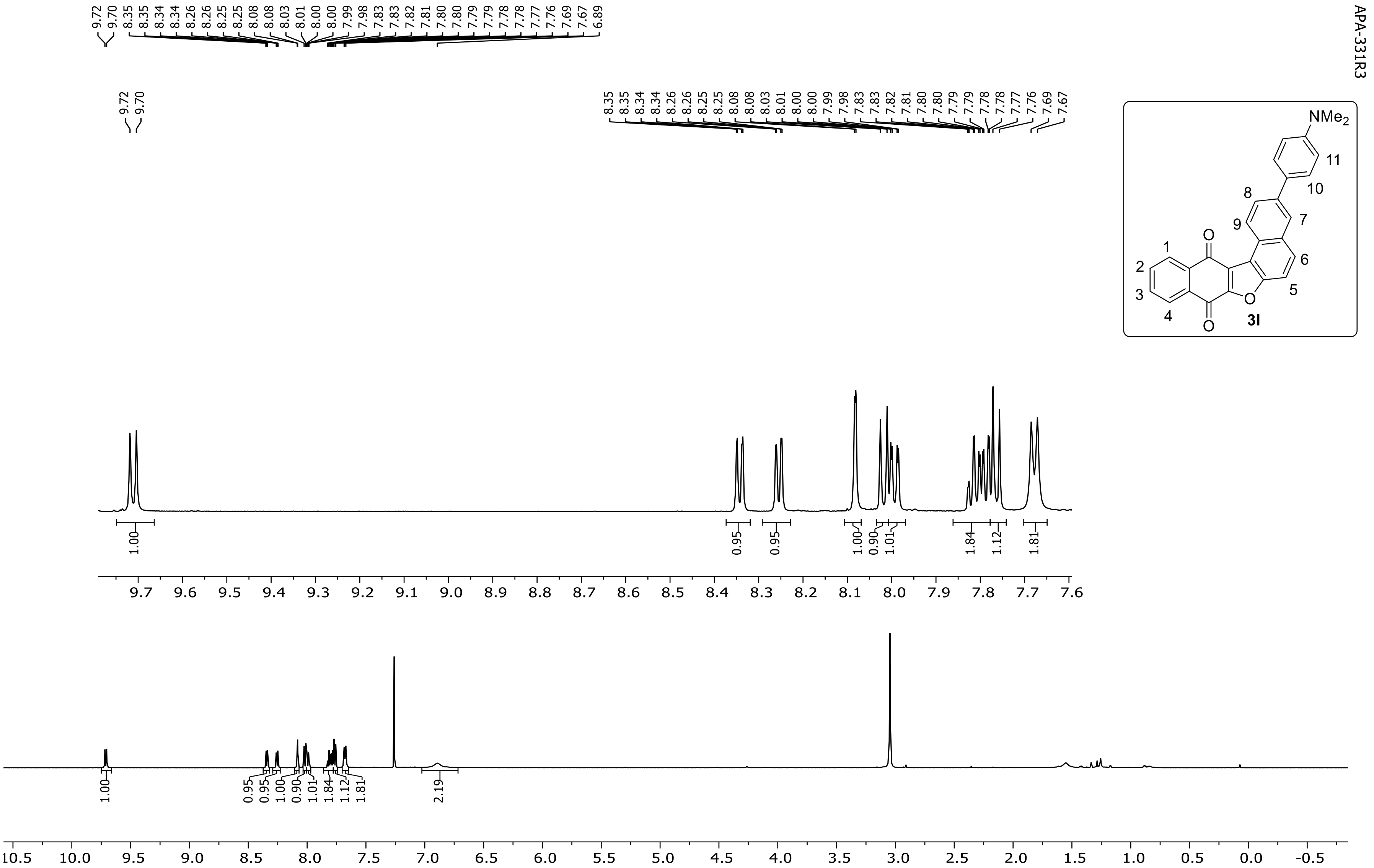

Figure S 60. ^{1}H NMR (top) spectra of **3l** in $CDCl_3$ at 600 MHz.

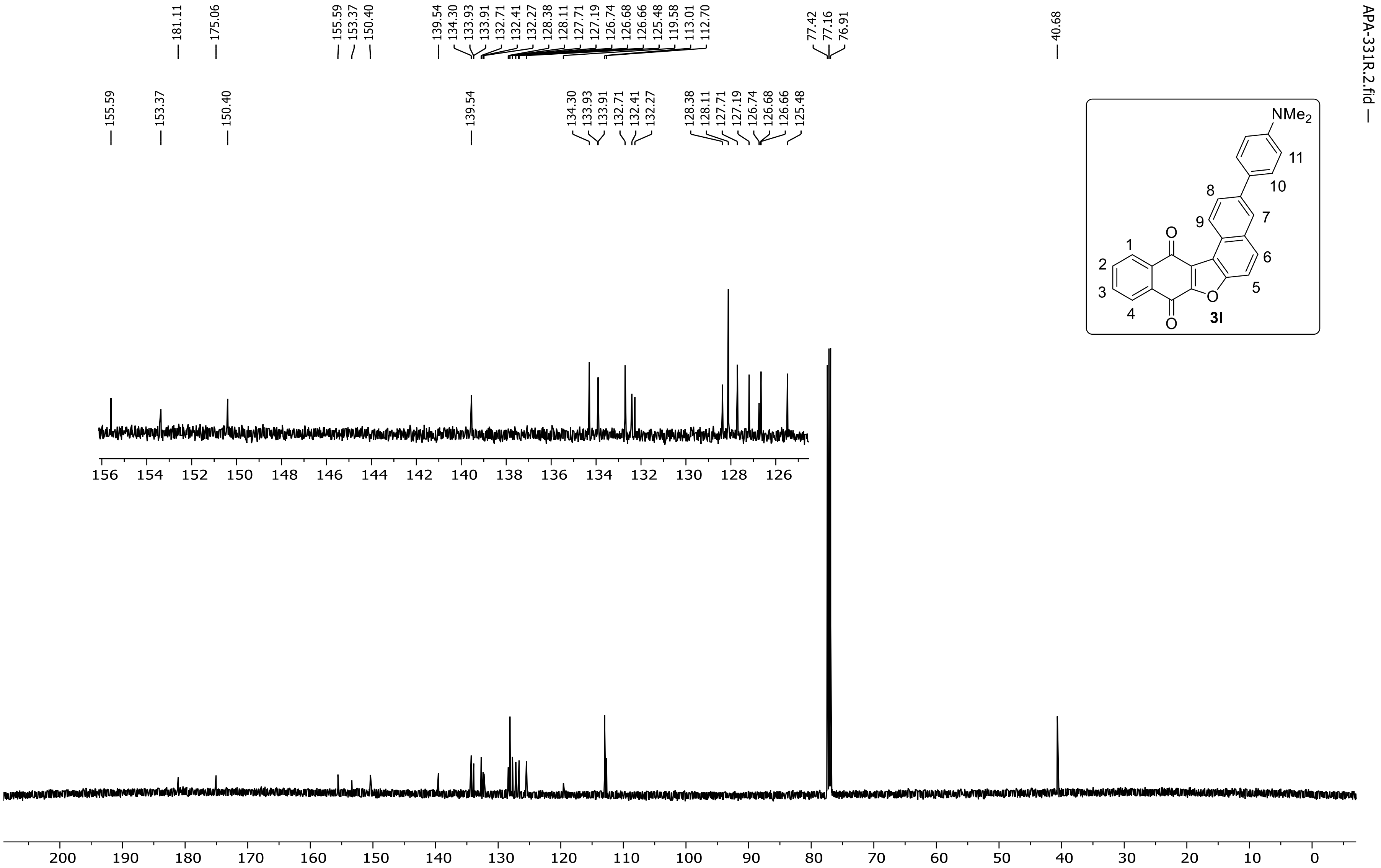

Figure S 61. $^{13}C\{^{1}H\}$ NMR (top) spectra of **3l** in $CDCl_3$ at 126 MHz.

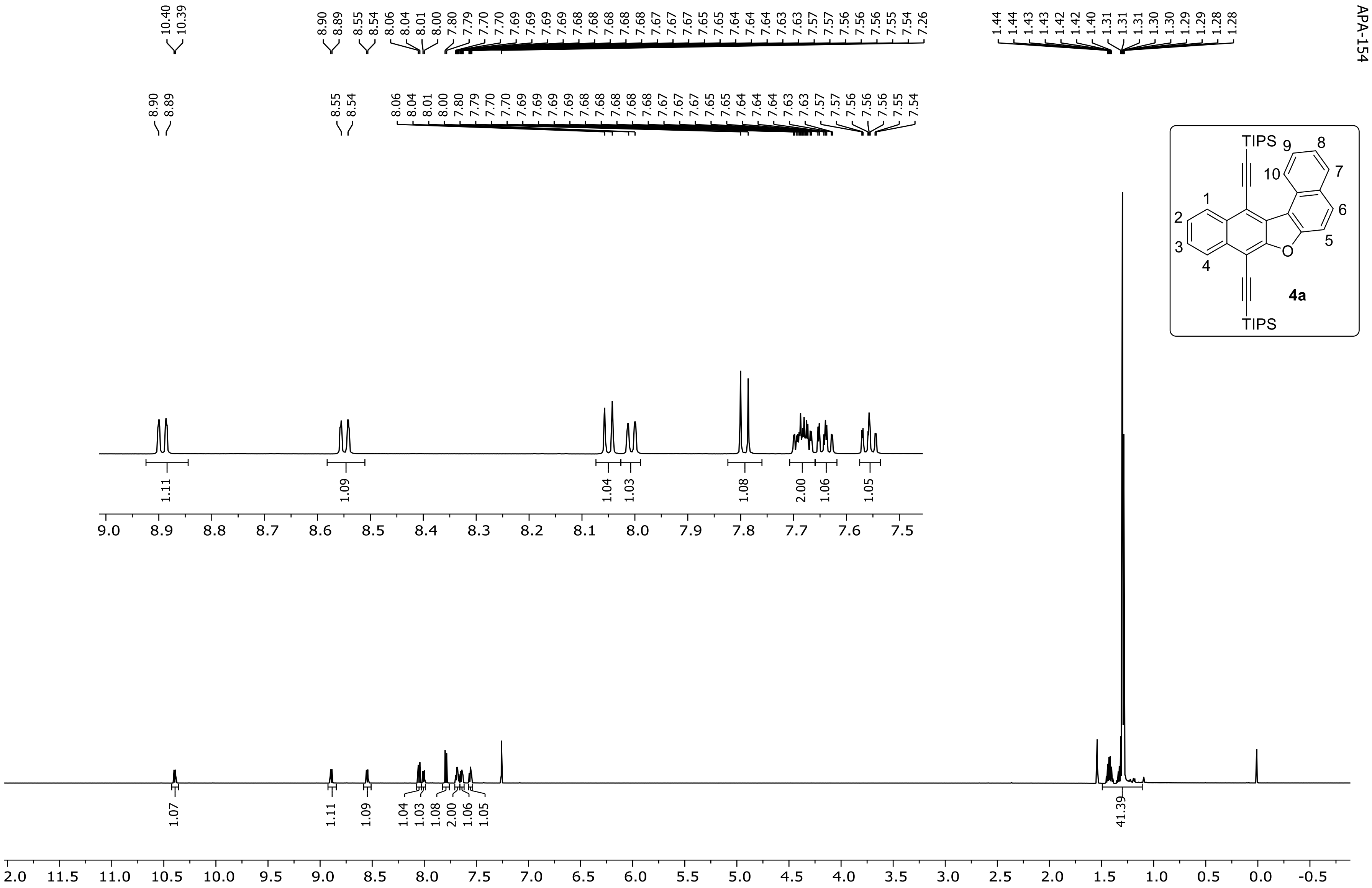

Figure S 62. ^{1}H NMR (top) spectra of **4a** in $CDCl_3$ at 600 MHz.

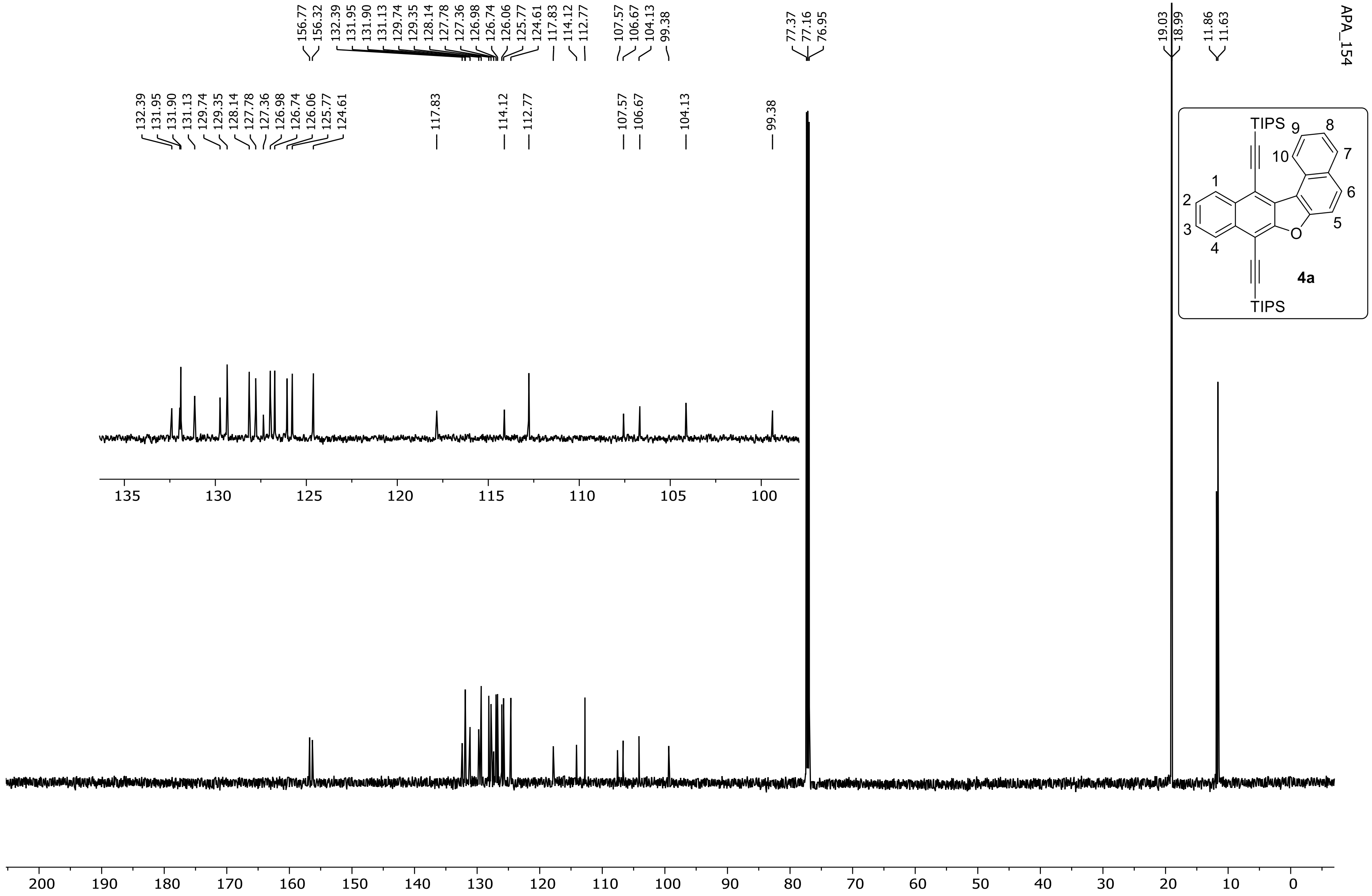

Figure S 63. $^{13}C\{^{1}H\}$ NMR (top) spectra of **4a** in $CDCl_3$ at 151 MHz.

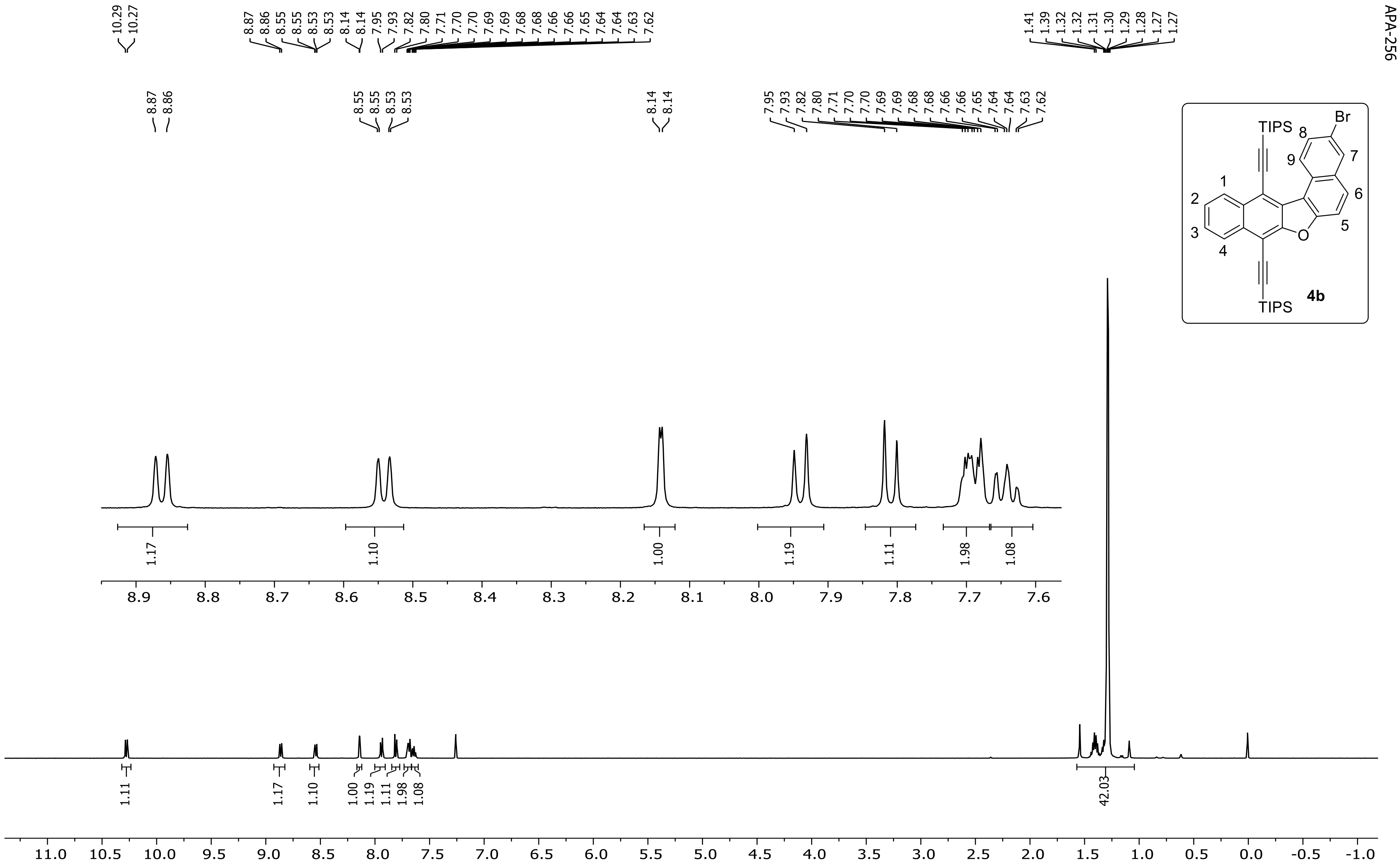

Figure S 64. ^{1}H NMR (top) spectra of **4b** in $CDCl_3$ at 500 MHz.

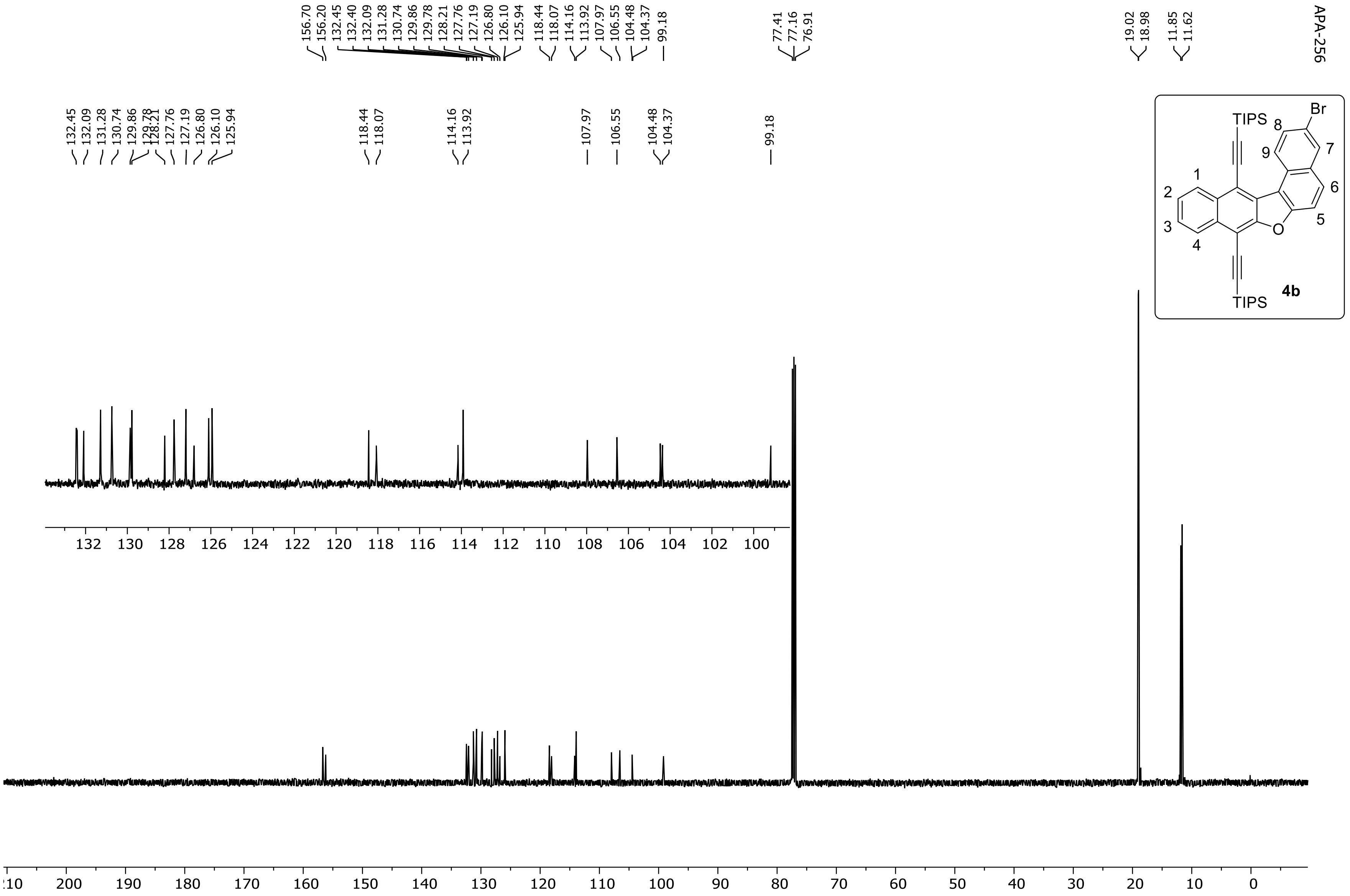

Figure S 65. $^{13}C\{^1H\}$ NMR (top) spectra of **4b** in $CDCl_3$ at 126 MHz.

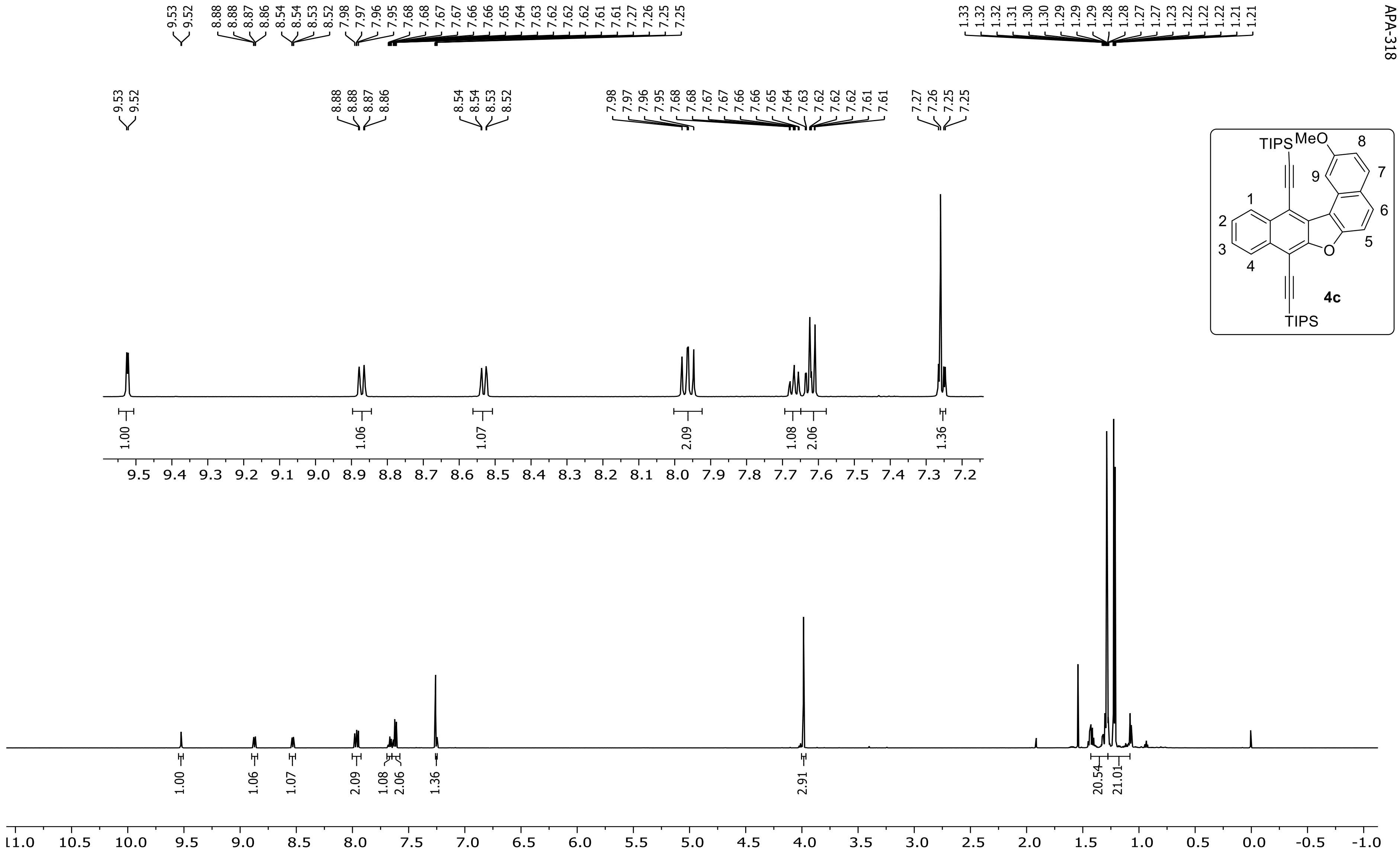

Figure S 66. ^{1}H NMR (top) spectra of **4c** in $CDCl_3$ at 600 MHz.

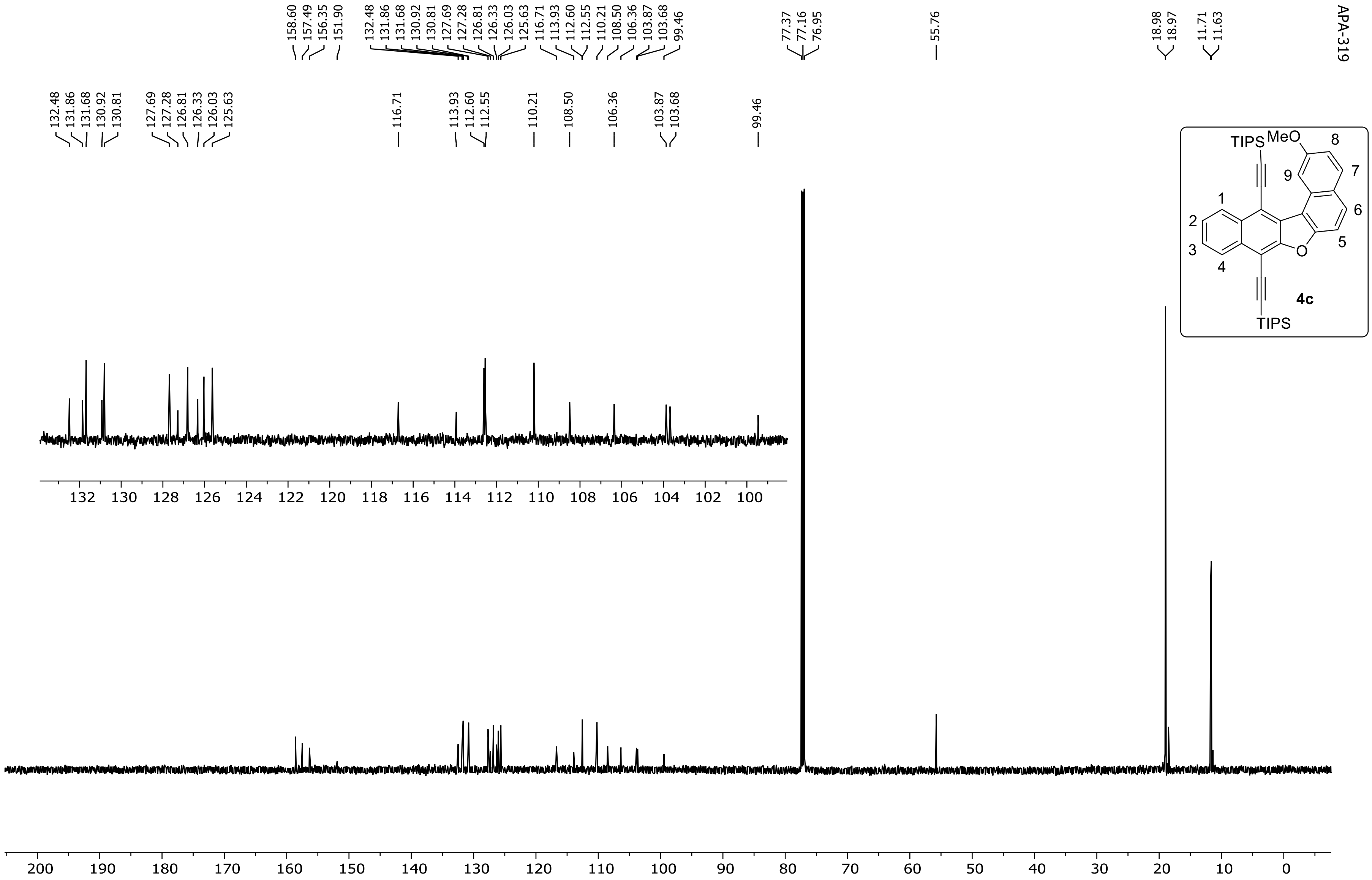

Figure S 67. $^{13}C\{^1H\}$ NMR (top) spectra of **4c** in $CDCl_3$ at 151 MHz.

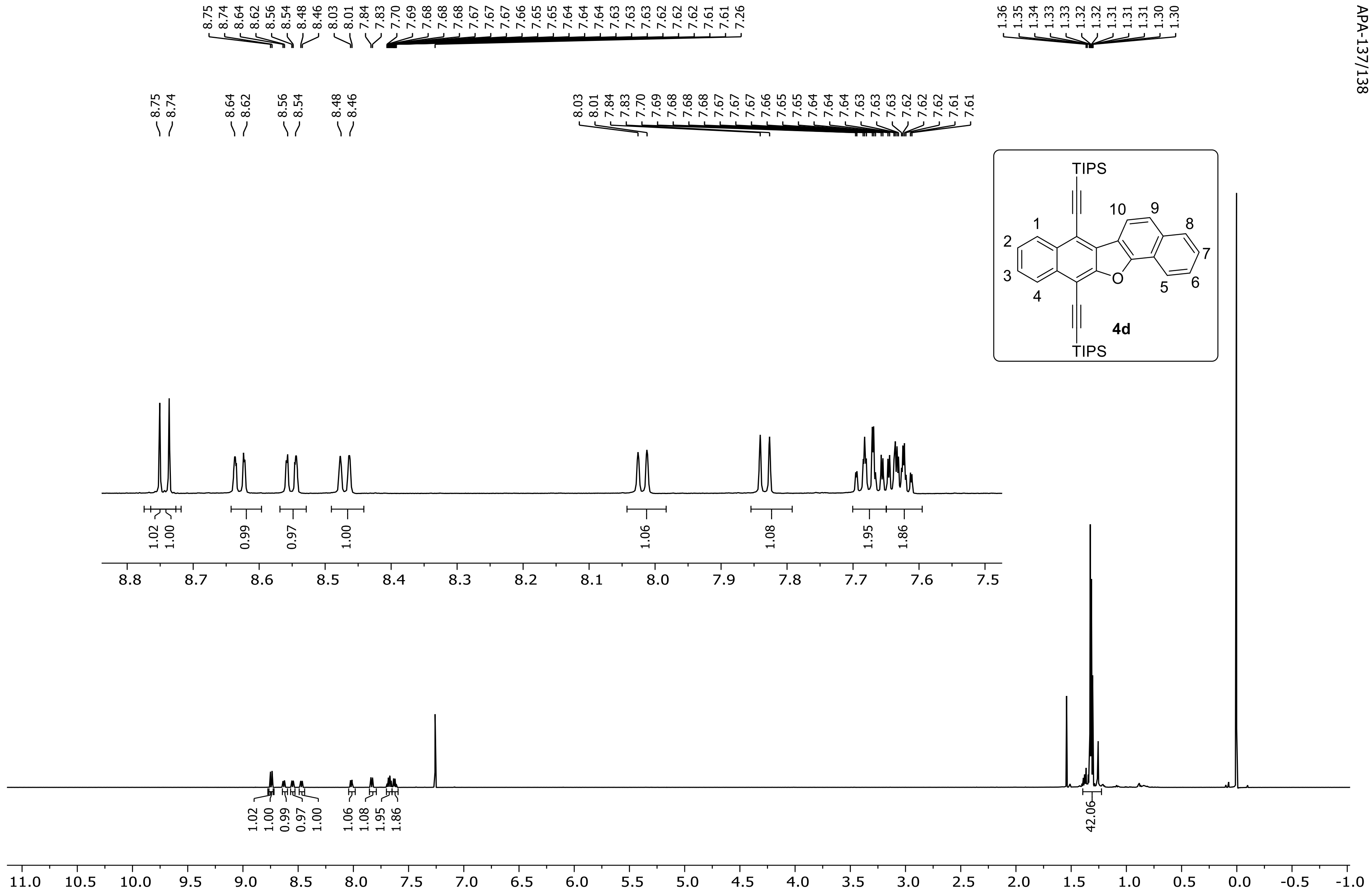

Figure S 68. ^{1}H NMR (top) spectra of **4d** in $CDCl_3$ at 600 MHz.

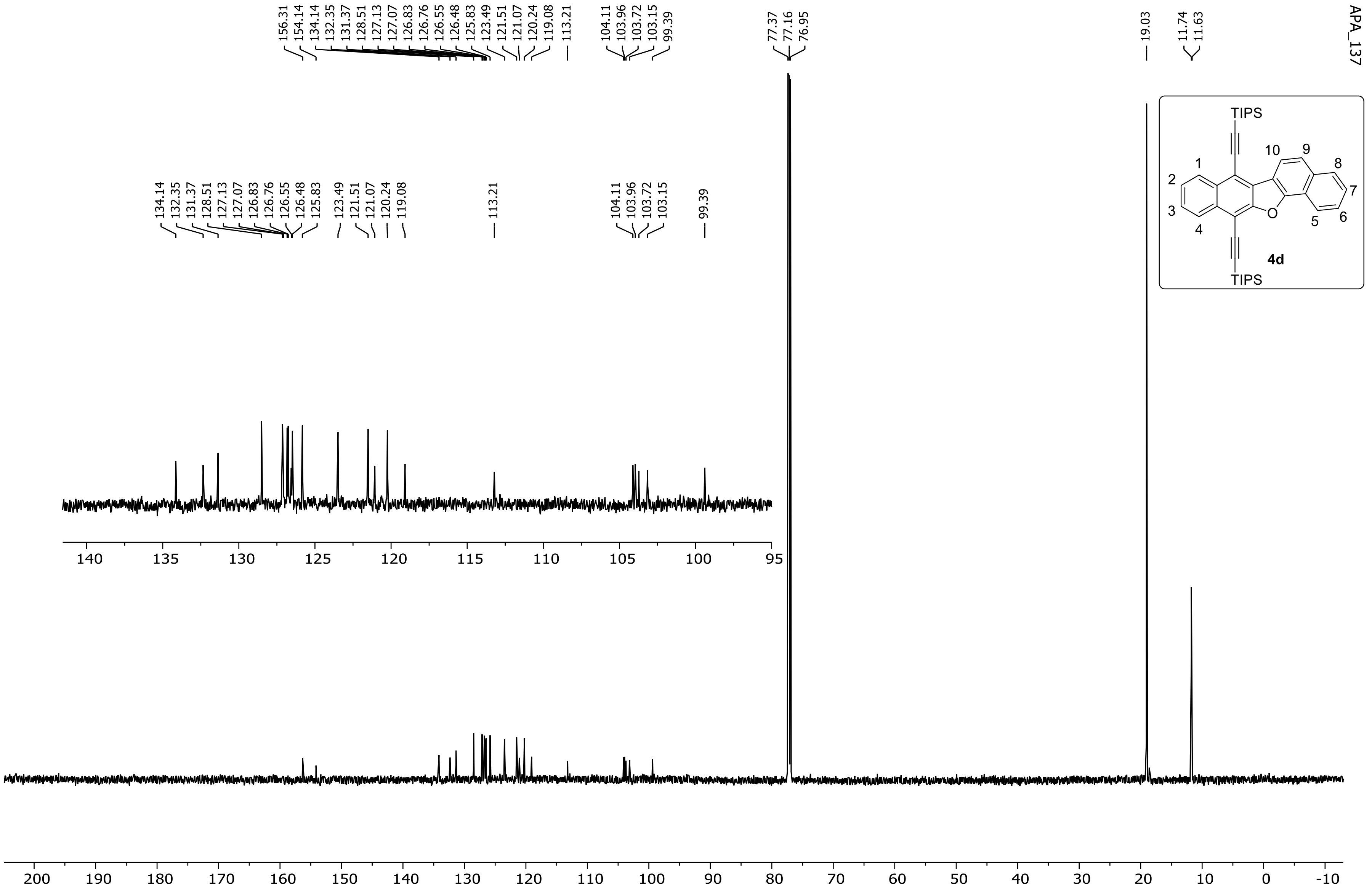

Figure S 69. $^{13}C\{^{1}H\}$ NMR (top) spectra of **4d** in $CDCl_3$ at 151 MHz.

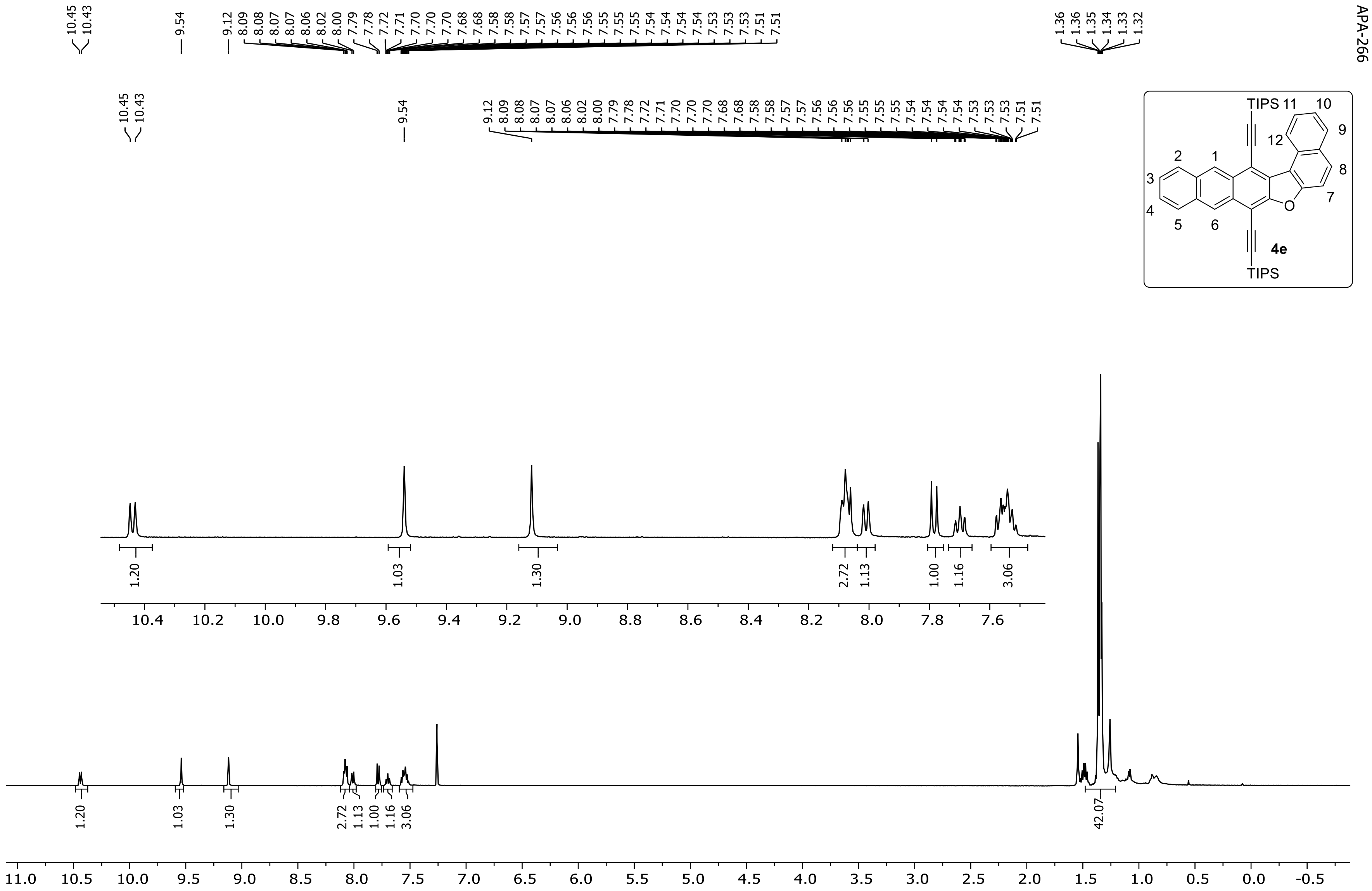

Figure S 70. ^{1}H NMR (top) spectra of **4e** in $CDCl_3$ at 500 MHz.

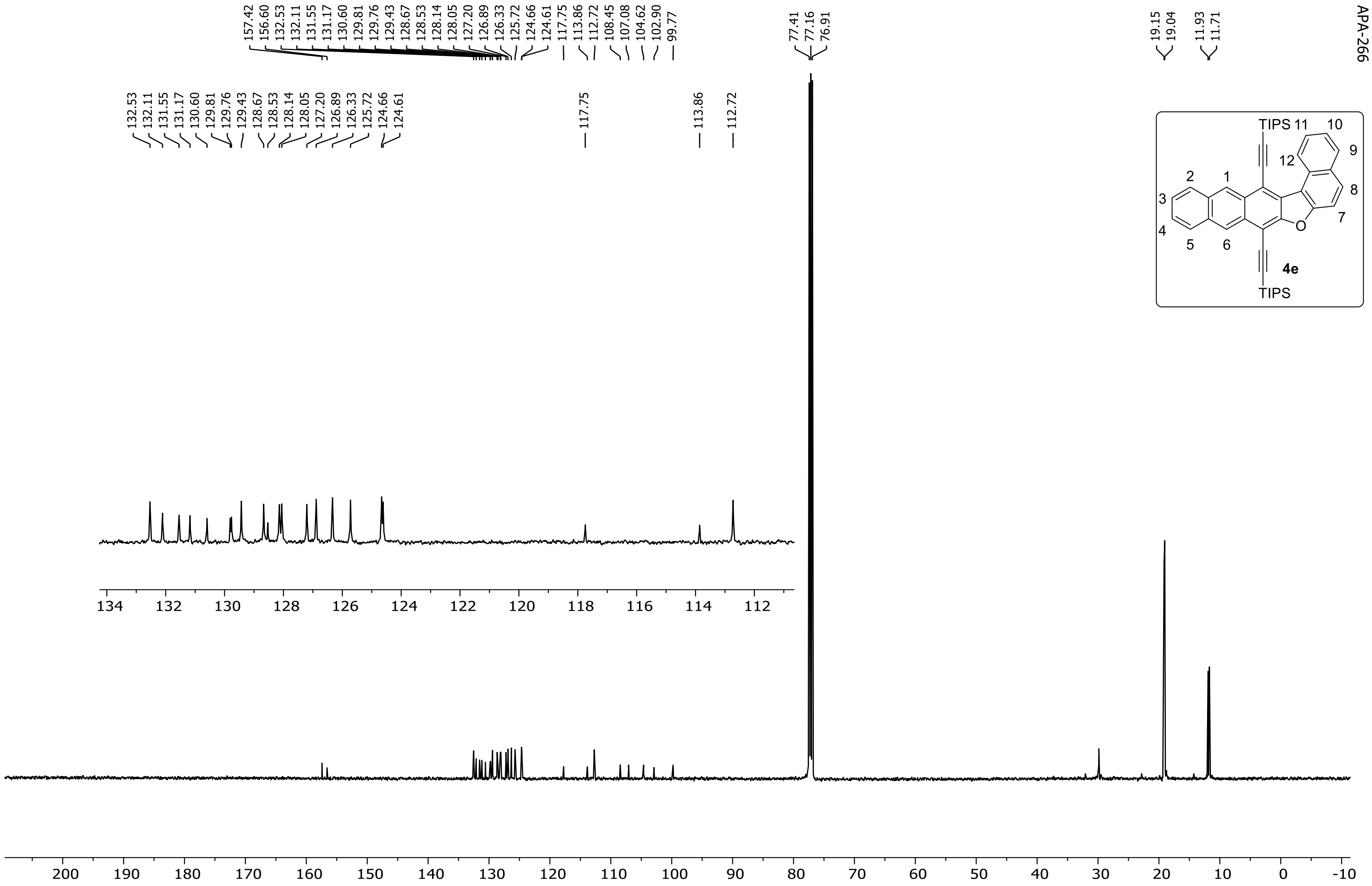

Figure S 71. $^{13}C\{^1H\}$ NMR (top) spectra of **4e** in $CDCl_3$ at 151 MHz.

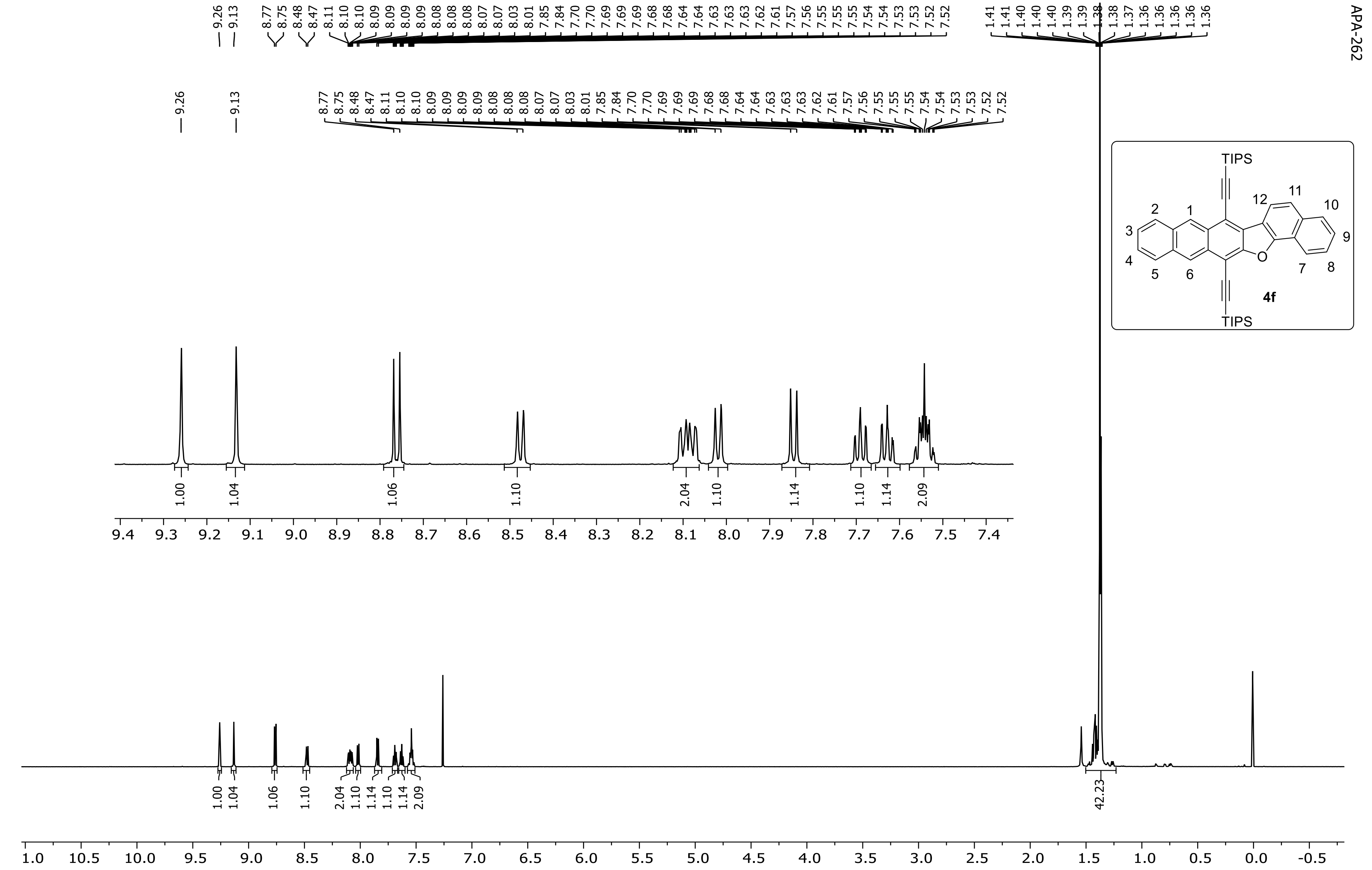

Figure S 72. ^{1}H NMR (top) spectra of **4f** in $CDCl_3$ at 600 MHz.

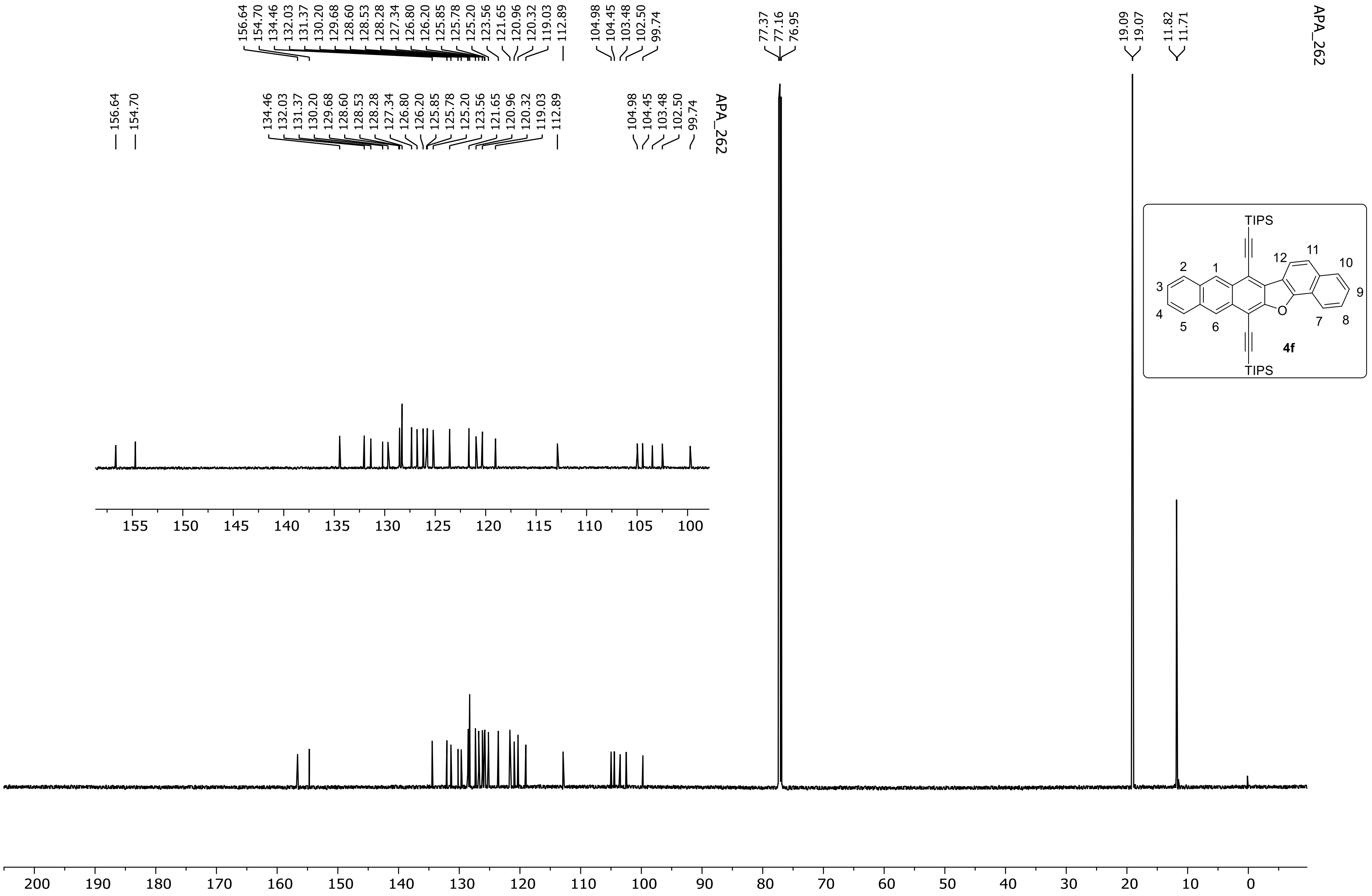

Figure S 73. $^{13}C\{^1H\}$ NMR (top) spectra of **4f** in $CDCl_3$ at 151 MHz.

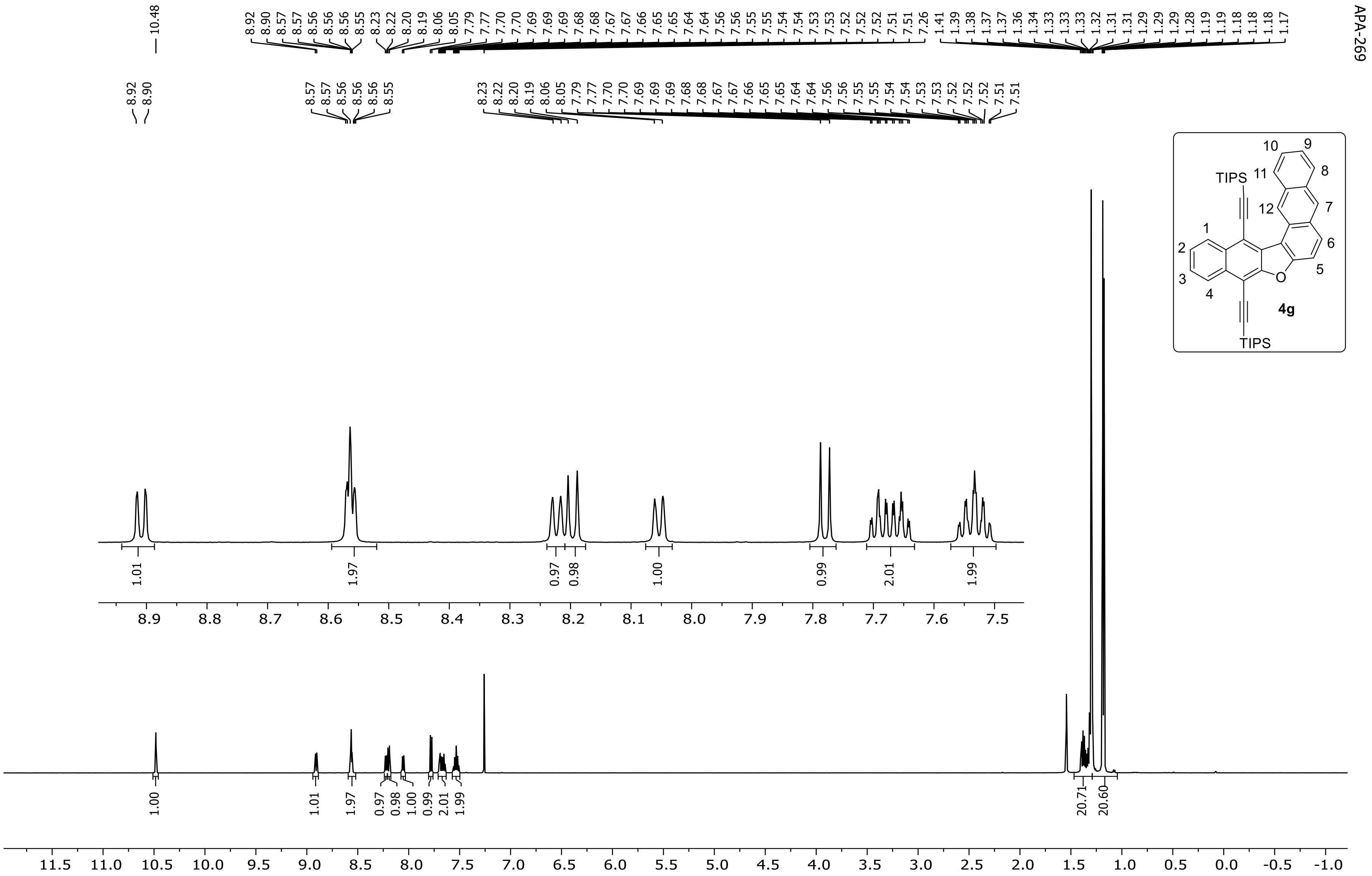

Figure S 74. ^{1}H NMR (top) spectra of **4g** in $CDCl_3$ at 600 MHz.

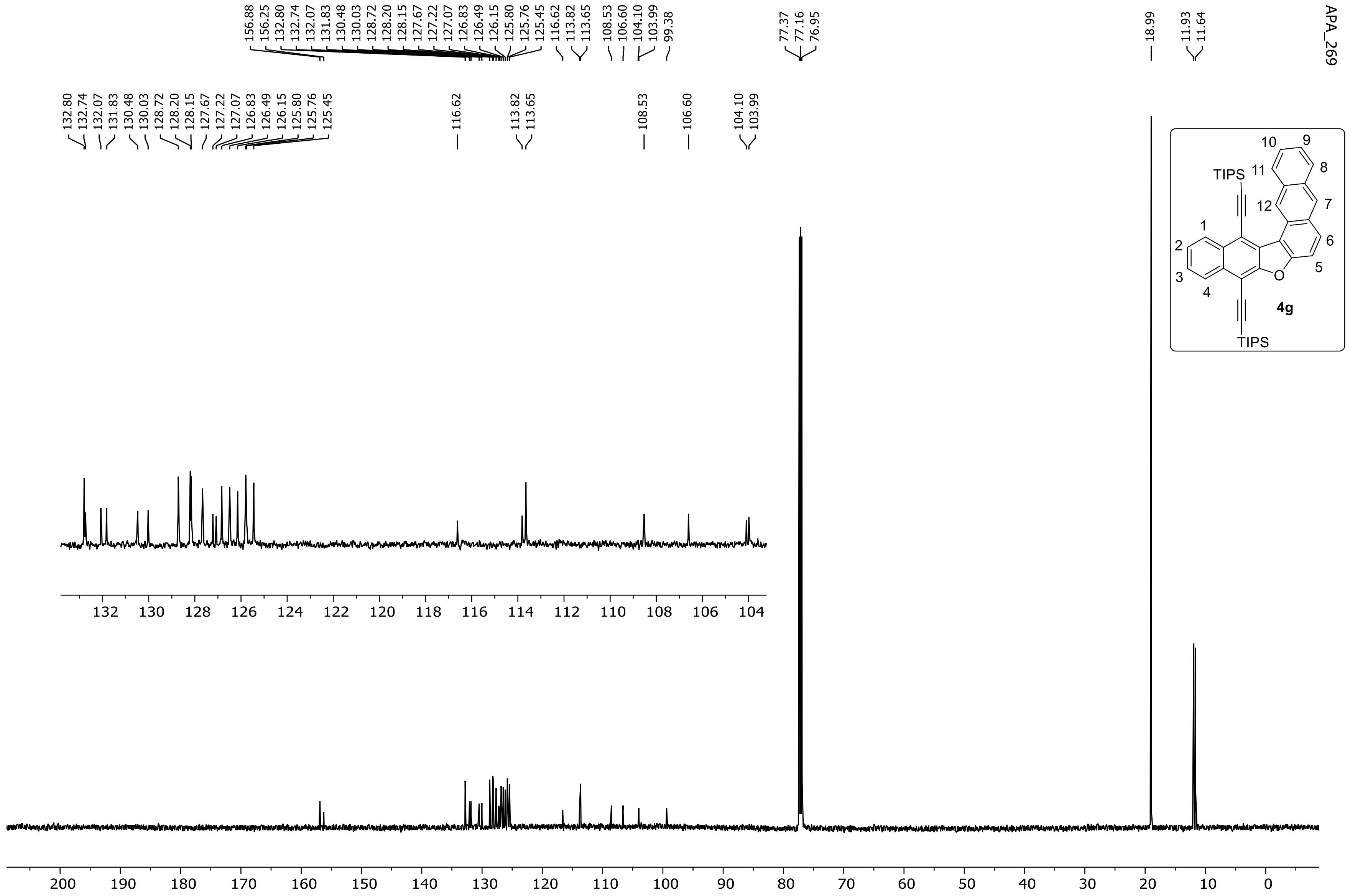

Figure S 75. $^{13}C\{^{1}H\}$ NMR (top) spectra of **4g** in $CDCl_3$ at 151 MHz.

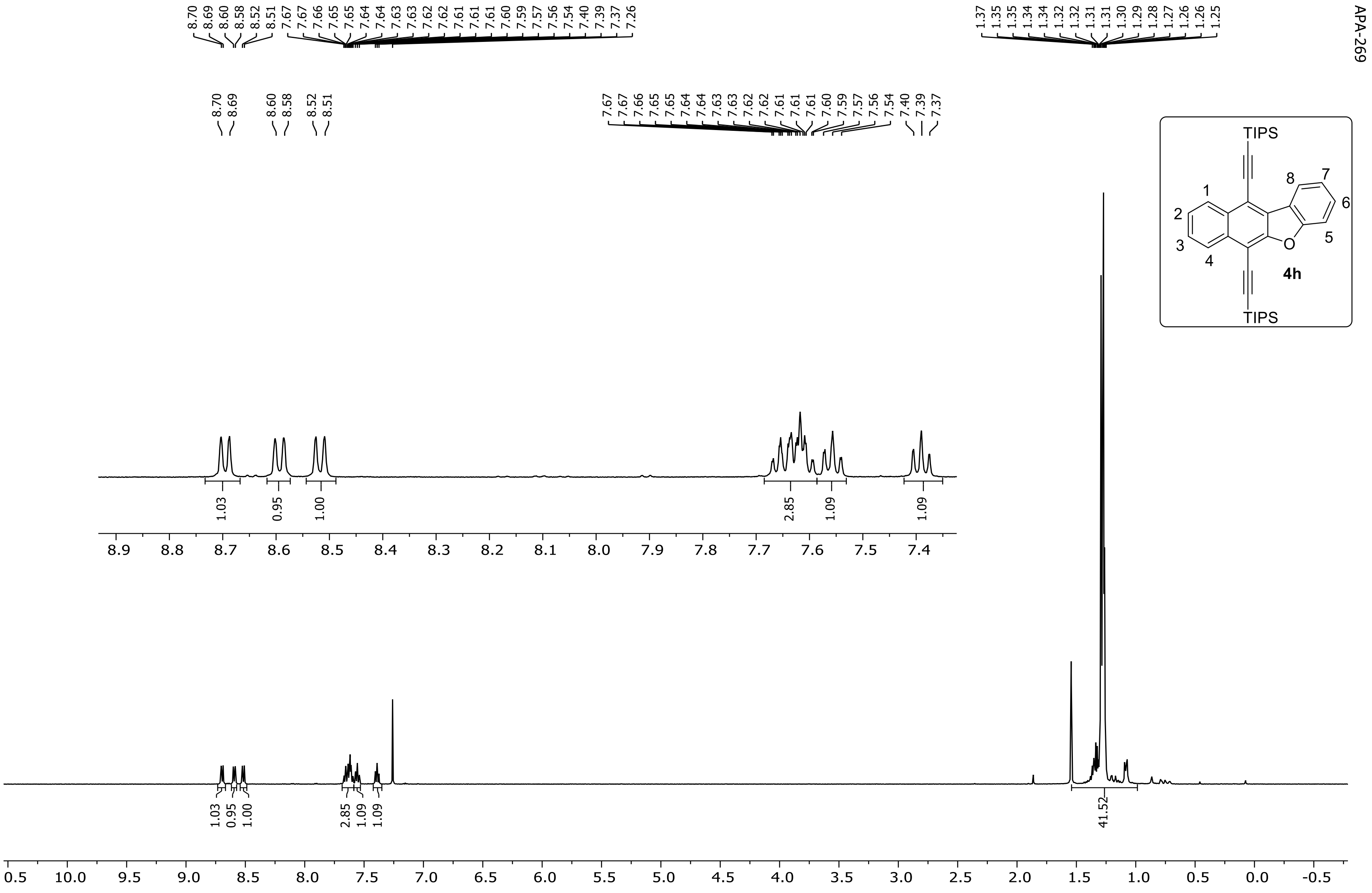

Figure S 76. ^{1}H NMR (top) spectra of **4h** in $CDCl_3$ at 500 MHz.

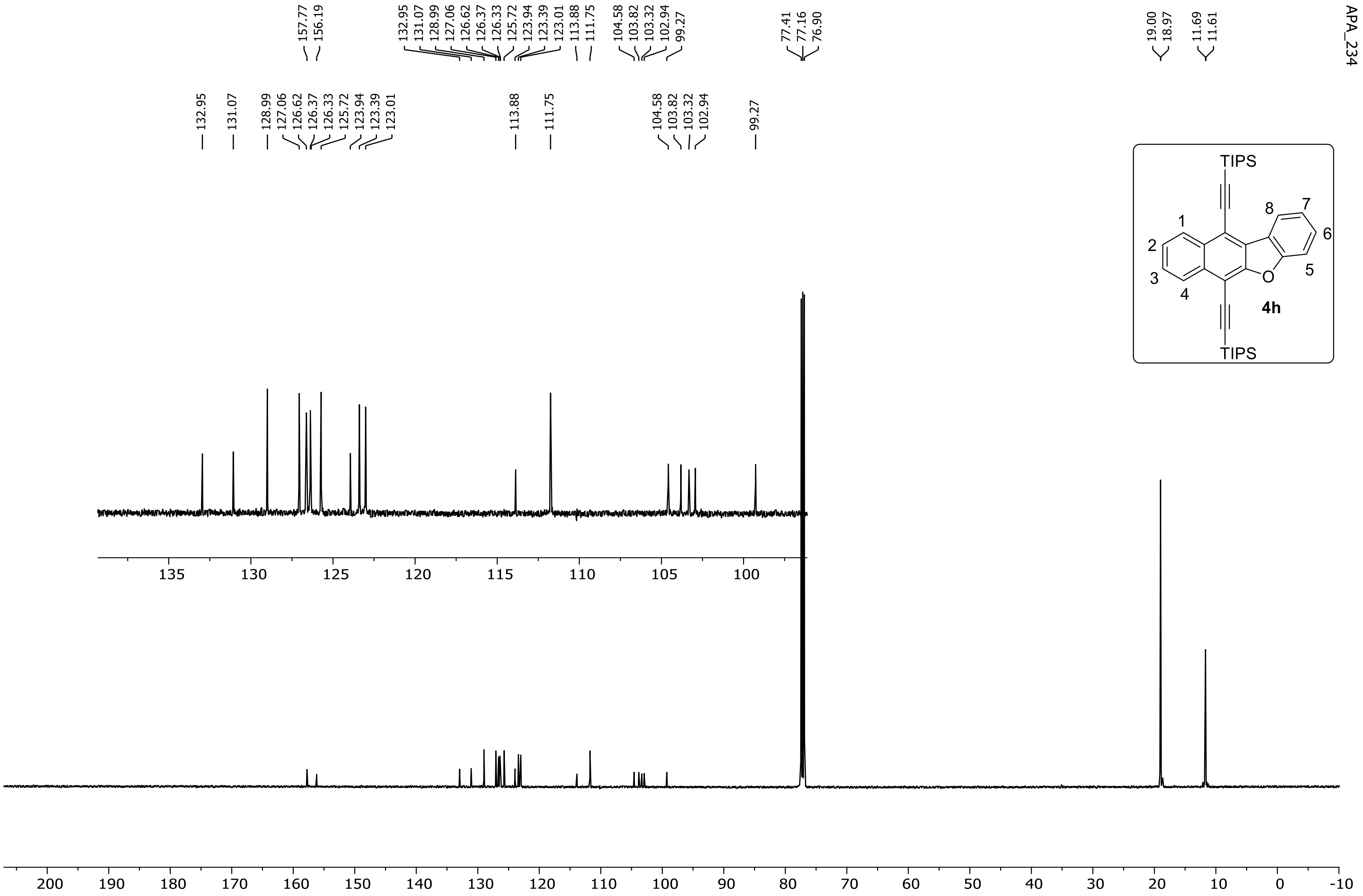

Figure S 77. $^{13}C\{^{1}H\}$ NMR (top) spectra of **4h** in $CDCl_3$ at 126 MHz.

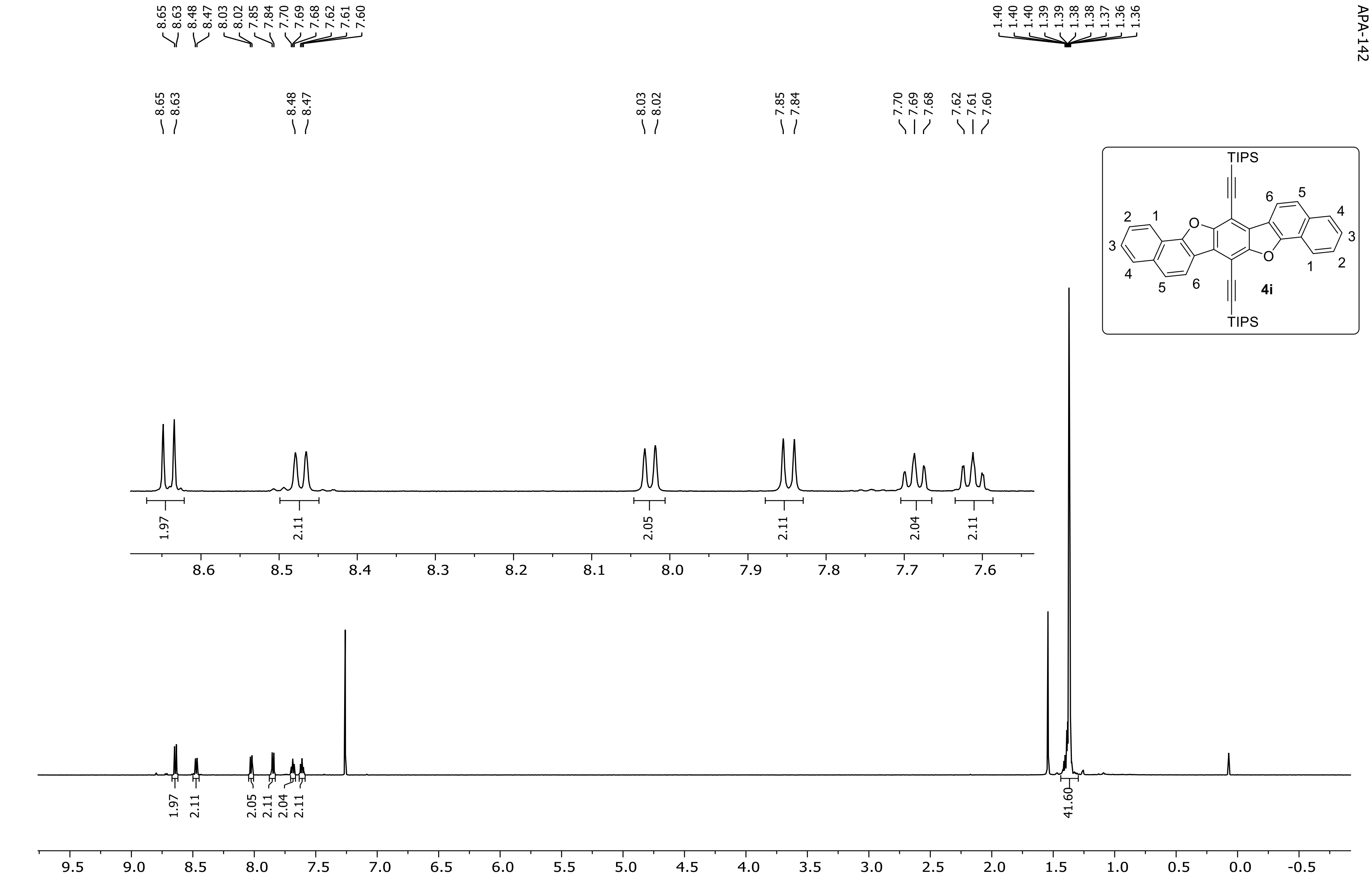

Figure S 78. ^{1}H NMR (top) spectra of **4i** in $CDCl_3$ at 600 MHz.

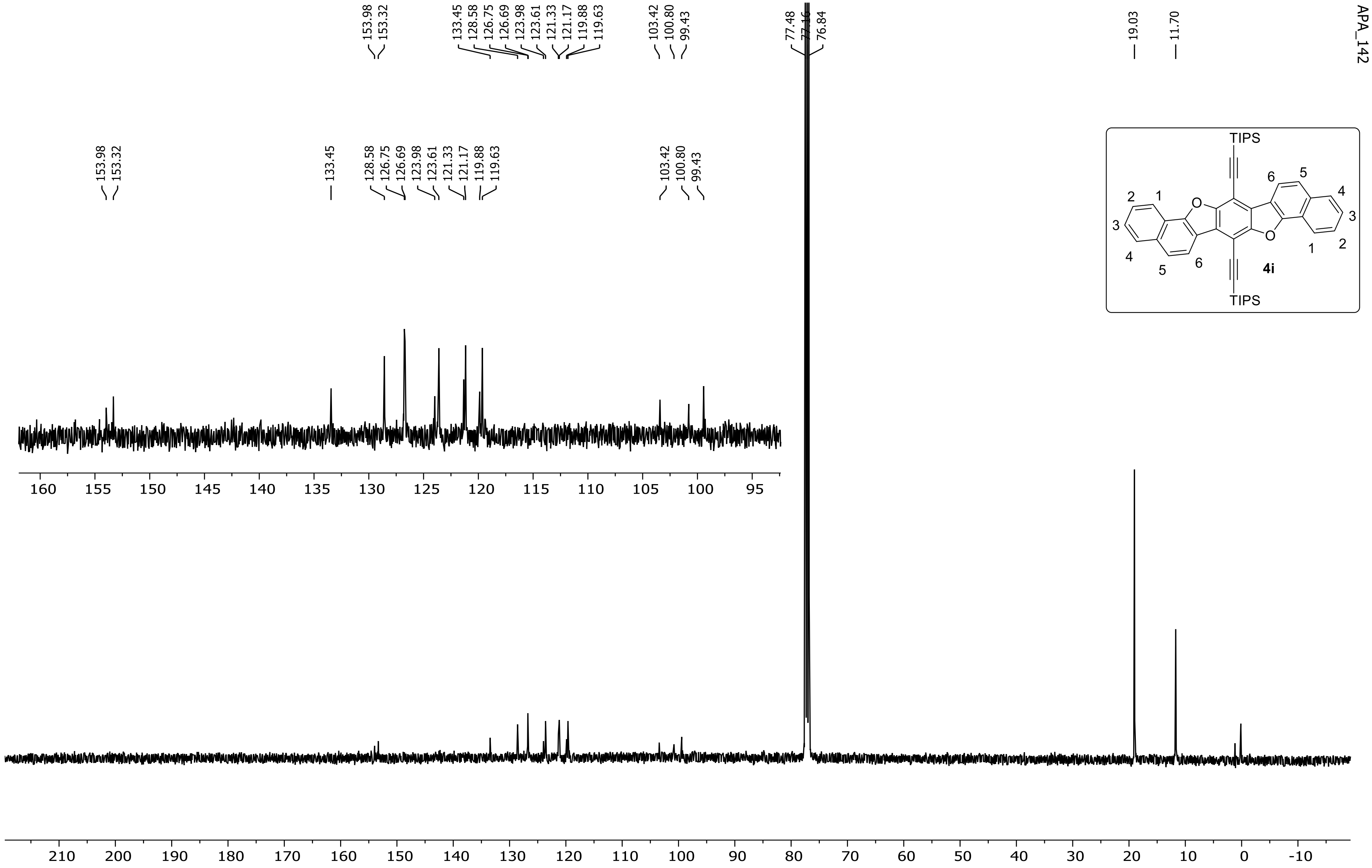

Figure S 79. $^{13}C\{^1H\}$ NMR (top) spectra of **4i** in $CDCl_3$ at 101 MHz.

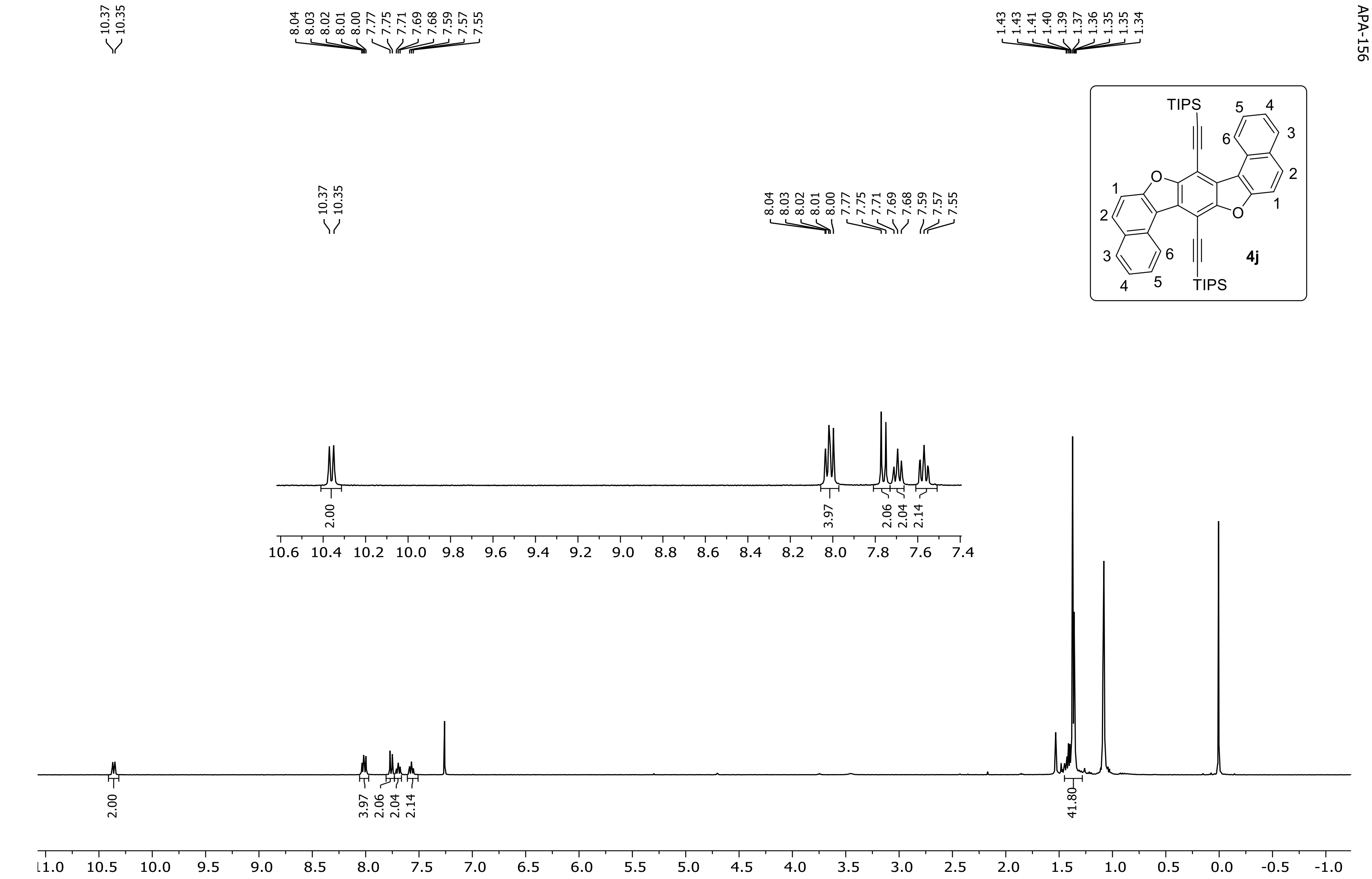

Figure S 80. ^{1}H NMR (top) spectra of **4j** in $CDCl_3$ at 400 MHz.

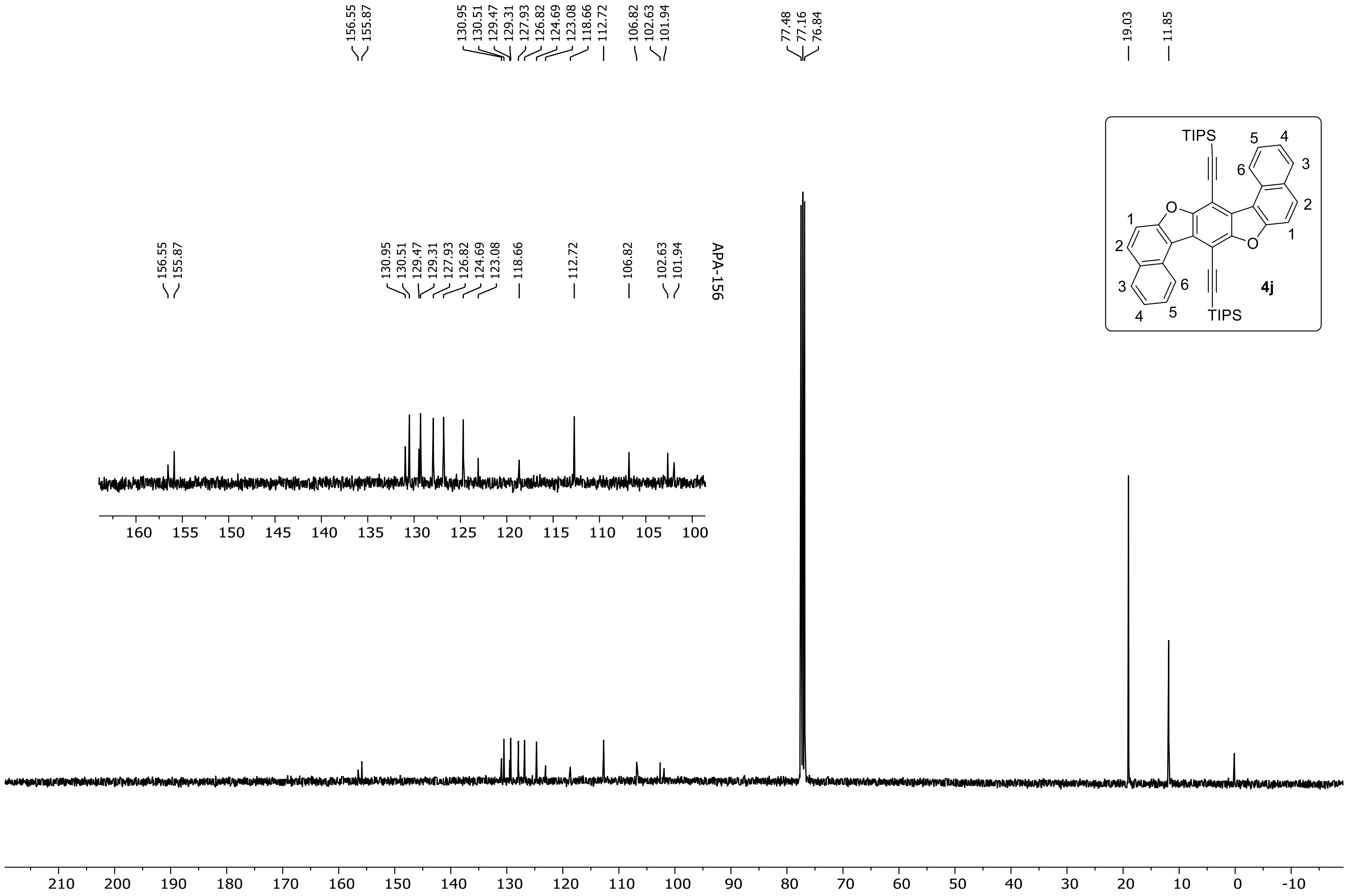

Figure S 81. $^{13}C\{^1H\}$ NMR (top) spectra of **4j** in $CDCl_3$ at 101 MHz.

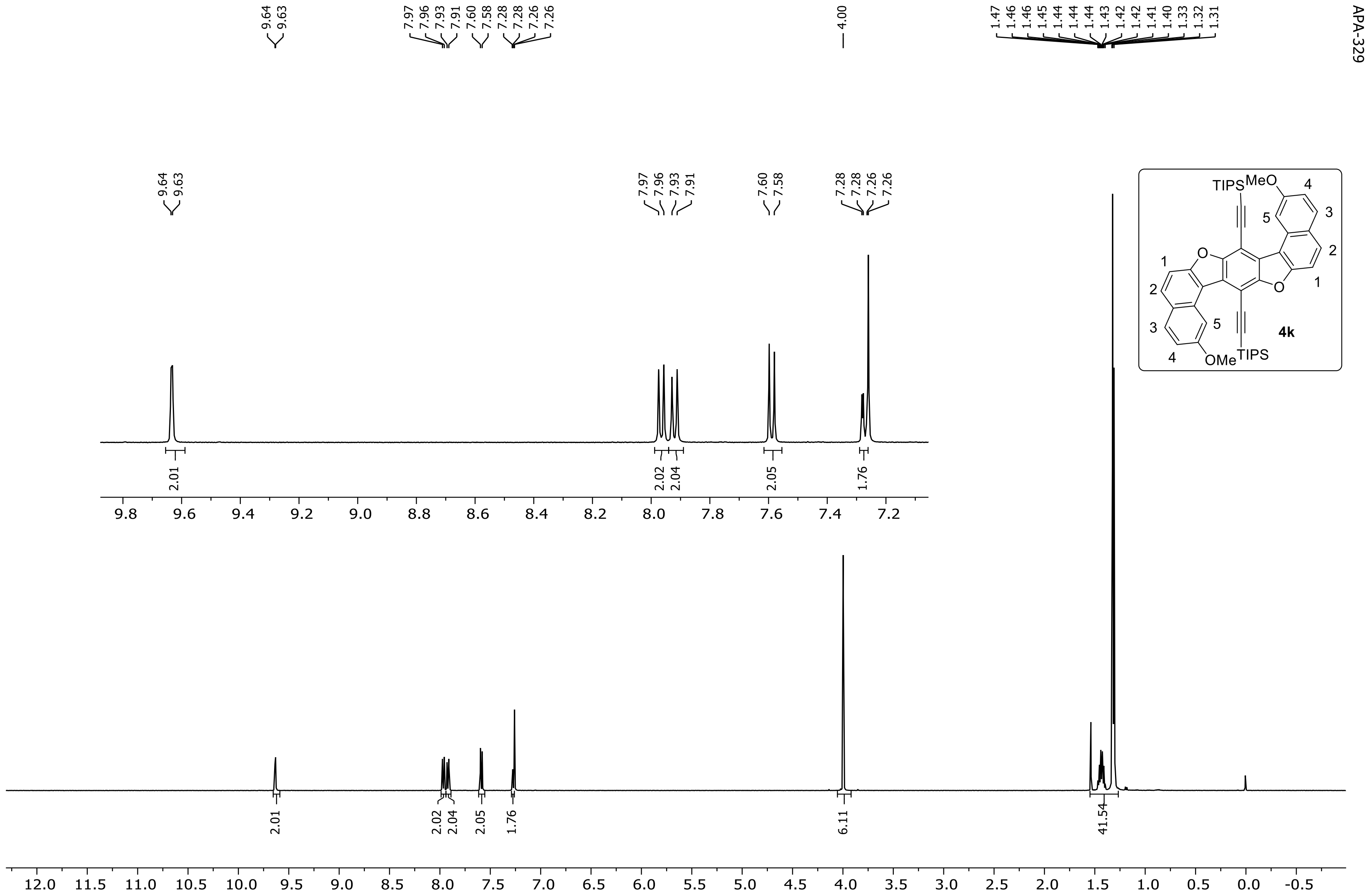

Figure S 82. ^{1}H NMR (top) spectra of **4k** in $CDCl_3$ at 500 MHz.

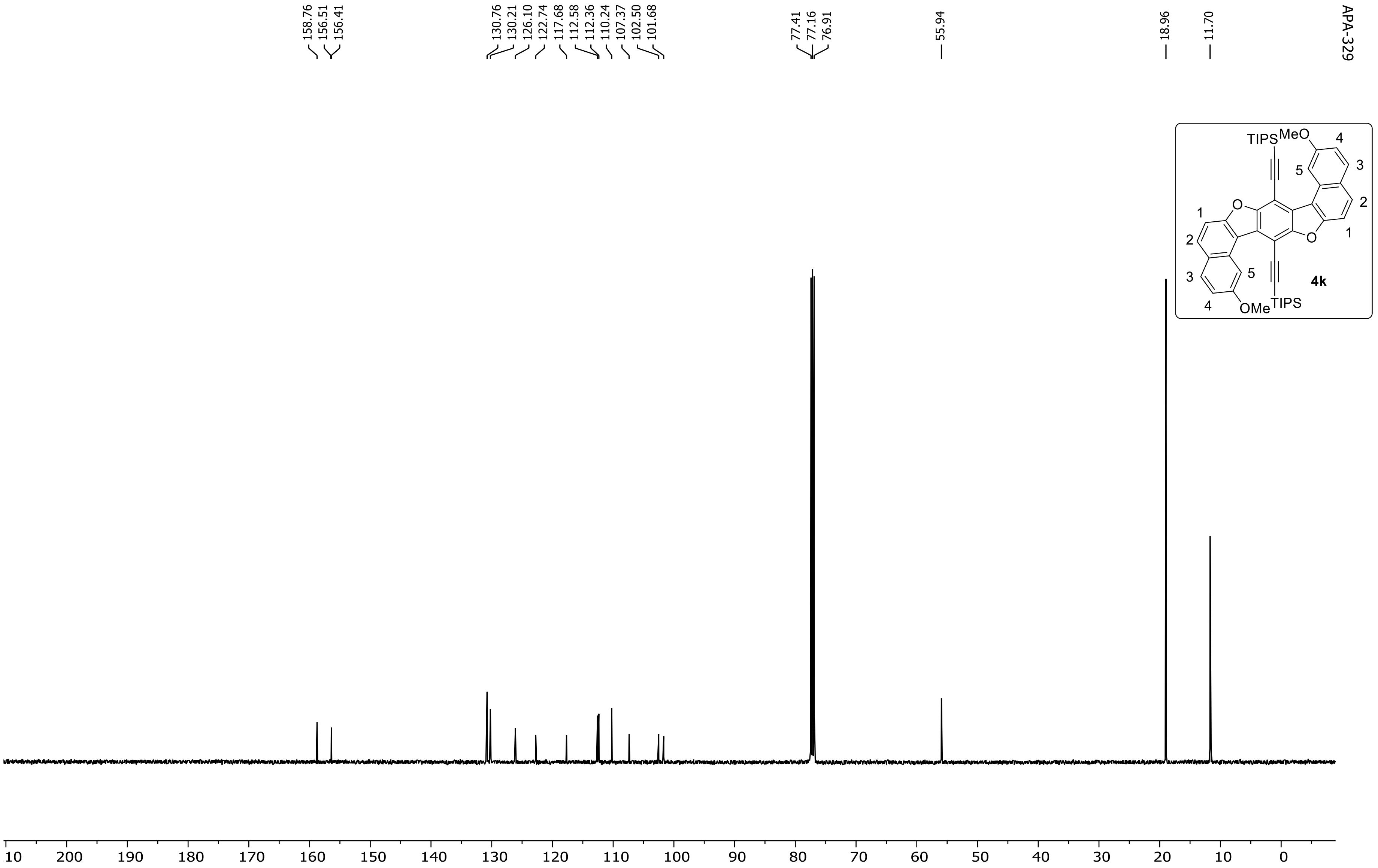

Figure S 83. $^{13}C\{^1H\}$ NMR (top) spectra of **4k** in $CDCl_3$ at 126 MHz.

S 11. References

1. Williams, A. T. R.; Winfield, S. A.; Miller, J. N. Relative Fluorescence Quantum Yields Using a Computer-Controlled Luminescence Spectrometer. *Analyst* **1983**, *108*, 1067–1071.
2. Morris, J. V.; Mahaney, M. A.; Huber, J. R. Fluorescence Quantum Yield Determinations. 9,10-Diphenylanthracene as a Reference Standard in Different Solvents. *J. Phys. Chem.* **1976**, *80*, 969–974.
3. Bruker AXS Inc. *Bruker*; Madison, WI, 2004.
4. Sheldrick, G. A Short History of SHELX. *Acta Crystallogr., Sect. A* **2008**, *64*, 112–122.
5. Sheldrick, G. M. *SADABS. Program for Empirical Absorption Correction of Area Detector Data*; University of Göttingen: Göttingen, Germany, 1996.
6. Sheldrick, G. M. *SHELXL-2014. Program for the Refinement of Crystal Structures from Diffraction Data*; University of Göttingen: Germany, 2014.
7. Pochorovski, I.; Boudon, C.; Gisselbrecht, J.-P.; Ebert, M.-O.; Schweizer, W. B.; Diederich, F. Quinone-Based, Redox-Active Resorcin[4]arene Cavitands. *Angew. Chem., Int. Ed.* **2012**, *51*, 262–266.
8. Škalamera, Đ.; Veljković, J.; Ptiček, L.; Sambol, M.; Mlinarić-Majerski, K.; Basarić, N. Synthesis of Asymmetrically Disubstituted Anthracenes. *Tetrahedron* **2017**, *73*, 5892–5899.
9. Gu, B.; Yu, X.; Xu, Z.; Pan, F.; Wang, D. A Single-Step Palladium-Catalysed Synthesis of Naphtho[2,3-b]Benzofuran-6,11-Diones and 2-(Hydroxyphenyl)Naphthalene-1,4-Diones. *J. Chem. Res.* **2017**, *41*, 564–568.
10. Yanai, T.; Tew, D. P.; Handy, N. C. A New Hybrid Exchange–Correlation Functional Using the Coulomb-Attenuating Method (CAM-B3LYP). *Chem. Phys. Lett.* **2004**, *393*, 51–57.
11. Weigend, F.; Ahlrichs, R. Balanced Basis Sets of Split Valence, Triple Zeta Valence and Quadruple Zeta Valence Quality for H to Rn: Design and Assessment of Accuracy. *Phys. Chem. Chem. Phys.* **2005**, *7*, 3297–3305.
12. Gershoni-Poranne, R.; Stanger, A. The NICS-XY-Scan: Identification of Local and Global Ring Currents in Multi-Ring Systems. *Chem. Eur. J.* **2014**, *20*, 5673–5688.
13. Wang, Z. py.Aroma: An Intuitive Graphical User Interface for Diverse Aromaticity Analyses. *Chemistry* **2024**, *6*, 1692–1703.
